# Semisynthesis of Forsyshiyanine A and a Derivative with Significant Anti-Pancreatic Cancer Activity

**DOI:** 10.1021/acs.jnatprod.5c01178

**Published:** 2025-11-03

**Authors:** Saad Y. Rfaish, Antonio Fernández, María C. Ramos, Thomas A. Mackenzie, José Justicia, Rachid Chahboun

**Affiliations:** † Departamento de Química Orgánica, Facultad de Ciencias, Instituto de Biotecnología, 16741 Universidad de Granada, 18071 Granada, Spain; ‡ 328560 Fundación MEDINA, Av. Conocimiento 34, Health Sciences Technology Park, 18016 Granada, Spain

## Abstract

Forsyshiyanine A (**8**), a trinorditerpene alkaloid isolated from *Forsythia suspensa* exhibiting *in vitro* anti-inflammatory and antiviral activities, has been synthesized for the first time from *trans*-communic acid (**12a**) and labdane cupressic acid (**13**). Furthermore, a series of derivatives were efficiently prepared and screened for cytotoxic activities against five human tumoral cell lines. Derivative **25** showed cytotoxicity (IC_50_ = 6.5 μM) against the Mia PaCa-2 pancreatic cancer cell line, making it an interesting candidate for future structure–activity relationship (SAR) investigations.

## Graphical abstract

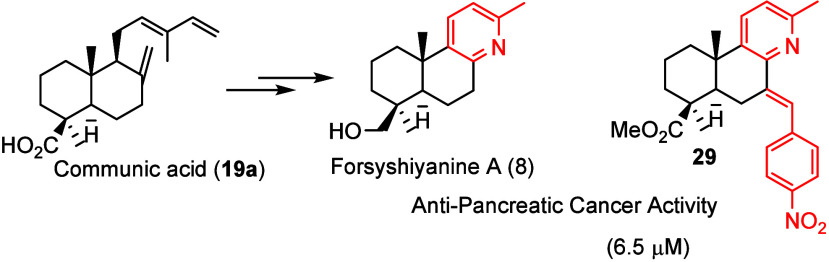

Steroidal and terpenoid alkaloids bearing a pyridinium moiety represent a relatively small but important group of metabolites characterized by their unique structures and potent biological properties.^1^ Several findings emphasize the crucial role of the pyridinium core in its biological properties. For instance, steroidal abiraterone (**1**) and its acetate derivative (**2**) are clinically used in prostate cancer therapy.^2^ Veragranine A (**3**) is another steroidal alkaloid, recently isolated from *Veratrum grandiflorum* ^3^ with a potent analgesic effect.
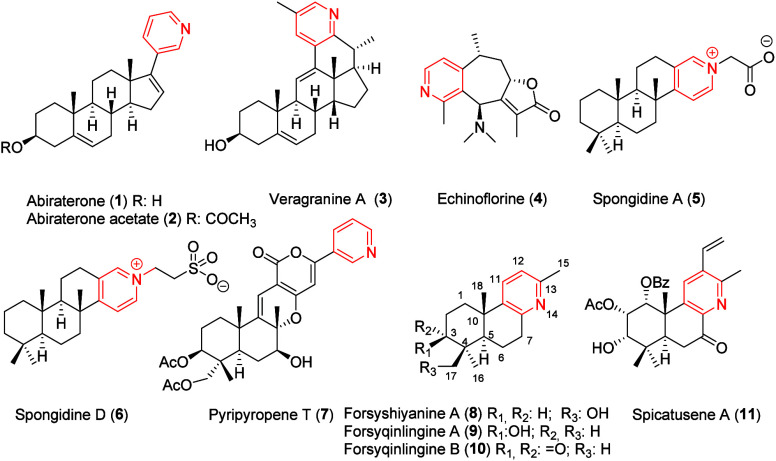
 Among the pyridinium terpenoid alkaloids include echinoflorine (**4**), a guaipyridine-type alkaloid from the gorgonian *Echinogorgia flora*,^4^ with promising biological activity, and spongidines A (**5**) and D (**6**), with potent inhibitory activity against phospholipase A_2_ (sPLA_2_).^5^ Semisynthesis of **5** and **6** from (+)-sclareolide has been completed.^6^ Pyripyropene T (**7**), a marine pyridinium meroterpenoid alkaloid isolated from *Veratrum grandiflorum*, has shown strong inhibitory activity against acyl-CoA:cholesterol acyltransferase (ACAT).^7^ Furthermore, recent studies have highlighted the biological potential of other pyridinic terpenoid alkaloids, such as forsyshiyanine A (**8**) and forsyqinlingines A (**9**) and B (**10**), isolated from *Forsythia suspensa*.^8^ Spicatusene A (**11**), isolated from *Clerodendranthus spicatus*,^9^ and structurally related to forsyshiyanine A (**8**) underscores the pharmacological relevance of this emerging class of compounds.

## Results and Discussion

Owing to the remarkable therapeutic potential of forsyshiyanine (**8**) and the growing demand for cost-effective and readily accessible bioactive compounds, we propose the synthesis of **8** along with a series of selected derivatives as the main objective of this work. To this end, naturally abundant labdane diterpenes *trans*-communic acid (**12a**) and cupressic acid (**13**), were selected as starting materials due to their widespread occurrence in different sources,^10^ but mainly in Cupressaceae family plants. Isolation studies of *trans*-communic acid (**12a**) from *Cupressus sempervirens* species revealed that this acid is present in different proportions with cupressic acid (**13**) depending on the season, tree variety, and collection site of the species. Thus, in specimens harvested in December 2023 from northern Granada, Spain, *trans*-communic acid (**12a**) and cupressic acid (**13**) were obtained at ∼2.3% and ∼1% of the plant weight, respectively. Both compounds possess the *trans*-decalinic core characteristic of forsyshiyanine A (**8**), making them ideal precursors for the semisynthesis of **8**. The pyridinic ring can be constructed by suitable transformation of the side chain and exocyclic double bond of cupressic acid (**13**) to afford diketone **14**. In addition, this moiety can be prepared via selective oxidation of the side chain of communic acids (**12a**–**c**) to yield alcohol **16**, which can be readily converted into methyl ketone **15**. Accordingly, compound **8** can be efficiently accessed from diketone **14** in a few straightforward steps (see Scheme 1).

**Scheme 1:**
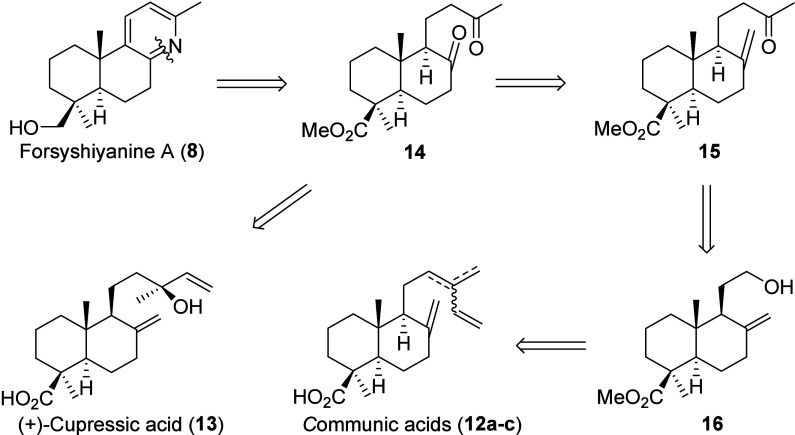
Proposed Retrosynthesis of Forsyshiyanine A (**8**) from Communic and Cupressic Acids (**12a**–**c** and **13**)

The synthesis of methylketone **15** from *trans*-communic acid (**12a**) was first explored to implement the proposed retrosynthetic sequence. Previous approaches employed a 15:35:50 mixture of (*trans*, *cis*, and *myrceo*) communic acids (**12a**–**c**), isolated from *Juniperus communis*, for the synthesis of **15** (see Scheme 2).^11^ However, only the *myrceo* isomer **12c** efficiently afforded methylketone **15**.^11a^ Alternatively, a more straightforward and efficient route to this key intermediate **15** is the direct and selective oxidative degradation of cupressic acid methyl ester.

**Scheme 2:**
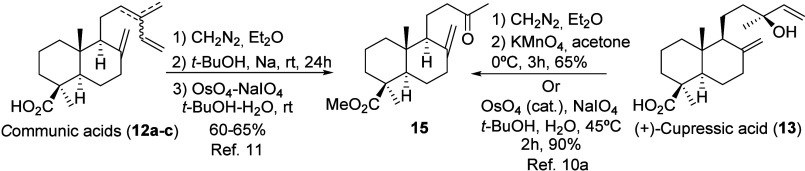
Described Synthesis of Methylketone **15** from **12a**–**c** and **13**

Our study proposes a new route to **15** from methyl *trans*-communate (**17**). The synthetic sequence comprises four basic steps: selective oxidative cleavage of the Δ^12*E*^ double bond with ozone, reduction of the resulting ozonide with NaBH_4_ to yield alcohol **16**,^10b^ its conversion into nitrile **19** via the corresponding iodo derivative **18**, and final transformation to methylketone **15** through MeMgBr addition and subsequently acid hydrolysis (see Scheme 3). The proposed synthetic route is general and versatile, enabling the synthesis of forsyshiyanine A (**8**) as well as a range of structurally related derivatives by varying the Grignard reagent in the final step.

**Scheme 3:**
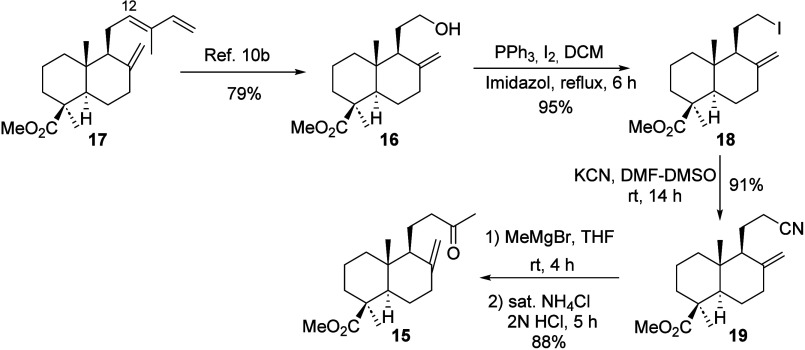
Synthesis of Methylketone **15** from *trans*-Communic Acid Methyl Ester **17**

Once the synthesis of **15** was performed, it was oxidized with ozone to afford diketone **14**, a key precursor for the target compound. Alternatively, diketone **14** can be directly prepared by oxidation of (+)-cupressic acid methyl ester (**20**) with KMnO_4_ in acetone at room temperature. However, this reaction provided only moderate yields on a gram scale. Significant portion of diketone **14** underwent further reaction with oxygen under alkaline conditions, leading to byproducts such as ketophenol **21**. Alternatively, **20** was oxidized employing the RuCl_3_(cat)/NaIO_4_ system at room temperature, affording diketone **14** in 76% yield along with 19-methylester ambracetal (**22**) (17%) as a byproduct.^11d^ The most efficient oxidation of **20** to diketone **14** was achieved using KMnO_4_ in acetone at room temperature in the presence of AcOH, affording the corresponding diketone **14** in 84% yield (see Scheme 4).

**Scheme 4:**
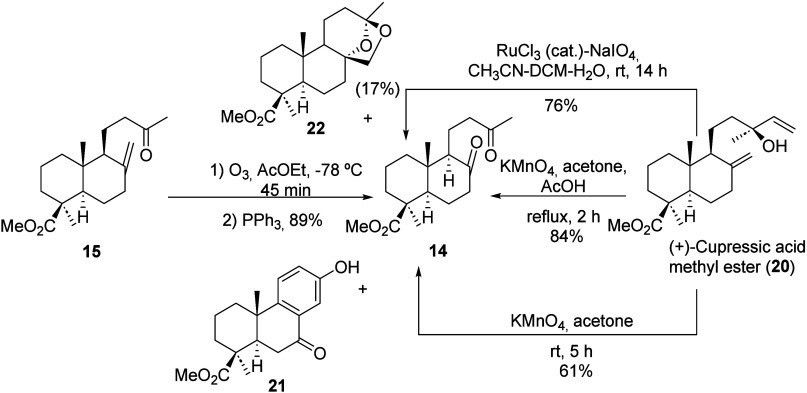
Synthesis of Diketone **14** from (+)-Cupressic Acid Methyl Ester (**20**) and Methylketone **15**

Finally, diketone **14** was converted into forsyshiyanine A (**8**) in high yield by treatment with the NH_4_OAc/Cu­(OAc)_2_ system in EtOH,^12^ followed by the reduction of the methyl ester in the tricyclic intermediate **23** using LiAlH_4_, as shown in Scheme 5. This approach enabled the synthesis of natural product **8** in only three steps from compound **20**, with a 73% global yield (or seven steps and 50% overall yield from **17**). These results highlight the efficiency and versatility of the proposed synthetic route. The spectroscopic data of synthetic **8** matched those reported for the natural product.^8a^

**Scheme 5:**
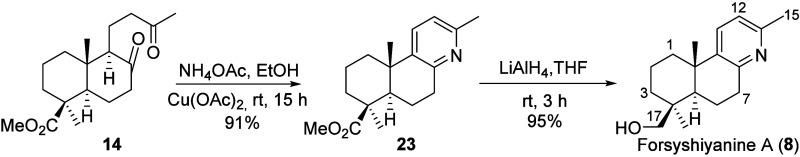
Synthesis of Forsyshiyanine A (**8**)

The concise synthetic strategy developed herein provided rapid access to the 17-methyl ester derivative of forsyshiyanine A (**8**). Therefore, the adjacent carbons to the pyridyl ring (C-7 and Me-15) in intermediate **23** would allow the design and synthesis of a series of derivatives through oxidative functionalization reactions. In addition, intermediate **19** can serve as a versatile precursor for the preparation of further derivatives by applying the same synthetic strategy used for **8**, substituting the Grignard reagent with alternative derivatives. Both routes enable the generation of a library of forsyshiyanine A (**8**) derivatives for the exploration of their biological activities. In light of these considerations, we propose the preparation of 17-methyl ester derivatives of forsyshiyanine A (**8**) as the next objective of this study.

First, the functionalization of Me-15 in compound **23** was studied. Treatment of **23** with NBS in the presence of substoichiometric amount of benzoyl peroxide under reflux in chlorobenzene unexpectedly afforded a 7:3 mixture of epimers: 7α-benzoyloxy derivative **24a** and 7β**-**benzoyloxy derivative **24b** in low yield, rather than the expected bromination of Me-15. Using stoichiometric benzoyl peroxide improved the yield of **24a**–**b** to 64%. Attempt to functionalize Me-15 via aldol-type condensation of **23** with *p*-nitrobenzaldehyde in Ac_2_O/AcOH under reflux conditions selectively produced compound **25**. The formation of this product likely proceeds via intermediate (**I**) (see Scheme 6). Similarly, treatment of **23** with aqueous KMnO_4_ in acetone at reflux yielded 7-ketopyridine **26**. These results underscore the difficulty of Me-15 functionalization in forsyshiyanine A (**8**), indicating that C-7 is the more reactive site and can be selectively modified. Successful functionalization of Me-15 was achieved using benzeneseleninic anhydride, affording ketoaldehyde **27** in high yield after 45 min under reflux in chlorobenzene. The oxidation of benzylic carbons using (PhSeO)_2_O has been previously reported.^13^ Subsequent Pinnick oxidation of **27** afforded the picolinic acid derivative **28** in a good yield (Scheme 6). Overall, this synthetic strategy enabled the efficient synthesis of complex, functionalized derivatives of natural **8** in only three steps from **20**, with good global yields.

**Scheme 6:**
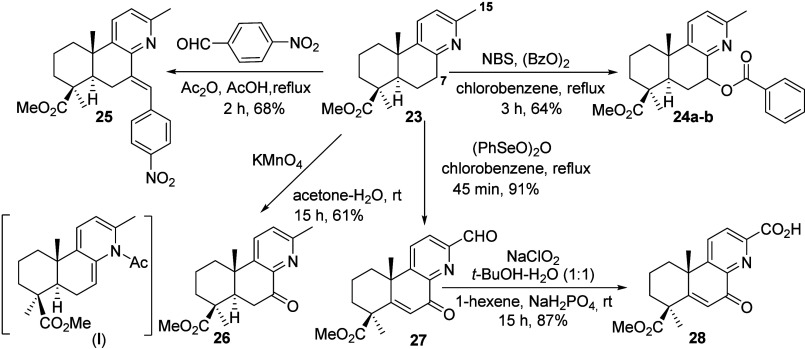
Synthesis of Forsyshiyanine A Derivatives **24**–**28**

An alternative proposal for the synthesis of functionalized derivatives of **8** involved modification of the terpenic skeleton prior to the pyridine ring formation. As an illustrative example, the 13-aryl-derivative **31** was prepared from intermediate **19** following the synthetic route established for forsyshiyanine A (**8**). The sequence involved the addition of 4-methoxyphenylmagnesium chloride to **19**, followed by acid hydrolysis, ozonolysis of the exocyclic double bond, and the formation of compound **31** via treatment with the NH_4_OAc/Cu­(OAc)_2_ system in EtOH (see Scheme 7).

**Scheme 7:**
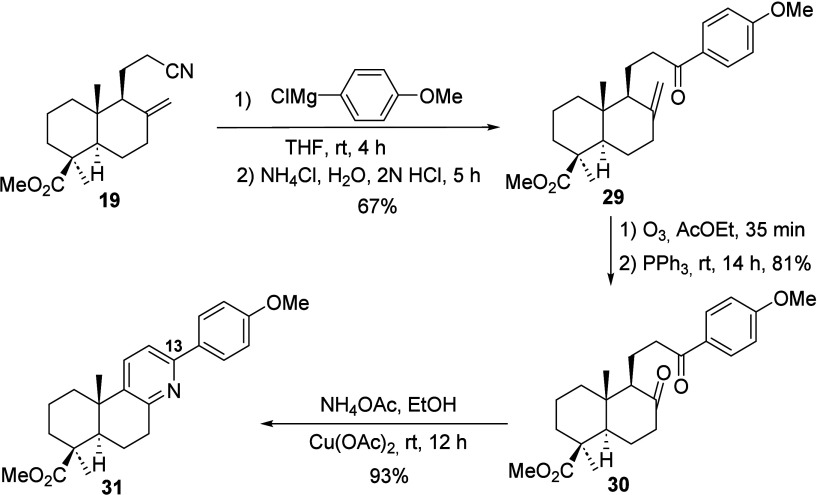
Synthesis of the 13-Aryl Forsyshiyanine A-Related Compound **31**

This synthetic route allows rapid access to derivatives of **8** in only three steps from intermediate **19**, with a good overall yield. The simplicity and efficiency of proposed approaches demonstrate that a broad array of **8** derivatives can be prepared in a straightforward and versatile manner.

As noted in the introduction, *Forsythia suspensa* is recognized in Chinese pharmacopoeia for its anti-inflammatory, antiviral, and anticancer properties. Forsyshiyanine A (**8**) has shown notable anti-inflammatory and antiviral activity. In this study, the anticancer potential of forsyshiyanine A (**8**) and its derivatives was evaluated against five human cancer cell lines, including A549 lung adenocarcinoma, A2058 melanoma, HepG2 hepatocellular carcinoma, MCF7 breast cancer, and Mia PaCa-2 pancreatic carcinoma. The cytotoxic activity of these compounds was first assayed using a cell-based MTT assay.^14^ Methylmethanesulfonate (MMS) was used as positive control. The resulting cytotoxicity percentages are summarized in Table 1.

**Table 1:** *In Vitro* Cytotoxicity Percentage of Compounds **8**, **23**–**28**, and **31** in Five Different Cell Lines^a^

	A549 (lung)	A2058 (melanoma)	HepG2 (hepatoma)	MCF-7 (breast)	Mia PaCa-2 (pancreas)
Compound	% Cytotoxicity	% Cytotoxicity	% Cytotoxicity	% Cytotoxicity	% Cytotoxicity
**8**	9.2 ± 0.8	7.5 ± 0.3	2.5 ± 5.7	9.1 ± 2.1	0.9 ± 10.8
**23**	14.2 ± 0.9	11.9 ± 0.8	22.6 ± 3.3	16.6 ± 1.9	19.3 ± 14.3
**24a**–**b**	5.9 ± 5.0	4.9 ± 0.1	–1.7 ± 4.8	19.7 ± 2.0	11.0 ± 11.3
**25**	5.8 ± 2.2	42.8 ± 0.8	71.2 ± 0.0	51.4 ± 6.3	84.9 ± 2.5
**26**	27.1 ± 4.3	8.7 ± 0.5	4.3 ± 6.4	36.3 ± 4.0	7.8 ± 11.6
**27**	–1.9 ± 0.2	2.4 ± 0.3	1.9 ± 4.9	2.5 ± 3.0	–1.0 ± 6.0
**28**	–4.5 ± 0.1	2.6 ± 0.6	–4.3 ± 4.0	–7.5 ± 9.5	–5.5 ± 5.5
**31**	8.6 ± 5.4	68.2 ± 10.0	53.2 ± 0.9	66.4 ± 4.8	72.6 ± 3.3
MMS	99.65 ± 0.2	99.81 ± 0.1	100.1 ± 0.1	99.83 ± 0.2	99.86 ± 0.1

a Compounds were tested at 50 μM. Data are expressed as mean ± standard deviation.

Compounds **25** and **31** showed >50% activity (84.9% and 72.6%, respectively) against Mia PaCa-2. These compounds were subsequently tested at different doses per triplicate to generate dose–response curves and determine the IC_50_ values (50% inhibitory concentration), as summarized in Table 2. IC_50_ values could only be calculated for these compounds against the pancreatic Mia PaCa-2 cell line, as no notable activity was observed in the other tested cell lines. The corresponding dose–response curves are presented in Figure 1.

**Table 2:** *In Vitro* Cytotoxicity Study of Compounds **25** and **31** in Five Different Cell Lines^a^

	A549 (lung)	A2058 (melanoma)	HepG2 (hepatoma)	MCF-7 (breast)	Mia PaCa-2 (pancreas)
Compound	IC_50_ (CI95%) μM	IC_50_ (CI95%) μM	IC_50_ (CI95%) μM	IC_50_ (CI95%) μM	IC_50_ (CI95%) μM
**25**	>100	>100	>100	>100	6.5 (4.2–9.9)
**31**	>100	>100	>100	>100	53.2 (40.4–69.9)
Doxorubicin	0.19 (0.1–0.2)	0.19 (0.1–0.2)	0.09 (0.1–0.2)	0.12 (0.1–0.2)	0.11 (0.0–0.1)

a Data are expressed as the 50% inhibitory concentration (IC_50_) and 95% confidence interval (CI95).

**Figure 1:**
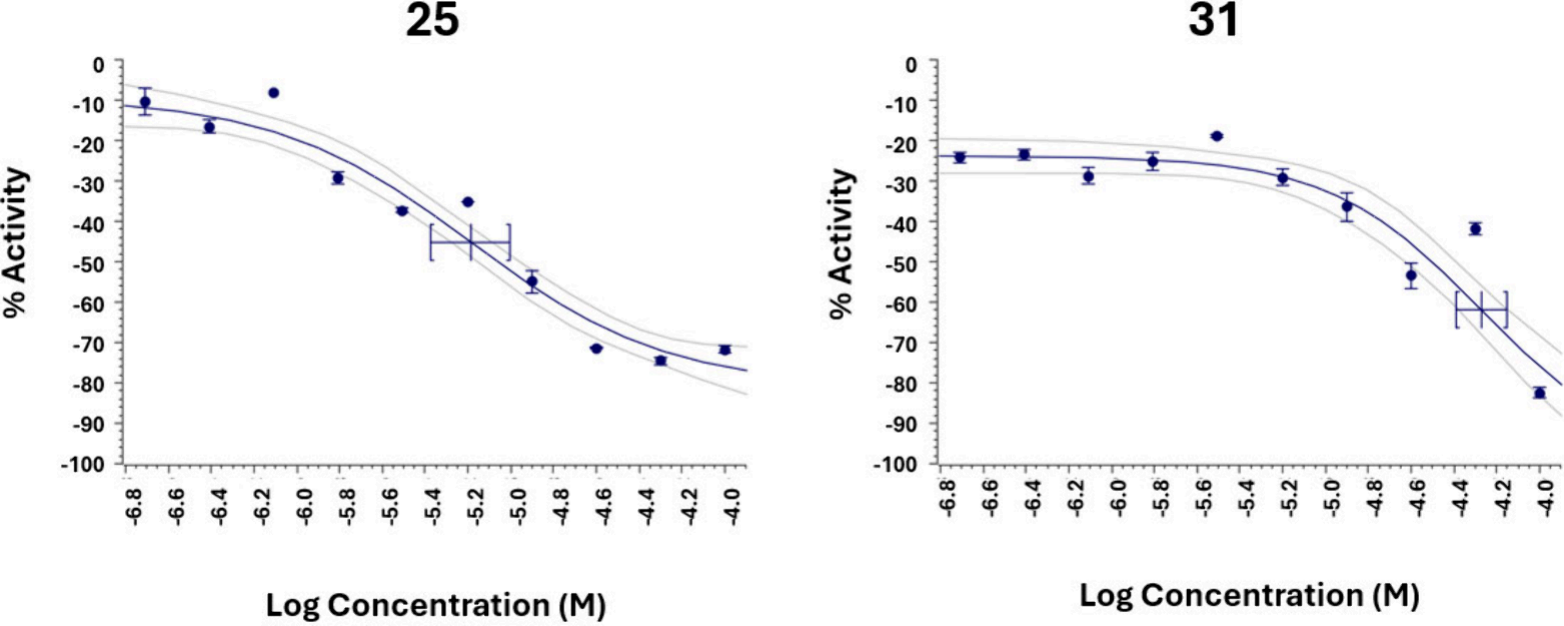
Dose–response curves of compounds **25** and **31** against the pancreatic cell line Mia PaCa-2.

Cytotoxicity results against the Mia PaCa-2 pancreatic cell line are promising. Compound **25** showed a IC_50_ activity at 6.5 μM, while derivative **31** presented no activity (IC_50_ of 53.2 μM). Structurally, the activity appears to be linked to the existence of conjugated aromatic rings, present in compounds **25** and **31** but absent in the other derivatives, which showed negligible effects. Other functionalizations, such as carbonyl groups at C-7, C-15, or C-17, did not enhance cytotoxicity.

The straightforward and flexible synthesis of these compounds enables the introduction of diverse structural variations at early stages. This fact would allow the preparation of broad derivative libraries for further biological evaluation and potential optimization.

In summary, we have achieved the first three-step semisynthesis of forsyshiyanine A (**8**) from cupressic acid (**13**) in 73% overall yield. In addition, an alternative route from *trans*-communic acid (**12a**), abundant in *Cupressus sempervirens*, was also performed. This synthesis confirmed the structure and absolute stereochemistry of natural product **8**, and enable access to diverse analogues, including C-13 aryl and alkyl derivatives and C-7 oxidized congeners. Biological evaluation revealed that compounds **25** and **31** display cytotoxicity against pancreatic cancer cells (IC_50_ = 6.5 μM and 53.2 μM, respectively). These results underscore the potential of these short and versatile synthetic routes to generate readily accessible derivatives with promising antitumor activity.

## Experimental Section

### General Experimental Procedures

Specific Rotation measurements were carried out in a PerkinElmer 341 polarimeter using a 1 dm length cell and CHCl_3_ as a solvent. Ultraviolet–visible spectra were obtained using an Analytikjena Specord 200 Plus spectrometer, and MeCN solutions in a 1 cm cuvette. Infrared spectra (IR) were recorded as thin films or solids on a PerkinElmer model One FTIR spectrophotometer, with samples between NaCl plates or as KBr pellets and are reported in frequency of absorption (cm^–1^). ^1^H and ^13^C NMR spectra were recorded on Varian instruments (at 400 or 500 MHz and 100 or125 MHz, respectively). CDCl_3_ was treated with K_2_CO_3_. The chemical shifts are expressed in parts per million (ppm) and are referred to CHCl_3_ (7.26 ppm) in the ^1^H NMR spectra and CDCl_3_ (77.16 ppm) in the ^13^C NMR spectra. HRMS were recorded on a Waters Xevo G2-XS QTof spectrometer using Q-TOF analyzer and ESI^+^ ionization. Thin-layer chromatography (TLC) was performed using E. Merck silica gel 60 F254 precoated plates (0.25 mm) and visualized by UV fluorescence quenching and phosphomolybdic acid solution staining. Flash chromatography was performed on silica gel (Merck Kieselgel 60, 230–400 mesh). Chromatography separations were carried out by conventional column on silica gel 60 (230–400 Mesh), using EtOAc/Hexane mixtures of increasing polarity. Unless otherwise stated, the reactions were performed in oven-dried glassware under an argon atmosphere using dry solvents. Compounds **8**,^8a^ **12a**–**c**,^11a^ **15**,^11a^ **14**,^11d^ **16**,^10b^ **17**,^10b^ and **20** ^10a^ are known in literature.

### General Procedures for the Preparation of **14** and **26**

To a solution of compounds **20** or **23** (1 mmol) in acetone (10 mL for the synthesis of **20**) or acetone:AcOH (10:2 mL for the synthesis of **20**) or acetone:H_2_O (10:2 mL for the synthesis of **26**) at 0 °C, KMnO_4_ (5 mmol for preparation of **14** or 3 mmol for preparation of **26**) was added in several portions, and the mixture was stirred at room temperature or at reflux for the specific time indicated in Schemes 4 and 6. Then, a 5% solution of NaHSO_3_ (2.5 mL) was added, and the reaction mixture was stirred for a further 15 min. Next, it was filtered, washed with acetone (2 × 5 mL), and the solvent was removed under vacuum to afford a crude product, which was diluted with EtOAc:H_2_O (15:5 mL), and the phases were shaken and separated. The organic phase was washed with H_2_O (3 × 5 mL), brine (2 × 5 mL), dried over anhydrous Na_2_SO_4_, filtered, and the solvent was removed. The residue was purified by flash silica gel chromatography (AcOEt/Hexane mixtures), giving compounds **14** or **26** in the yields indicated in Schemes 4 and 6.

### General Procedures for the Preparation of **23** and **31**

To a solution of **14** or **30** (1 mmol) in EtOH (15 mL), Cu­(OAc)_2_ (1.5 mmol) and NH_4_OAc (4 mmol) were added, and the reaction mixture was stirred at room temperature for the specific time. Then, the mixture was filtered through a silica gel pad, and the solvent was removed. The obtained residue was diluted with EtOAc:H_2_O (30:10 mL), and the phases were shaken and separated. The organic layer was washed with H_2_O and brine, dried over anhydrous Na_2_SO_4,_ filtered, and the solvent was removed. The residue was purified by flash chromatography on silica gel (30% EtOAc/Hexane) to give **23** or **31** in the yields indicated in Schemes 5 and 7.

### Synthesis Procedures

#### Methyl (1*S*,4a*R*,­5*S*,8a*R*)-5-(2-Iodo­ethyl)-1,4a-di­methyl-6-meth­yl­ene­deca­hydro­naph­thal­ene-1-car­boxy­late (**18**)

To a solution of PPh_3_ (2.62 g, 10 mmol) in CH_2_Cl_2_ (30 mL) iodide (2.54 g, 10 mmol) was added, and the mixture was stirred at room temperature for 5 min. Then, imidazole (1 g, 14.7 mmol) was added, and the mixture was stirred for another 10 min. Next, a solution of **16** (2.16 g, 7.71 mmol) in CH_2_Cl_2_ (20 mL) was added, and the mixture was refluxed for 6 h. Then, the solvent was removed under vacuum, and the crude product was diluted with EtOAc:H_2_O (60:20 mL), and the phases were shaken and separated. The organic layer was washed with H_2_O (2 × 20 mL) and brine (20 mL), dried over anhydrous Na_2_SO_4,_ and the solvent was removed. The residue (6.2 g) was purified by flash chromatography on silica gel (5% EtOAc/Hexane) giving **18** (2.85 g, 95%) as a colorless syrup.

#### Methyl (1*S*,4a*R*,­5*S*,8a*R*)-5-(2-Cyano­ethyl)-1,4a-di­methyl-6-meth­yl­ene­deca­hydro­naph­thal­ene-1-carb­oxy­late (**19**)

To a solution of **18** (1.97 g, 5.05 mmol) in DMF:DMSO (30:20 mL) KCN was added (0.7 g, 10.77 mmol), and the mixture was stirred at room temperature for 14 h. Then, H_2_O:EtOAc (30:80 mL) was added and the phases were shaken and separated. The organic phase was washed with H_2_O (5 × 20 mL), brine (1 × 20 mL), dried over anhydrous Na_2_SO_4_, filtered, and the solvent was removed. The residue (1.6 g) was purified by flash chromatography on silica gel (5% EtOAc/Hexane) giving **19** (1.47 g, 91%) as a white solid.

#### Methyl (1*S*,4a*R*,­5*S*,8a*R*)-1,4a-Di­methyl-6-meth­ylene-5-(3-oxo­but­yl)­deca­hydro­naph­thal­ene-1-carb­oxy­late (**15**)

To a solution of **19** (915 mg, 3.16 mmol) in dry THF (20 mL) at 0 °C, 1 M solution of methylmagnesium bromide in THF (4.5 mL, 4.5 mmol) was added carefully under an argon atmosphere, and the resulting mixture was stirred at room temperature for 4 h. Then, a saturated solution of NH_4_Cl (5 mL) and 2 N HCl (5 mL) were added successively at 0 °C, and the reaction mixture was stirred for 5 h. Next, the solvent was removed under vacuum, and the mixture was diluted with EtOAc (40 mL). The organic layer was washed with H_2_O (2 × 10 mL) and brine (20 mL), dried over anhydrous Na_2_SO_4,_ filtered, and the solvent was removed. The residue (1.2 g) was purified by flash chromatography on silica gel (5% EtOAc/Hexane) yielding 851 mg (88%) of an unresolvable mixture of methylketone **15** and **19** (ratio 5:1).

#### Methyl (1*S*,4a*S*,­5*R*,8a*R*)-1,4a-Di­methyl-6-oxo-5-(3-oxo­but­yl)­deca­hydro­naph­thal­ene-1-carb­oxy­late (**14**) from Cupressic Acid Methyl Ester (**20**)

##### a. By Treatment with KMnO_4_ in Acetone

To a solution of cupressic acid methyl ester (**20**) (1.3 g, 3.89 mmol) in acetone (40 mL) at 0 °C, KMnO_4_ (3 g, 18.98 mmol) was added in several portions, and the mixture was stirred at room temperature for 5 h. Following the same workup used in the general procedure and after column chromatography, using 5% EtOAc/Hexane, diketone **14** was obtained (730 mg, 61%) as a colorless syrup.

##### b. By Treatment with KMnO_4_ in Acetone-AcOH

To a solution of cupressic acid methyl ester (**20**) (1 g, 3 mmol) in acetone-AcOH (30:6 mL) at 0 °C, KMnO_4_ (3 g, 18.98 mmol) was added in several portions, and the mixture was stirred at reflux for 2 h. Following the same workup used in the general procedure and after column chromatography, using 5% EtOAc/Hexane, diketone **14** was obtained (776 mg, 84%) as a colorless syrup.

##### c. By Treatment with RuCl_3_/NaIO_4_

Cupressic acid methyl ester (**20**) (530 mg, 1.58 mmol) was dissolved in CH_2_Cl_2_ (5 mL) and a mixture of CH_3_CN–H_2_O (5:8 mL) was added. Then, NaIO_4_ (1.36 g, 6.35 mmol) and RuCl_3_·3H_2_O (100 mg, 0.38 mmol) were added sequentially and the dark mixture was vigorously stirred at room temperature for 14 h. Next, the organic solvents were removed under vacuum, and the crude product was diluted with EtOAc:H_2_O (25:5 mL) and the phases were shaken and separated. The organic phase was washed with H_2_O (10 mL) and brine (10 mL), dried over anhydrous Na_2_SO_4,_ filtered, and the solvent was removed. The residue (510 mg) was purified by flash chromatography on silica gel (10% EtOAc/Hexane), yielding **22** (86 mg, 17%) as a white solid and **14** (370 mg, 76%) as a colorless syrup.

#### Methyl (1*S*,4a*S*,­5*R*,8a*R*)-1,4a-Di­methyl-6-oxo-5-(3-oxo­but­yl)­deca­hydro­naph­thal­ene-1-car­boxy­late (**14**) from Methylketone (**15**)

A solution of methylketone **15** (1 g, 3.26 mmol) in EtOAc (50 mL) at −78 °C was bubbled with a constant stream of O_3_/O_2_ flow for 45 min. Then, the mixture was flushed with argon and PPh_3_ (920 mg, 3.5 mmol) was added. Next, the reaction mixture was stirred for 4 h at room temperature, and the solvent was removed. The residue was purified by flash chromatography on silica gel (25% EtOAc/Hexane) to yield diketone **14** (893 mg, 89%) as a white solid.

#### Methyl (6a*R*,7*S*,­10a*S*)-3,7,10a-Tri­methyl-5,6,6a,7,­8,9,10,10a-octa­hydro­ben­zo­[*f*]­quin­oline-7-car­boxy­late (**23**)

To a solution of diketone **14** (370 mg, 1.20 mmol) in EtOH (15 mL), Cu­(OAc)_2_ (362 mg, 2.0 mmol) and NH_4_OAc (308 mg, 4.0 mmol) were added, and the reaction mixture was stirred at room temperature for 15 h. Following the same workup used in the general procedure and after column chromatography, using (30% EtOAc/Hexane), **23** was obtained (313 mg, 91%) as a white solid.

#### Synthesis of Forsyshiyanine A (**8**)

To a solution of **23** (170 mg, 0.59 mmol) in dry THF (3 mL) at 0 °C, LiAlH_4_ (94 mg, 2.47 mmol) was added, and the mixture was stirred at room temperature for 3 h. Then, 2 N HCl (5 mL) was carefully added, and the mixture was stirred for 5 min and extracted with Et_2_O (2 × 10 mL). Next, 2 N NaOH (10 mL) was added to the aqueous phase, and the mixture was extracted with EtOAc (2 × 20 mL). The organic phase was washed with H_2_O (10 mL) and brine (10 mL), dried over anhydrous Na_2_SO_4_, filtered, and the solvent was removed to give pure forsyshiyanine A (**8**) (145 mg, 95%) as a white solid. Its ^1^H and ^13^C NMR matched with those previously described.^8a^

#### Methyl (5*R*,6a*R*,­7*S*,10a*S*)-5-(Ben­zoyl­oxy)-3,7,10a-tri­methyl-5,6,6a,7,8,­9,10,10a-octa­hydro­ben­zo­[*f*]­quin­oline-7-carb­oxy­late (**24a**–**b**)

To a solution of **23** (73 mg, 0.254 mmol) in chlorobenzene (2 mL), NBS (53 mg, 0.3 mmol) and (BzO)_2_ (63 mg, 0.26 mmol) were added, and the mixture was heated at reflux for 3 h. Then, the solvent was removed under vacuum, and the residue was purified by flash chromatography on silica gel (40% EtOAc/Hexane) to give **24a**–**b** (66 mg, 64%) as a 7:3 mixture of epimers at C-7. White solid.

#### Methyl (6a*R*,7*S*,10a*S*)-3,7,10a-Tri­methyl-5-(*E*)-4-nitro­ben­zyli­dene)-5,6,6a,7,8,­9,10,10a-octa­hydro­ben­zo­[*f*]­quin­oline-7-carb­oxy­late (**25**)

To a solution of **23** (56 mg, 0.195 mmol) in AcOH (2 mL), *p*-nitrobenzaldehyde (30 mg, 0.2 mmol) and Ac_2_O (1 mL) were added, and the mixture was stirred at reflux for 2 h. Then, the mixture was diluted with H_2_O (5 mL), stirred for an additional 5 min, and extracted with EtOAc (2 × 10 mL). The combined organic layers were washed with saturated NaHCO_3_ (3 × 5 mL), H_2_O (2 × 5 mL), and brine (5 mL), dried over anhydrous Na_2_SO_4,_ filtered, and the solvent was removed. The residue was purified by flash chromatography on silica gel (40% EtOAc/hexane) to give **25** (56 mg, 68%) as a yellow solid.

#### Methyl (6a*R*,7*S*,10a*S*)-3,7,10a-Tri­methyl-5-oxo-5,6,6a,7,8,­9,10,10a-octa­hydro­ben­zo­[*f*]­quin­oline-7-carb­oxy­late (**26**)

To a solution of **23** (270 mg, 0.94 mmol) in acetone (10 mL) at 0 °C, H_2_O (2 mL) and KMnO_4_ (446 mg, 2.82 mmol) were added, and the mixture was stirred at room temperature for 15 h. Then, a 5% solution of NaHSO_3_ (3 mL) was added, and the reaction mixture was stirred for a further 5 min. Following the same workup used in the general procedure and after column chromatography, using (20% EtOAc/Hexane), ketone **26** was obtained (173 mg, 61%) as a colorless solid.

#### Methyl (7*S*,10a*S*)-3-Formyl-7,10a-di­methyl-5-oxo-5,7,8,­9,10,10a-hexa­hydro­ben­zo­[*f*]­quin­oline-7-carb­oxy­late (**27**)

To a solution of **23** (118 mg, 0.41 mmol) in chlorobenzene (3 mL), (PhSeO)_2_O (325 mg, 0.9 mmol) was added, and the mixture was stirred at reflux for 45 min. Then, the solvent was removed under vacuum, and the residue was purified by flash chromatography on silica gel (50% EtOAc/hexane) to afford **27** (117 mg, 91%) as a yellow syrup.

#### (7*S*,10a*S*)-7-(Meth­oxy­carb­on­yl)-7,10a-di­methyl-5-oxo-5,7,8,­9,10,10a-hexa­hydro­ben­zo­[*f*]­quin­oline-3-carb­oxy­lic Acid (**28**)

To a solution of **27** (47 mg, 0.15 mmol) in *t-*BuOH (2 mL), NaH_2_PO_4_ (60 mg, 0.5 mmol), H_2_O (2 mL), and 1-hexene (0.5 mL) were successively added. The mixture was cooled at 0 °C, and NaClO_2_ (70 mg, 0.62 mmol, 80% purity) was added. After stirring the reaction mixture for 15 h, 2N NaOH (2 mL) was added, and the solvent was removed. Next, the crude reaction was extracted with Et_2_O (2 × 10 mL), and the aqueous phase was acidified with 2 N HCl (3 mL). Then, the mixture was extracted with EtOAc (2 × 10 mL), the combined organic layers were washed with H_2_O (10 mL) and brine (10 mL), dried over anhydrous Na_2_SO_4_, filtered, and the solvent was removed. The residue was purified by flash chromatography on silica gel (60% EtOAc/hexane) to give **28** (43 mg, 87%) as a yellow solid.

#### Methyl (1*S*,4a*R*,­5*S*,8a*R*)-5-(3-(4-Meth­oxy­phen­yl)-3-oxo­prop­yl)-1,4a-di­methyl-6-meth­yl­ene­deca­hydro­naph­thal­ene-1-carb­oxy­late (**29**)

To a solution of nitrile **19** (620 mg, 2.14 mmol) in anhydrous THF (8 mL) at 0 °C, a solution of 4-methoxyphenylmagnesium chloride (0.5 M in THF, 5 mL, 2.5 mmol) was added, and the mixture was stirred at room temperature for 4 h. Then, the reaction was quenched with saturated NH_4_Cl (3 mL), and 2 N HCl (3 mL) was added. The new mixture was stirred for an additional 5 h. Next, the solvent was removed under vacuum, and the residue was extracted with EtOAc (2 × 20 mL). The combined organic layers were washed with H_2_O (15 mL) and brine (15 mL), dried over anhydrous Na_2_SO_4_, filtered, and the solvent was removed. The residue was purified by flash chromatography on silica gel (15% EtOAc/hexane) to give **29** (570 mg, 67%) as a colorless syrup.

#### Methyl (1*S*,4a*S*,­5*R*,8a*R*)-5-(3-(4-Meth­oxy­phen­yl)-3-oxo­prop­yl)-1,4a-di­methyl-6-oxo­deca­hydro­naph­thal­ene-1-carb­oxy­late (**30**)

A solution of ketone **29** (223 mg, 0.56 mmol) in EtOAc (15 mL) at −78 °C was bubbled with a constant stream of O_3_/O_2_ flow for 35 min. Then, the mixture was flushed with argon, and PPh_3_ (157 mg, 0.6 mmol) was added. Next, the mixture was stirred for 14 h at room temperature, and the solvent was removed. The residue was purified by flash chromatography on silica gel (20% EtOAc/hexane) to yield diketone **30** (181 mg, 81%) as a colorless syrup. This compound was immediately used in the next step.

#### Methyl (6a*R*,7*S*,10a*S*)-3-(4-Meth­oxy­phen­yl)-7,10a-di­methyl-5,6,6a,7,­8,9,10,10a-octa­hydro­ben­zo­[*f*]­quin­oline-7-carb­oxy­late (**31**)

To a solution of diketone **30** (108 mg, 0.27 mmol) in EtOH (5 mL), Cu­(OAc)_2_ (73 mg, 0.4 mmol) and NH_4_OAc (83 mg, 1.08 mmol) were added, and the mixture was stirred at room temperature for 12 h. Then, the mixture was filtered through a silica gel pad, and the solvent was removed. Following the same workup used in the general procedure and after column chromatography, using (30% EtOAc/hexane), **31** was obtained (95 mg, 93%) as a white solid.

### Biological Assays

Biological effects of compounds were evaluated using the MTT assay^15^ performed in a high-throughput 384-well-plate format according to MEDINA’s workflow as reported by Mackenzie et al.^16^ Compounds **8**, **23**–**28**, and **31** were tested at 50 μM per triplicate in A549 lung adenocarcinoma (CCL-185, ATCC), A2058 melanoma (CRL-3601, ATCC), HepG2 hepatocellular carcinoma (HB-8065, ATCC), MCF-7 breast cancer (HTB-22, ATCC) and Mia PaCa-2 pancreas carcinoma (CRL-1420, ATCC). Additionally, compounds **25** and **31** were tested at different concentrations (100, 50, 25, 12.5, 6.2, 3.1, 1.6, and 0.8 μM) per triplicate to obtain a dose–response curve in the same cell lines. Briefly, cells were seeded at 4.000 (A549, A2058 and Mia PaCa-2) or 8.000 cells/well (HepG2 and MCF-7) in a 384-well plate (Corning 384 Well TC-Treated Microplates). After 24 h, cells were treated with compounds for 72 h. MMS (methylmethanesulfonate, Sigma-Aldrich) 2 mM was used as positive control of cell death and DMSO (Dimethyl sulfoxide) 0.5% as negative control of cell death (same % as the compounds tested). Then, MTT dye (Thiazolyl blue tetrazolium bromide, ACROS Organics) 0.5 mg/mL was added for 3 h and absorbance was measured at 570 nm. Data obtained was analyzed using Genedata Screener Software.

## Supplementary Material





## Notes

Characterization data for compounds **18**, **19**, **23**–**29** and **31**; copies of ^1^H and ^13^C NMR, UV, IR and HRMS spectra of compounds **18**, **19**, **23**–**29** and **31** (PDF)
NMR raw data files for compounds **18**, **19**, **23**–**29** and **31** (ZIP)
