# Supplementary material for: Semisynthesis of Forsyshiyanine A and a Derivative with Significant Anti-Pancreatic Cancer Activity
Source: J Nat Prod. 2025 Nov 3;88(11):2781–8. doi: 10.1021/acs.jnatprod.5c01178 (PMC12670700; doi:10.1021/acs.jnatprod.5c01178)
Supplement: Supplementary file 1 [file np5c01178_si_001.pdf]

Supporting Information

Semisynthesis of Forsyshiyanine A and a Derivative with Significant Anti-Pancreatic Cancer Activity

Saad Y. Rfaish†, Antonio Fernández†, María C. Ramos¥, Thomas A Mackenzie¥, José Justicia†*, Rachid Chahboun†*

†Departamento de Química Orgánica, Facultad de Ciencias, Instituto de Biotecnología, Universidad de Granada, 18071 Granada, Spain

¥Fundación MEDINA, Av. Conocimiento 34, Health Sciences Technology Park, 18016 Granada, Spain

E-mail: jjusti@ugr.es, rachid@ugr.es

Contents

- Characterization Data of the Synthesized Compounds S3-S8
  - Data for **18** S3
  - Data for **19** S3
  - Data for **23** S4
  - Data for **24** S4
  - Data for **25** S5
  - Data for **26** S5
  - Data for **27** S6
  - Data for **28** S6
  - Data for **29** S7
  - Data for **31** S7
- ^{1}H, ^{13}C NMR, UV, IR and HRMS spectra for new compounds S9-S48
  - **Figure S1.** ^{1}H NMR spectrum of **8** S9
  - **Figure S2.** ^{1}H NMR spectrum of **18** S10
  - **Figure S3.** ^{13}C NMR spectrum of **18** S11
  - **Figure S4.** UV and IR spectra report for **18** S12

Figure S5. ^{1}H NMR spectrum of **19** S13
Figure S6. ^{13}C NMR spectrum of **19** S14
Figure S7. UV, IR spectra report for **19** S15
Figure S8. HRMS report for **19** S16
Figure S9. ^{1}H NMR spectrum of **23** S17
Figure S10. ^{13}C NMR spectrum of **23** S18
Figure S11. UV and IR spectra report for **23** S19
Figure S12. HRMS report for **23** S20
Figure S13. ^{1}H NMR spectrum of **24** S21
Figure S14. ^{13}C NMR spectrum of **24** S22
Figure S15. UV and IR spectra report for **24** S23
Figure S16. HRMS report for **24** S24
Figure S17. ^{1}H NMR spectrum of **25** S25
Figure S18. ^{13}C NMR spectrum of **25** S26
Figure S19. UV and IR spectra report for **25** S27
Figure S20. HRMS report for **25** S28
Figure S21. ^{1}H NMR spectrum of **26** S29
Figure S22. ^{13}C NMR spectrum of **26** S30
Figure S23. UV and IR spectra report for **26** S31
Figure S24. HRMS report for **26** S32
Figure S25. ^{1}H NMR spectrum of **27** S33
Figure S26. ^{13}C NMR spectrum of **27** S34
Figure S27. UV and IR spectra report for **27** S35
Figure S28. HRMS report for **27** S36
Figure S29. ^{1}H NMR spectrum of **28** S37
Figure S30. ^{13}C NMR spectrum of **28** S38
Figure S31. UV and IR spectra report for **28** S39
Figure S32. HRMS report for **28** S40
Figure S33. ^{1}H NMR spectrum of **29** S41
Figure S34. ^{13}C NMR spectrum of **29** S42
Figure S35. UV and IR spectra report for **29** S43
Figure S36. HRMS report for **29** S44
Figure S37. ^{1}H NMR spectrum of **31** S45
Figure S38. ^{13}C NMR spectrum of **31** S46
Figure S39. UV and IR spectra report for **31** S47
Figure S40. HRMS report for **31** S48

Characterization Data of the Synthesized Compounds

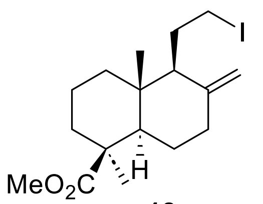

18

*Methyl (1S,4aR,5S,8aR)-5-(2-iodoethyl)-1,4a-dimethyl-6-methylenedecahydronaphthalene-1-carboxylate (**18**).* $[\alpha]_D^{25}$ + 58.3 (c 3.61, $CHCl_3$); UV (MeCN) λ_{max} (log ε) 254 (2.69) nm; IR (thin film, ν cm^{-1}): 2941, 2845, 1723, 1445, 1221, 1157, 889; ^1H NMR (400 MHz, $CDCl_3$) δ 4.83 (bs, 1H), 4.44 (br s, 1H), 3.59 (s, 3H), 3.32 (ddd, J = 9.4, 8.3, 4.1 Hz, 1H), 3.04 (td, J = 9.1, 7.6 Hz, 1H), 2.39 (ddd, J = 12.4, 4.0, 2.5 Hz, 1H), 2.15 (dtd, J = 13.3, 3.3, 1.5 Hz, 1H), 2.07-1.99 (m, 1H), 1.98–1.92 (m, 1H), 1.91–1.84 (m, 2H), 1.83–1.78 (m, 1H), 1.77–1.71 (m, 3H), 1.56–1.47 (m, 1H), 1.32 (dd, J = 12.6, 3.0 Hz, 1H), 1.17 (s, 3H), 1.11 (td, J = 13.2, 4.3 Hz, 1H), 1.04 (td, J = 13.4, 4.1 Hz, 1H), 0.48 (s, 3H); ^{13}C NMR (100 MHz, $CDCl_3$) δ 177.6 (C), 147.1 (C), 106.3 (CH_2), 56.8 (CH), 56.1 (CH), 51.3 (CH_3), 44.3 (C), 40.3 (C), 39.0 (CH_2), 38.6 (CH_2), 38.2 (CH_2), 29.0 (CH_2), 28.9 (CH_3), 26.2 (CH_2), 19.9 (CH_2), 13.0 (CH_3), 7.0 (CH_2); A good HRMS could not be obtained.

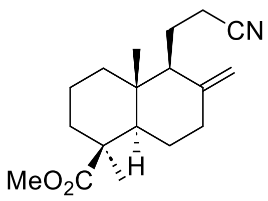

19

*Methyl (1S,4aR,5S,8aR)-5-(2-cyanoethyl)-1,4a-dimethyl-6-methylenedecahydronaphthalene -1-carboxylate (**19**).* $[\alpha]_D^{25}$ + 61.71 (c 3.36, $CHCl_3$); UV (MeCN) λ_{max} (log ε) 253 (2.70) nm; IR (thin film, ν cm^{-1}): 2951, 2336, 2246, 1714, 1441, 1215, 1154; ^1H NMR (400 MHz, $CDCl_3$) δ 4.88 (br s, 1H), 4.40 (br s, 1H), 3.59 (s, 3H), 2.49–2.43 (m, 1H), 2.42–2.37 (m, 1H), 2.29–2.21 (m, 1H), 2.21–2.13 (m, 2H), 2.03–1.96 (m, 1H), 1.95–1.86 (m, 2H), 1.86–1.71 (m, 3H), 1.71–1.63 (m, 1H), 1.53 (dt, J = 14.4, 3.6 Hz, 1H), 1.32 (dd, J = 12.5, 3.0 Hz, 1H), 1.17 (s, 3H), 1.14 (td, J = 13.2, 4.3 Hz, 1H), 1.04 (td, J = 13.5, 4.1 Hz, 1H), 0.49 (s, 3H); ^{13}C NMR (100 MHz, $CDCl_3$) δ 177.6 (C), 146.8 (C), 120.2 (C), 106.5 (CH_2), 56.1 (CH), 55.1 (CH), 51.3 (CH_3), 44.3 (C), 40.3 (C), 39.1 (CH_2), 38.5 (CH_2), 38.1 (CH_2), 28.9 (CH_3), 26.2 (CH_2), 20.4 (CH_2), 19.9 (CH_2), 16.3 (CH_2), 12.7 (CH_3); HRMS (ESI) *m/z* calcd for $C_{18}H_{27}NO_2Na$ $(M+Na^+)$ 312.1936, found 312.1939.

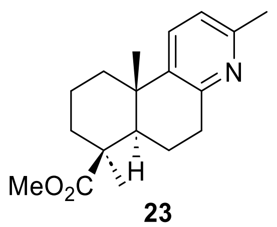

23

*Methyl (6aR,7S,10aS)-3,7,10a-trimethyl-5,6,6a,7,8,9,10,10a-octahydrobenzo[f]quinoline-7-carboxylate (**23**)*. $[\alpha]_D^{25}$ + 124.56 (c 1.46, $CHCl_3$); UV (MeCN) λ_{max} (log ε) 274 (3.82) nm; IR (thin film, ν cm^{-1}): 2940, 2892, 1715, 1592, 1463, 1431, 1375, 1234, 1147, 1034, 826; ^1H NMR (400 MHz, $CDCl_3$) δ 7.34 (d, *J* = 8.1 Hz, 1H), 6.81 (d, *J* = 8.1 Hz, 1H), 3.54 (s, 3H), 2.95 (dd, *J* = 17.7, 5.4 Hz, 1H), 2.76 (m, 1H), 2.35 (s, 3H), 2.20–2.11 (m, 2H), 2.06 (d, *J* = 12.9 Hz, 1H), 2.01–1.80 (m, 2H), 1.50 (dt, *J* = 14.1, 3.6 Hz, 1H), 1.40 (dd, *J* = 12.4, 1.9 Hz, 1H), 1.21 (td, *J* = 13.3, 4.3 Hz, 1H), 1.16 (s, 3H), 0.95 (td, *J* = 13.5, 4.3 Hz, 1H), 0.88 (s, 3H); ^{13}C NMR (100 MHz, $CDCl_3$) δ 177.4 (C), 154.6 (C), 154.4 (C), 139.9 (C), 134.0 (CH), 120.8 (CH), 51.9 (CH), 51.1 (CH_3), 43.7 (C), 38.9 (CH_2), 37.5 (CH_2), 37.4 (C), 34.4 (CH_2), 28.2 (CH_3), 23.5 (CH_3), 22.7 (CH_3), 20.6 (CH_2), 19.7 (CH_2); HRMS (ESI) *m/z* calcd for $C_{18}H_{26}NO_2$ ($M+H^+$) 288.1958, found 288.1957.

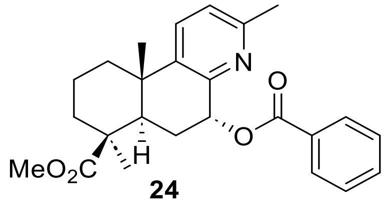

24

*Methyl (5R,6aR,7S,10aS)-5-(benzoyloxy)-3,7,10a-trimethyl-5,6,6a,7,8,9,10,10a-octahydrobenzo[f]quinoline-7-carboxylate (**24**)*. 7:3 mixture of epimers at C-7. $[\alpha]_D^{25}$ + 1.96 (c 0.346, $CHCl_3$); UV (MeCN) λ_{max} (log ε) 279 (3.67) nm; IR (thin film, ν cm^{-1}): 1731, 1447, 1367, 1219, 773; ^1H NMR (400 MHz, $CDCl_3$) δ 8.09 (d, *J* = 7.9 Hz, 2H), 7.76 (d, *J* = 8.3 Hz, 1H), 7.58 (t, *J* = 7.0 Hz, 1H), 7.45 (t, *J* = 7.7 Hz, 2H), 7.24 (d, *J* = 8.1 Hz, 1H), 6.02 (br s, 1H), 5.76 (br s, 1H, minor isomer), 3.65 (s, 3H), 2.71 (br s, 3H), 2.65–2.50 (m, 2H), 2.34 (d, *J* = 13.3 Hz, 1H), 2.26 (dd, *J* = 11.9, 1.9 Hz, 1H), 2.23–2.14 (m, 1H), 2.05–1.90 (m, 1H), 1.74–1.64 (m, 1H), 1.46 (td, *J* = 13.3, 3.9 Hz, 1H), 1.29 (s, 3H), 1.25–1.10 (m, 1H), 0.97 (s, 3H, minor isomer), 0.96 (s, 3H); ^{13}C NMR (100 MHz, $CDCl_3$) δ 177.4 (C, minor isomer), 177.3 (C), 170.7 (C), 155.3 (C, minor isomer), 155.1 (C), 150.9 (C), 150.2 (C, minor isomer), 141.7 (C, minor isomer), 141.5 (C), 138.5 (CH),

138.3 (CH, minor isomer), 133.5 (CH), 133.4 (CH, minor isomer), 130.2 (CH), 130.0 (CH, minor isomer), 128.5 (CH), 125.53 (CH, minor isomer), 125.50 (CH), 77.4 (CH, minor isomer), 51.7 (CH_3), 50.0 (CH_3, minor isomer), 46.2 (CH), 45.3 (CH, minor isomer), 43.4 (C), 43.39 (C, minor isomer), 38.3 (CH_2, minor isomer), 38.2 (CH_2), 37.1 (CH_2, minor isomer), 37.0 (CH_2), 30.6 (CH_2), 30.1 (CH_2, minor isomer), 27.9 (CH_3, minor isomer), 27.8 (CH_3), 23.0 (CH_3), 22.4 (CH_3, minor isomer), 22.1 (CH_3, minor isomer), 22.0 (CH_3), 19.6 (CH_2); HRMS (ESI) *m/z* calcd for $C_{25}H_{30}NO_4$ ($M+H^+$) 408.2169, found 408.2168.

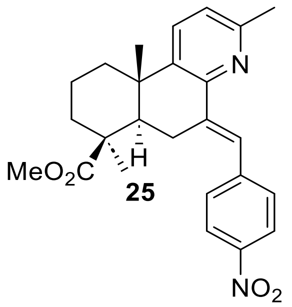

25

*Methyl (6aR,7S,10aS)-3,7,10a-trimethyl-5-(E)-4-nitrobenzylidene)-5,6,6a,7,8,9,10,10a-octahydrobenzo[f]quinoline-7-carboxylate (**25**).* $[\alpha]_D^{25}$ + 194.01 (c 0.117, $CHCl_3$); UV (MeCN) λ_{max} (log ε) 360 (3.66) nm; IR (thin film, ν cm^{-1}): 1738, 1437, 1365, 1228, 1216; ^1H NMR (400 MHz, $CDCl_3$) δ 8.25 (d, J = 8.6 Hz, 2H), 8.02 (br s, 1H), 7.66 (d, J = 8.6 Hz, 2H), 7.59 (d, J = 7.6 Hz, 1H), 7.09 (d, J = 7.6 Hz, 1H), 3.72 (s, 3H), 3.34 (dd, J = 16.4, 2.8 Hz, 1H), 3.12 (t, J = 14.8 Hz, 1H), 2.62 (bs, 3H), 2.33–2.19 (m, 2H), 1.99 (m, 1H), 1.72–1.62 (m, 2H), 1.47–1.23 (m, 3H), 1.18 (s, 3H), 1.08 (s, 3H); ^{13}C NMR (100 MHz, $CDCl_3$) δ 190.4 (C), 177.2 (C), 155.6 (C), 148.9 (C), 146.3 (C), 144.8 (C), 141.3 (C), 130.6 (CH), 130.4 (2 x CH), 124.4 (CH), 123.7 (2 x CH), 123.5 (CH), 51.8 (CH_3), 50.7 (CH), 44.0 (C), 38.8 (CH_2), 37.9 (C), 37.5 (CH_2), 28.4 (CH_3), 26.1 (CH_2), 21.8 (2 x CH_3), 19.9 (CH_2); HRMS (ESI) *m/z* calcd for $C_{25}H_{28}N_2O_4$ (M^+) 420.2044, found 420.2031.

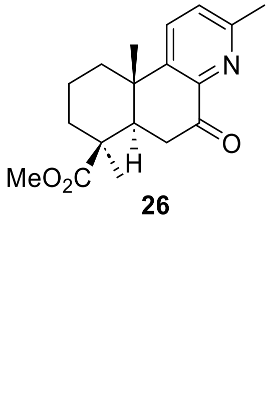

26

*Methyl (6aR,7S,10aS)-3,7,10a-trimethyl-5-oxo-5,6,6a,7,8,9,10,10a-octahydrobenzo[f]quinoline-7-carboxylate (**26**)*. $[\alpha]_D^{25}$ + 75.41 (c 1.2, $CHCl_3$); UV (MeCN) λ_{max} (log ε) 289 (2.25) nm; IR (thin film, ν cm^{-1}): 1724, 1702, 1467, 1375, 1216, 1141; ^{1}H NMR (400 MHz, $CDCl_3$) δ 7.69 (d, *J* = 8.2 Hz, 1H), 7.29 (d, *J* = 8.2 Hz, 1H), 3.67 (s, 3H), 3.39–3.00 (m, 2H), 2.60 (s, 3H), 2.28 (dq, *J* = 12.3, 3.3 Hz, 2H), 2.15–1.91 (m, 3H), 1.68 (dt, *J* = 14.4, 3.6 Hz, 1H), 1.43 (td, *J* = 13.4, 4.3 Hz, 1H), 1.23 (s, 3H), 1.09 (s, 3H); ^{13}C NMR (100 MHz, $CDCl_3$) δ 197.3 (C), 176.8 (C), 157.6 (C), 147.8 (C), 145.2 (C), 134.5 (CH), 127.9 (CH), 51.73 (CH_3), 49.7 (CH), 43.8 (C), 38.5 (C), 38.3 (CH_2), 38.2 (C), 37.3 (CH_2), 27.9 (CH_3), 24.2 (CH_3), 21.2 (CH_3), 19.5 (CH_2); HRMS (ESI) *m/z* calcd for $C_{18}H_{24}NO_3$ (M+H$^+$) 302.1751, found 302.1744.

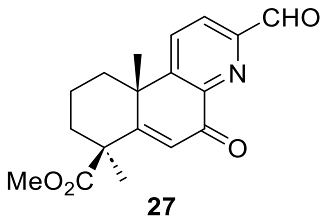

27

*Methyl (7S,10aS)-3-formyl-7,10a-dimethyl-5-oxo-5,7,8,9,10,10a-hexahydrobenzo[f]quinoline-7-carboxylate (**27**)*. $[\alpha]_D^{25}$ + 75.41 (c 1.2, $CHCl_3$); UV (MeCN) λ_{max} (log ε) 249 (3.65) nm; IR (thin film, ν cm^{-1}): 2950, 1723, 1669, 1459, 1240, 1144, 767; ^{1}H NMR (400 MHz, $CDCl_3$) δ 10.26 (s, 1H), 8.18–8.09 (m, 2H), 6.82 (s, 1H), 3.66 (s, 3H), 2.63–2.52 (m, 1H), 2.38 (bd, *J* = 13.1 Hz, 1H), 2.18 (dt, *J* = 13.9, 3.8 Hz, 1H), 1.83–1.74 (m, 1H), 1.57 (td, *J* = 13.5, 4.3 Hz, 1H), 1.52 (s, 3H), 1.38 (s, 3H), 1.26 (td, *J* = 13.7, 4.5 Hz, 1H); ^{13}C NMR (100 MHz, $CDCl_3$) δ 193.1 (CH), 183.2 (C), 174.9 (C), 165.2 (C), 152.1 (C), 152.0 (C), 145.8 (C), 136.4 (CH), 127.6 (CH), 123.8 (CH), 52.5 (CH_3), 48.3 (C), 43.1 (C), 39.7 (CH_2), 36.9 (CH_2), 27.3 (CH_3), 27.2 (CH_3), 19.0 (CH_2); HRMS (ESI) *m/z* calcd for $C_{18}H_{19}NO_4Na$ (M+Na$^+$) 336.1212, found 336.1217.

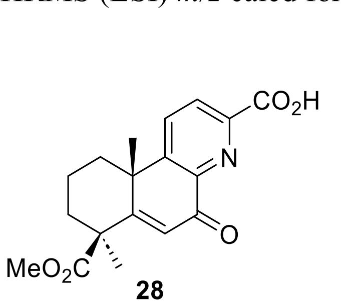

28

*(7S,10aS)-7-(methoxycarbonyl)-7,10a-dimethyl-5-oxo-5,7,8,9,10,10a-hexahydrobenzo[f]quinoline-3-carboxylic acid (**28**)*. $[\alpha]_D^{25}$ + 215.6 (c 0.353, $CHCl_3$); UV

(MeCN) λ_{max} (log ε) 270 (3.69) nm; IR (thin film, ν cm^{-1}): 2933, 2861, 1729, 1662, 1459, 1249, 1148; ^1H NMR (400 MHz, $CDCl_3$) δ 8.40 (d, *J* = 8.3 Hz, 1H), 8.18 (d, *J* = 8.3 Hz, 1H), 6.79 (s, 1H), 3.67 (s, 3H), 2.59 (dt, *J* = 13.8, 1.9 Hz, 1H), 2.45–2.34 (m, 1H), 2.19 (qt, *J* = 13.8, 3.7 Hz, 1H), 1.80 (dq, *J* = 14.1, 3.1 Hz, 1H), 1.53 (s, 3H), 1.39 (s, 3H), 1.37–1.19 (m, 2H); ^{13}C NMR (100 MHz, $CDCl_3$) δ 183.1 (C), 174.7 (C), 166.6 (C), 164.4 (C), 151.6 (C), 146.4 (C), 143.9 (C), 137.5 (CH), 127.2 (CH), 126.9 (CH), 52.5 (CH_3), 48.4 (C), 43.1 (C), 39.7 (CH_2), 36.9 (CH_2), 27.2 (CH_3), 19.0 (CH_2); HRMS (ESI) *m/z* calcd for $C_{18}H_{19}NO_5$ ($M+Na^+$) 352.1161, found 352.1160.

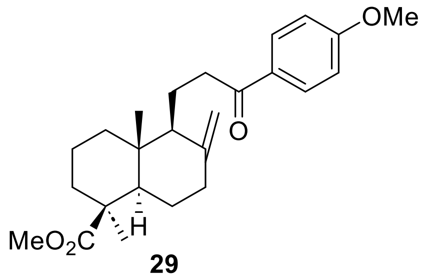

29

*Methyl (1S,4aR,5S,8aR)-5-(3-(4-methoxyphenyl)-3-oxopropyl)-1,4a-dimethyl-6-methylenedecahydronaphthalene-1-carboxylate (**29**).* $[\alpha]_D^{25}$ + 41.29 (c 1.16, $CHCl_3$); UV (MeCN) λ_{max} (log ε) 271 (3.75) nm; IR (thin film, ν cm^{-1}): 2939, 2847, 1722, 1675, 1600, 1454, 1253, 1164; ^1H NMR (400 MHz, $CDCl_3$) δ 7.91 (d, *J* = 8.5 Hz, 2H), 6.90 (d, *J* = 8.5 Hz, 2H), 4.88 (s, 1H), 4.53 (s, 1H), 3.85 (s, 3H), 3.60 (s, 3H), 3.09–2.97 (m, 1H), 2.88–2.76 (m, 1H), 2.45–2.37 (m, 1H), 2.16 (t, *J* = 6.9 Hz, 1H), 2.08–1.76 (m, 7H), 1.35–1.17 (m, 2H), 1.16 (s, 3H), 1.04 (td, *J* = 13.3, 4.0 Hz, 1H), 0.54 (s, 3H); ^{13}C NMR: (100 MHz, $CDCl_3$) δ 199.5 (C), 177.8 (C), 163.4 (C), 148.1 (C), 130.4 (C), 130.3 (CH), 113.7 (CH), 106.6 (CH_2), 56.4 (CH), 55.8 (CH), 55.5 (CH_3), 51.2 (CH_3), 44.4 (C), 40.6 (C), 39.2 (CH_2), 38.8 (CH_2), 38.3 (CH_2), 37.3 (CH_2), 29.0 (CH_3), 26.4 (CH_2), 20.1 (CH_2), 18.5 (CH_2), 12.6 (CH_3); HRMS (ESI) *m/z* calcd for $C_{25}H_{34}O_4Na$ ($M+Na^+$) 421.2355, found 421.2362.

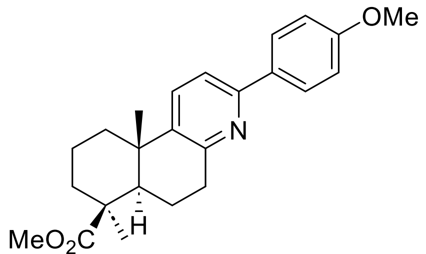

31

*Methyl (6aR,7S,10aS)-3-(4-methoxyphenyl)-7,10a-dimethyl-5,6,6a,7,8,9,10,10a-octahydrobenzo[f]quinoline-7-carboxylate (**31**)*. $[\alpha]_D^{25}$ + 83.47 (c 0.53, $CHCl_3$); UV (MeCN) λ_{max} (log ε) 265 (3.70), 345 (2.68) nm; IR (thin film, ν cm^{-1}): 2943, 2205, 1722, 1598, 1454, 1245, 1169, 1029, 769; ^1H NMR (500 MHz, $CDCl_3$) δ 8.10 (d, *J* = 8.3 Hz, 2H), 8.03 (d, *J* = 8.3 Hz, 1H), 7.65 (d, *J* = 8.5 Hz, 1H), 7.07 (d, *J* = 8.3 Hz, 2H), 3.87 (s, 3H), 3.69 (s, 3H), 2.40 (dd, *J* = 14.4, 6.3 Hz, 1H), 2.33 (dd, *J* = 14.0, 3.2 Hz, 1H), 2.25 (d, *J* = 12.7 Hz, 1H), 2.08 (dd, *J* = 14.0, 4.4 Hz, 1H), 2.06–2.00 (m, 2H), 1.70 (d, *J* = 14.3 Hz, 2H), 1.55 (dd, *J* = 12.5, 1.8 Hz, 1H), 1.40 (ddd, *J* = 13.1, 13.2, 4.1 Hz, 1H), 1.30 (s, 3H), 1.12 (ddd, *J* = 13.7, 13.7, 4.2 Hz, 1H), 1.09 (s, 3H); ^{13}C NMR (125 MHz, $CDCl_3$) δ 177.1 (C), 163.2 (C), 153.1 (C), 151.3 (C), 144.7 (C), 142.1 (CH), 131.1 (2 x CH), 122.1 (CH), 115.1 (2 x CH), 55.7 (CH_3), 51.7 (CH_3), 50.9 (CH), 43.9 (C), 38.8 (CH_2), 38.4 (C), 37.3 (CH_2), 31.1 (CH), 30.6 (CH_2), 28.3 (CH_3), 22.8 (CH_3), 19.6 (CH_2), 19.3 (CH_2); HRMS (ESI) *m/z* calcd for $C_{24}H_{30}NO_3$ ($M+H^+$) 380.2226, found 380.2225.

^{1}H, ^{13}C NMR, UV, IR and HRMS Spectra of New Compounds

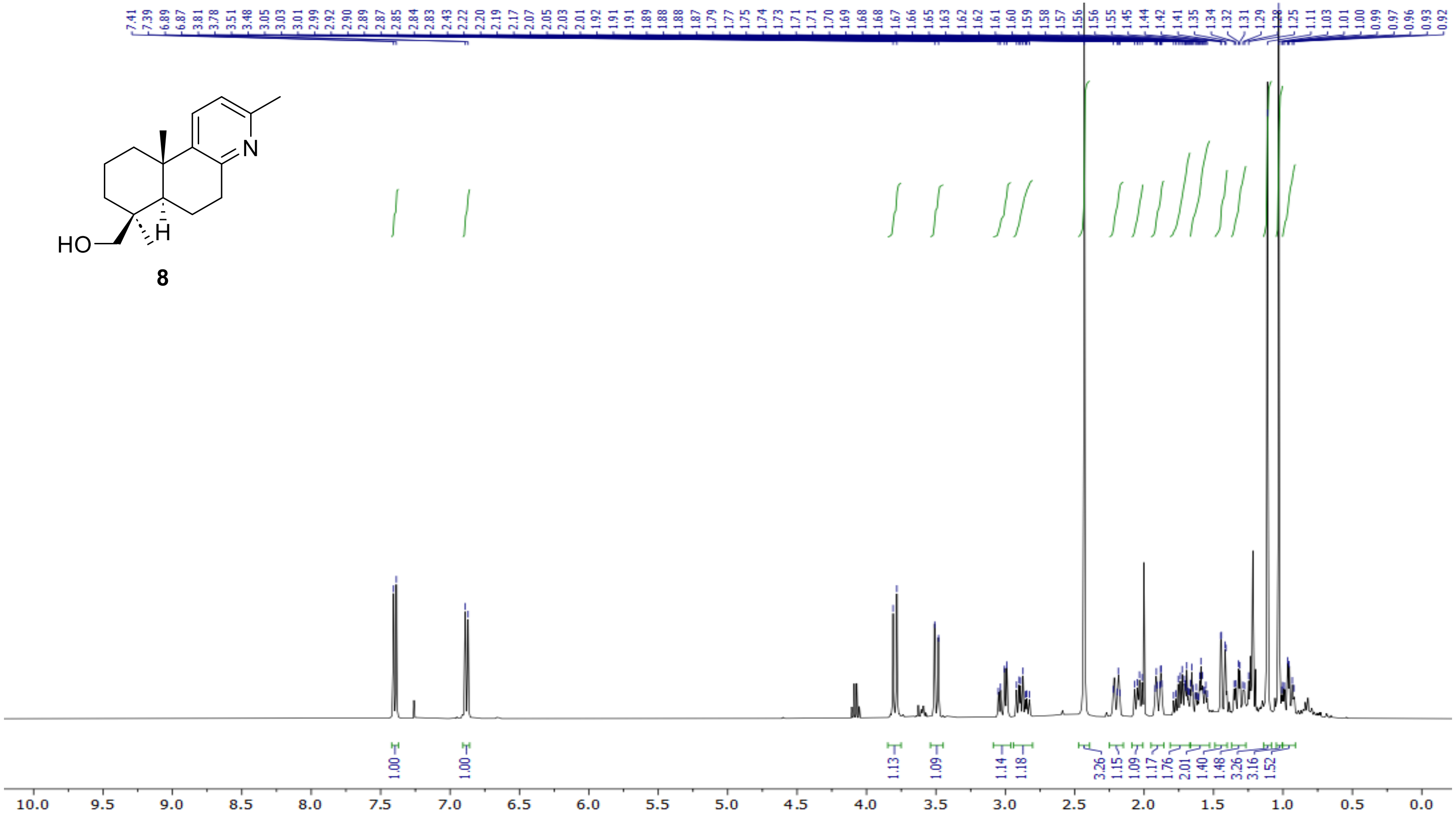

Figure S1. ^{1}H NMR spectrum of **8** ($CDCl_3$, 400 MHz).

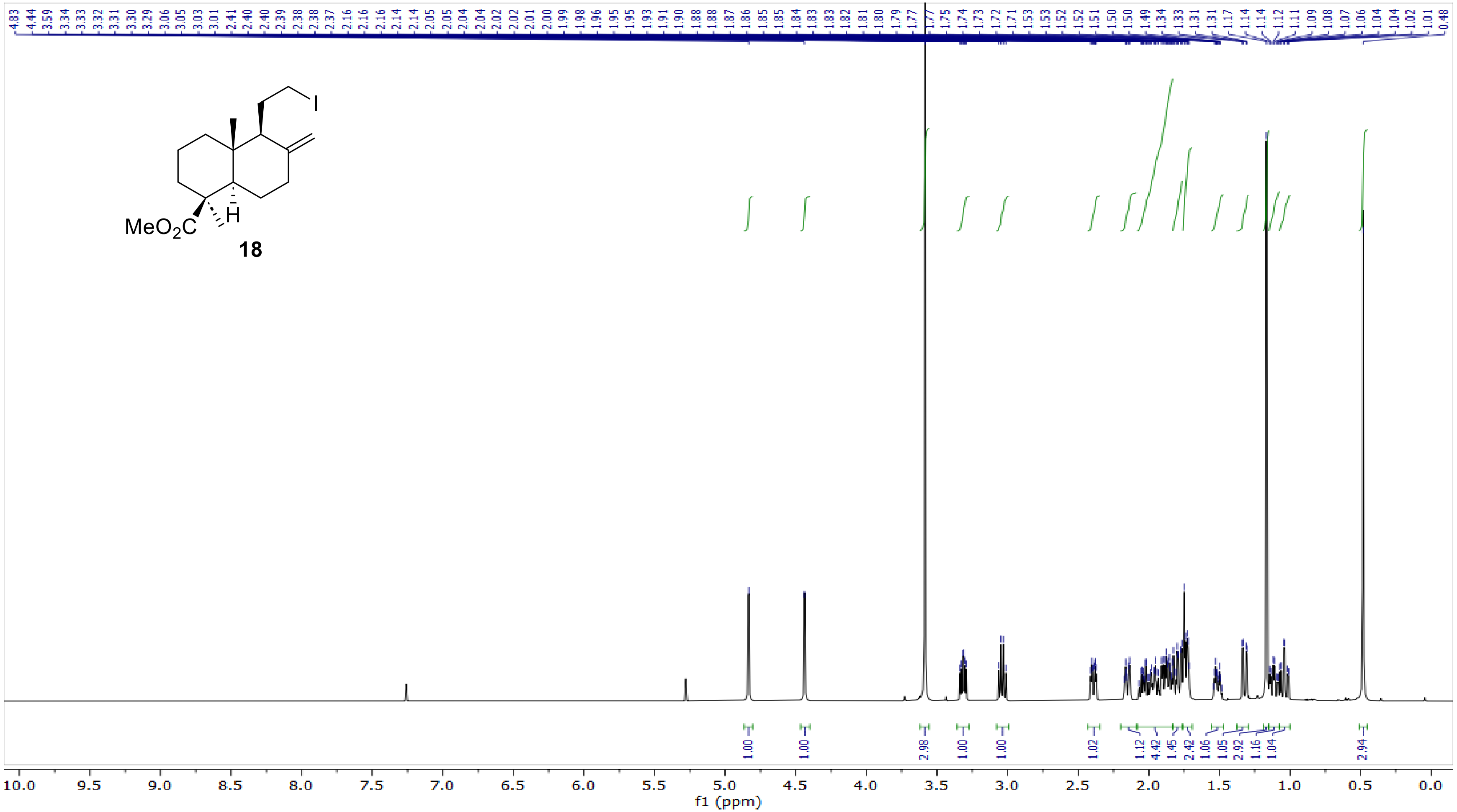

Figure S2. ^{1}H NMR spectrum of **18** ($CDCl_3$, 400 MHz).

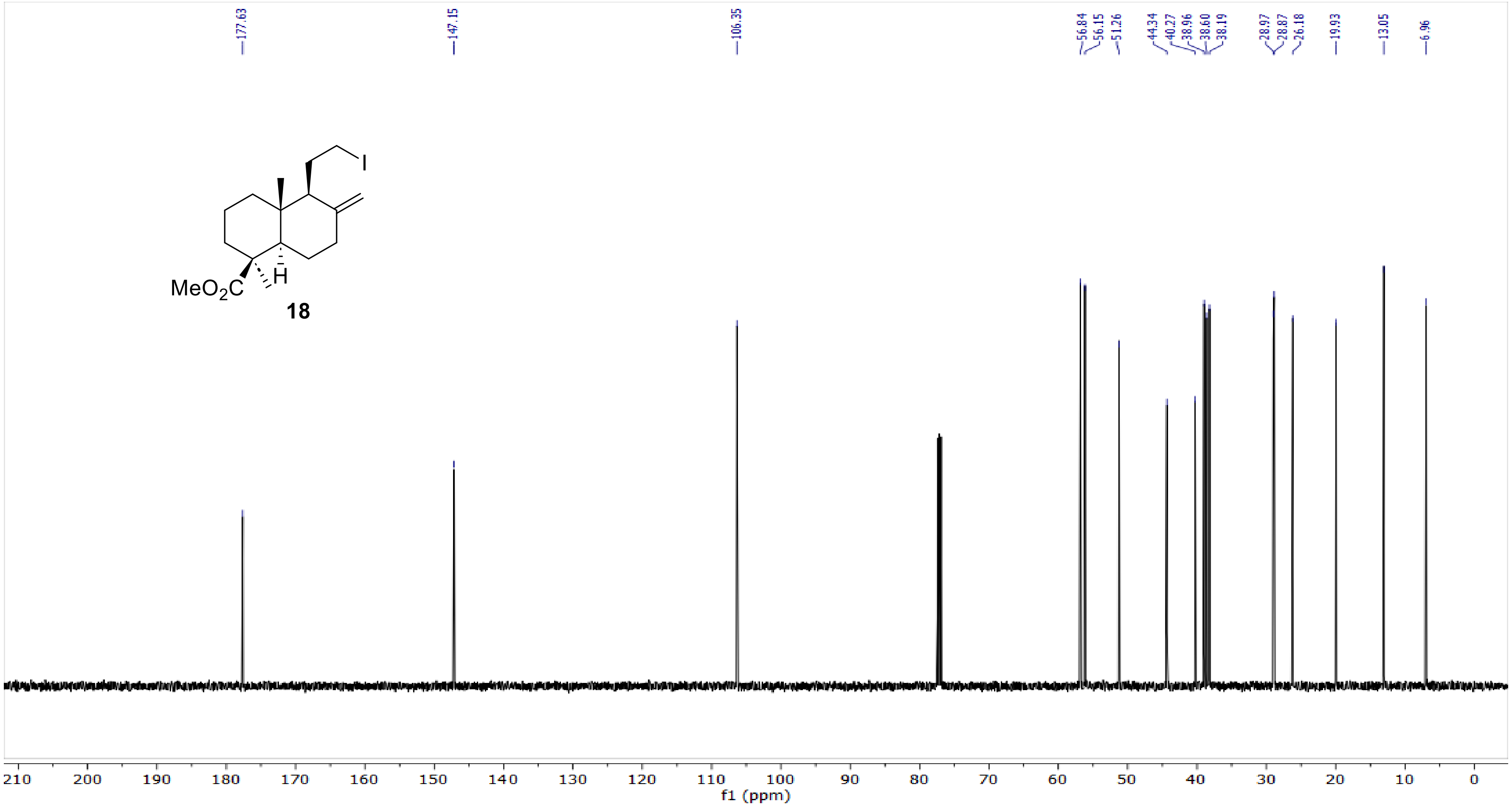

Figure S3. ^{13}C NMR spectrum of **18** ($CDCl_3$, 100 MHz).

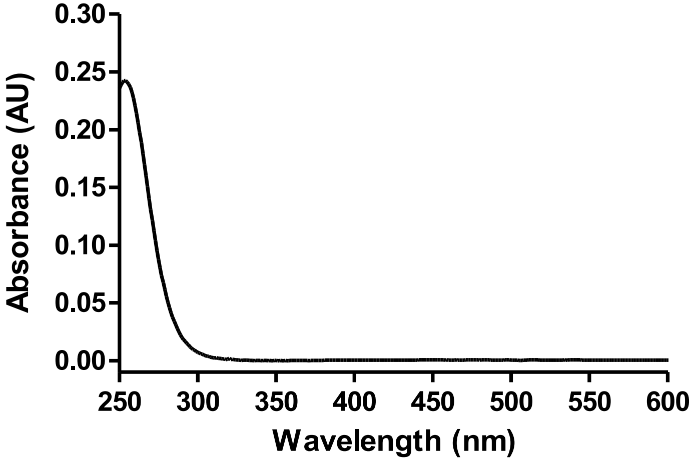

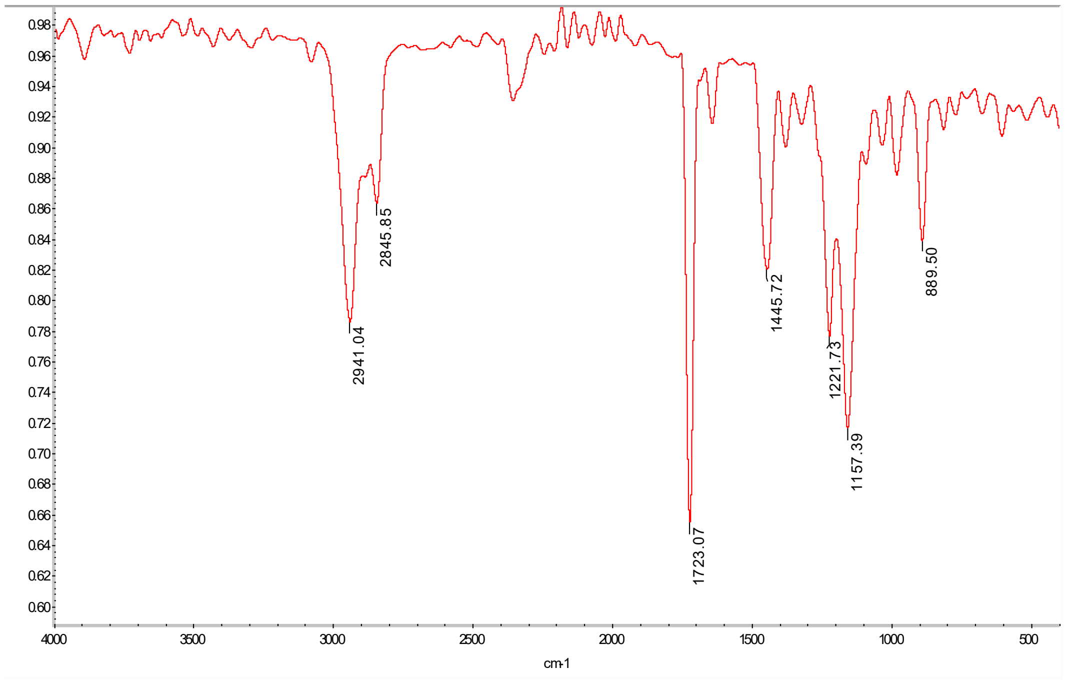

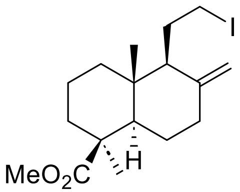

18

Figure S4. UV (in MeCN) and IR (thin film) spectra report for **18**.

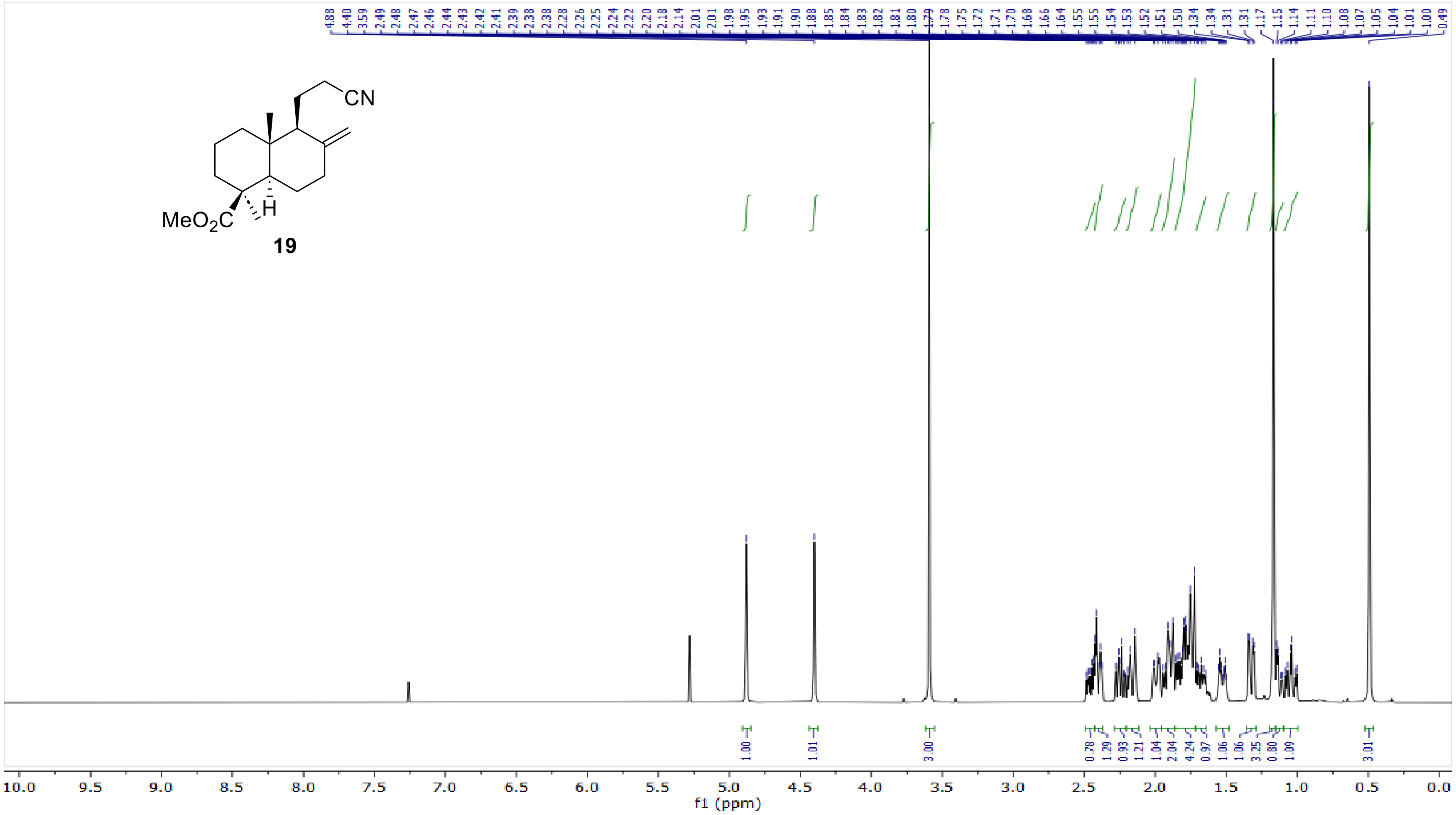

Figure S5. ^{1}H NMR spectrum of **19** ($CDCl_3$, 400 MHz).

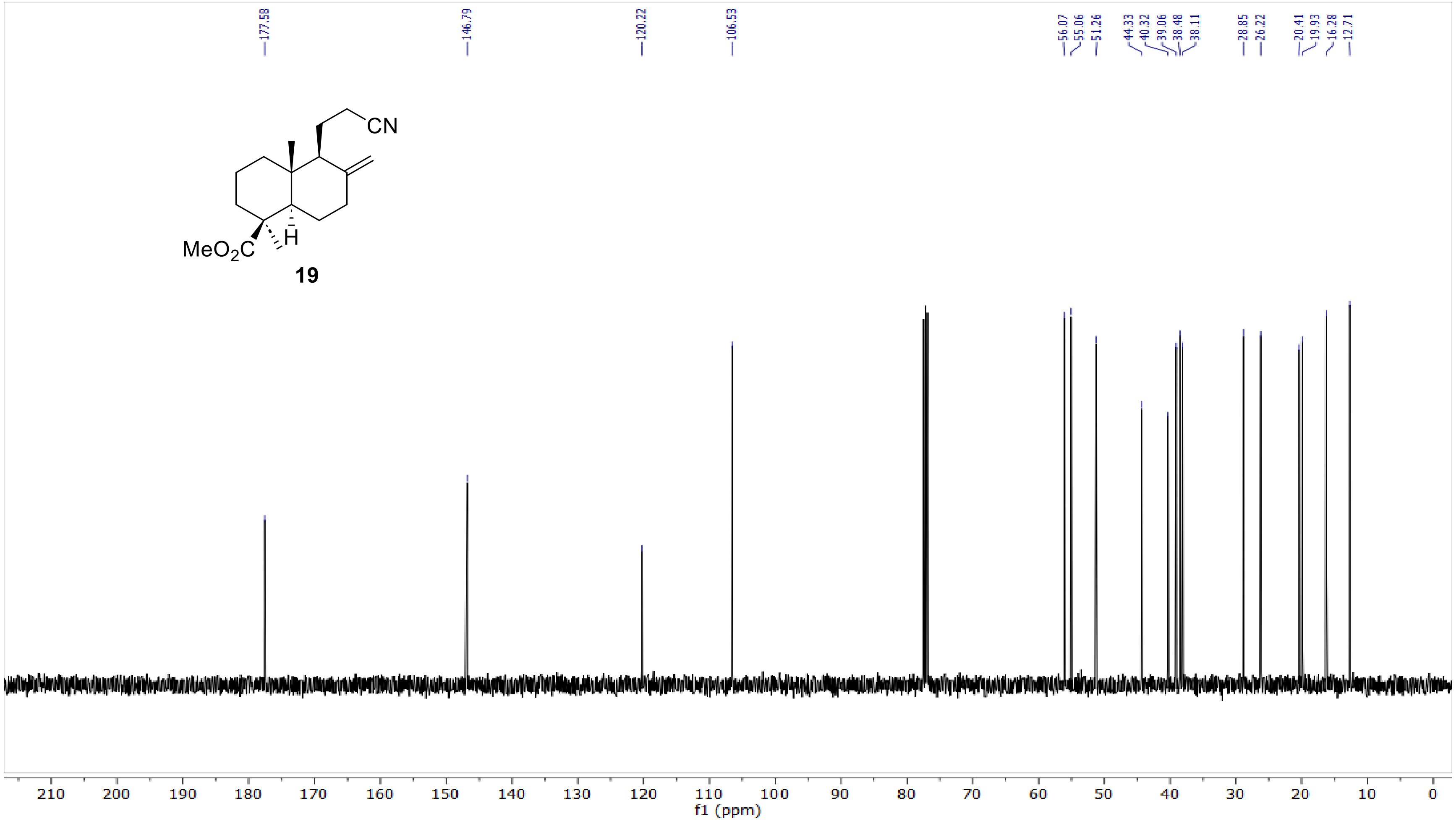

Figure S6. ^{13}C NMR spectrum of **19** ($CDCl_3$, 100 MHz).

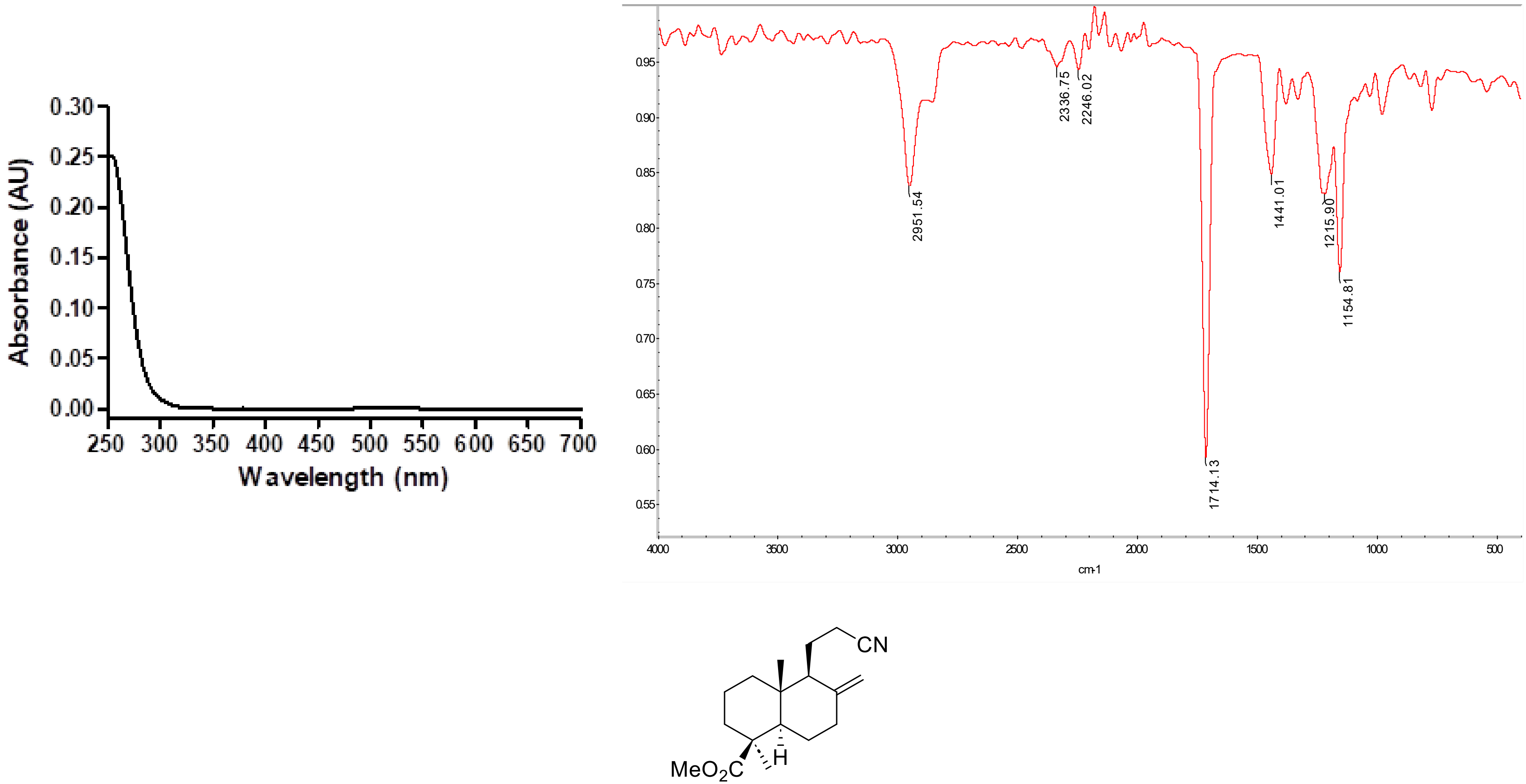

19

Figure S7. UV (in MeCN) and IR (KBr pellet) spectra report for **19**.

Elemental Composition Report

Page 1

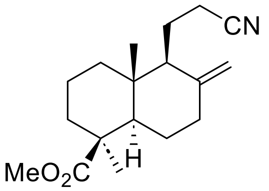

19

Multiple Mass Analysis: 31 mass(es) processed

Tolerance = 5.0 PPM / DBE: min = -400.0, max = 400.0
Element prediction: Off
Number of isotope peaks used for i-FIT = 3

Monoisotopic Mass, Even Electron Ions
499 formula(e) evaluated with 7 results within limits (all results (up to 1000) for each mass)
Elements Used:
C: 18-18 H: 0-50 N: 0-1 O: 0-5 Na: 0-1

JJRac11-bis 6.00000000
JJRac11-bis 49 (1.855) Cm (21:90) 1: TOF MS ES+ 1.07e+008

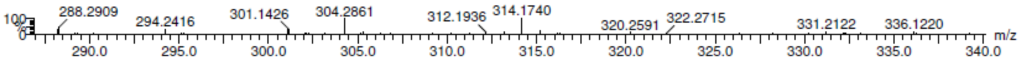

Minimum:	5.00				-400.0				
Maximum:	100.00		3.0	5.0	400.0				

Mass	RA	Calc. Mass	mDa	PPM	DBE	i-FIT	Norm	Conf(%)	Formula
288.1971	29.39	288.1964	0.7	2.4	6.5	2636.4	n/a	n/a	C18 H26 N O2
288.2909	43.90	---							
289.1996	6.48	---							
289.2943	9.06	---							
290.2112	9.37	290.2120	-0.8	-2.8	5.5	2749.9	n/a	n/a	C18 H28 N O2
294.2416	33.49	---							
295.2439	6.78	---							
301.0766	27.34	---							
301.1426	32.63	301.1440	-1.4	-4.6	8.5	2577.4	n/a	n/a	C18 H21 O4
302.0801	5.23	---							
302.1464	5.98	---							
302.2703	6.14	---							
303.1434	6.12	---							
304.2861	97.48	---							
305.1589	7.79	---							
305.2898	18.56	---							
309.1843	6.51	---							
310.2359	9.45	---							
312.1936	10.54	312.1939	-0.3	-1.0	5.5	2695.8	n/a	n/a	C18 H27 N O2 Na
313.2354	14.71	---							
314.1740	100.00	---							
315.1785	23.48	---							
316.3220	7.59	---							
320.2591	12.17	---							
322.2715	5.74	322.2722	-0.7	-2.2	0.5	2689.6	n/a	n/a	C18 H37 N O2 Na
330.2047	9.04	330.2045	0.2	0.6	4.5	2599.3	n/a	n/a	C18 H29 N O3 Na
331.2122	18.15	---							
332.1476	5.33	---							
332.3155	8.31	---							
333.1578	5.86	---							
336.1220	14.62	336.1212	0.8	2.4	9.5	2811.6	n/a	n/a	C18 H19 N O4 Na

Figure S8. HRMS report for **19**.

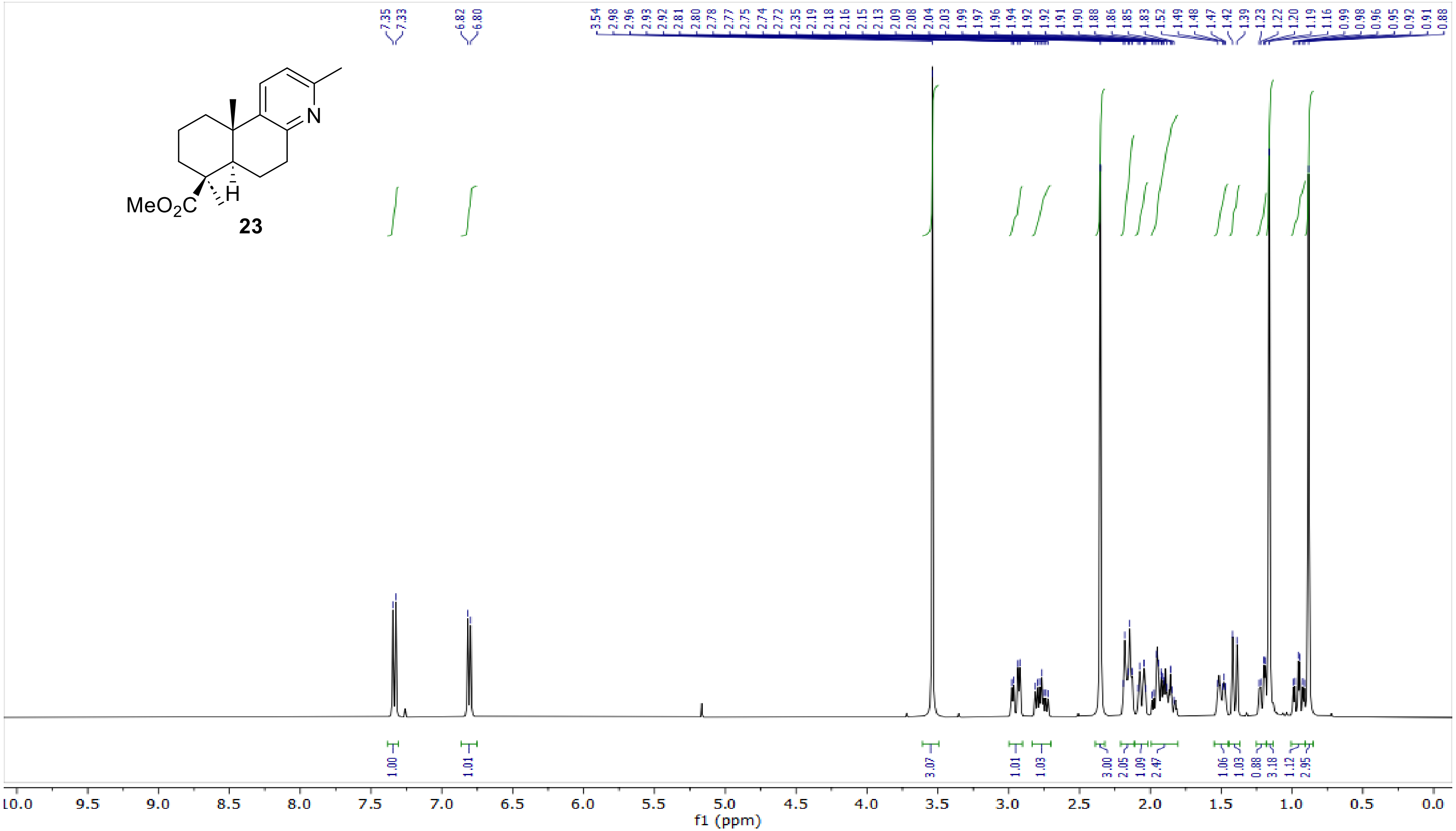

Figure S9. ^{1}H NMR spectrum of **23** ($CDCl_3$, 400 MHz).

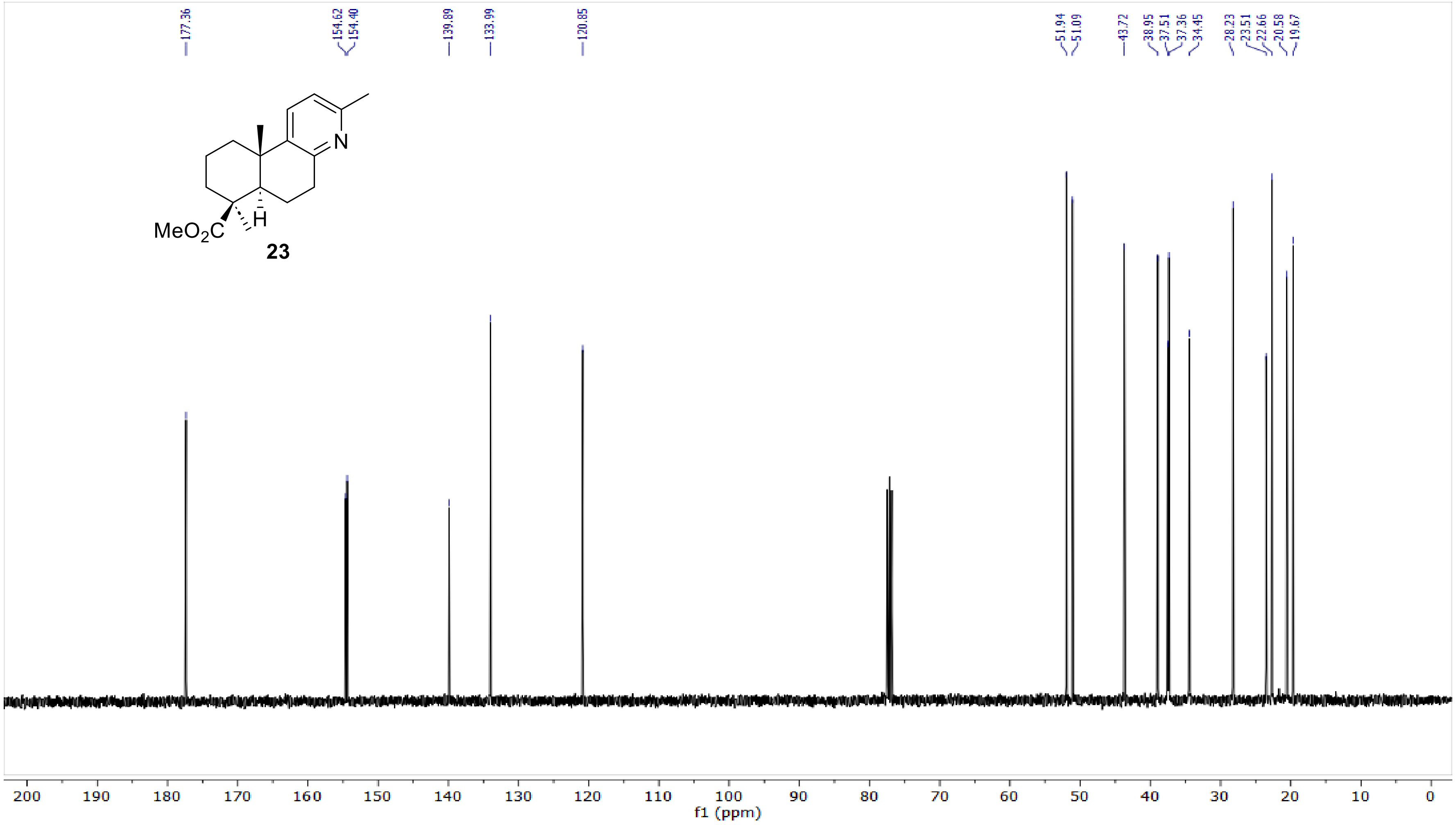

Figure S10. ^{13}C NMR spectrum of **23** ($CDCl_3$, 100 MHz).

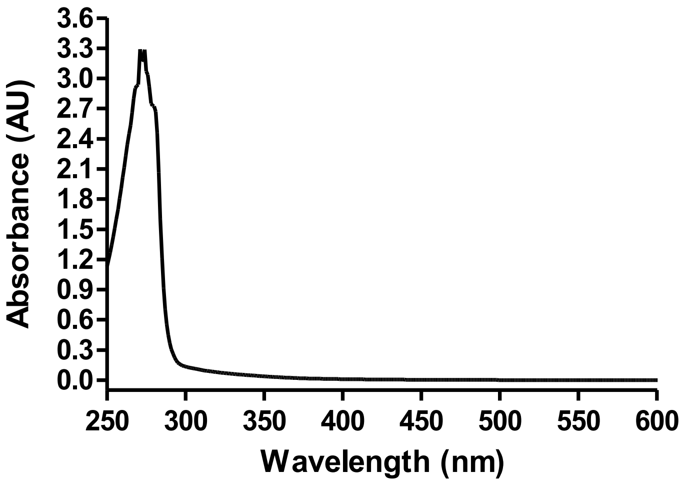

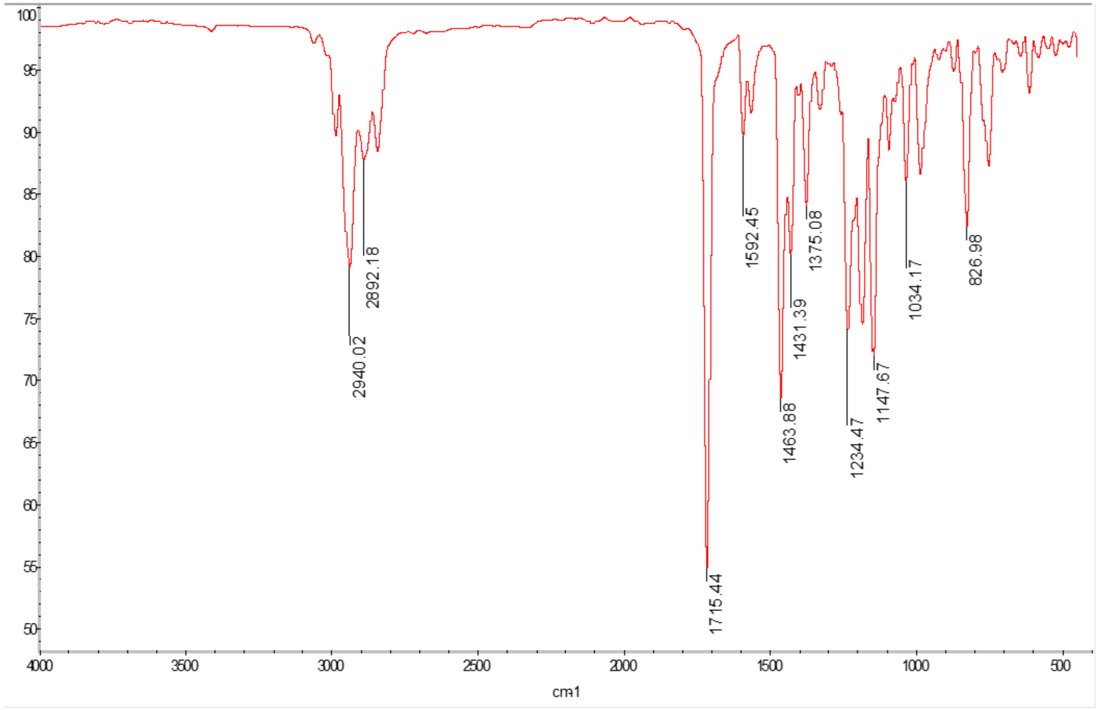

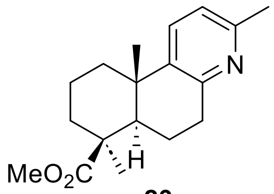

23

Figure S11. UV (in MeCN) and IR (KBr pellet) spectra report for **23**.

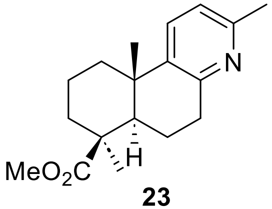

23

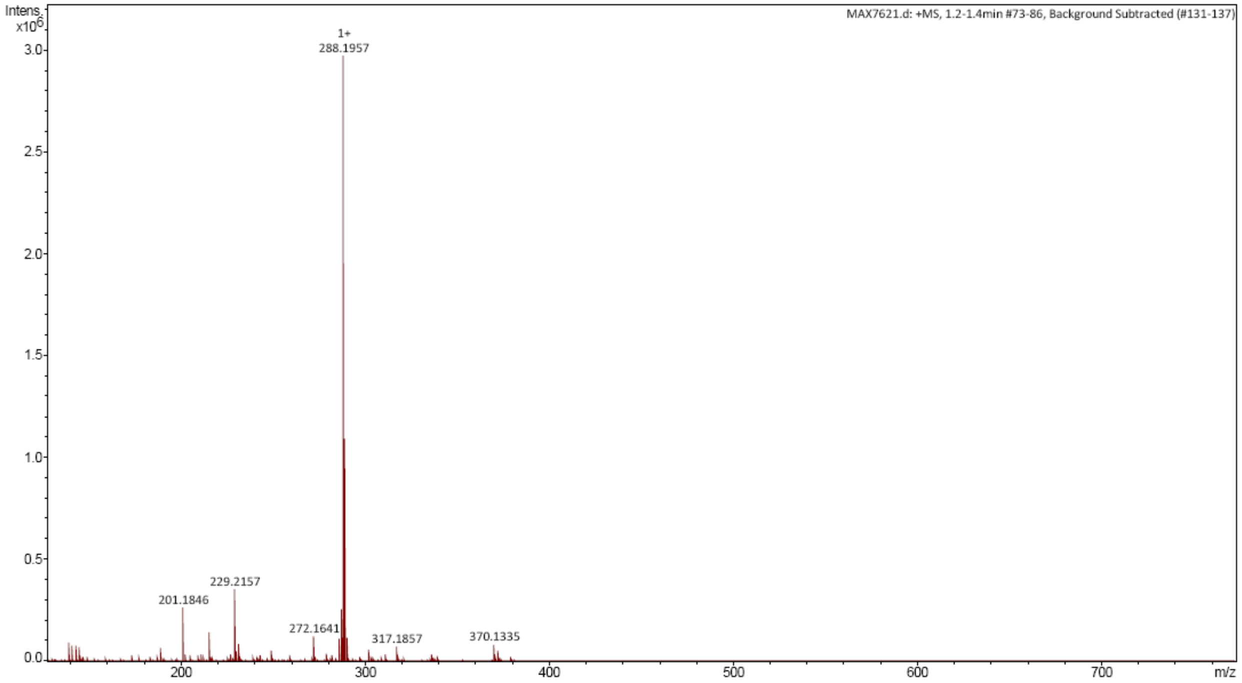

Meas. m/z	#	Ion Formula	m/z	err [ppm]	mSigma	# mSigma	Score	rdb	e⁻ Conf	N-Rule
288.1957	1	C18H26NO2	288.1958	0.4	90.0	1	100.00	7.0	even	ok

Figure S12. HRMS report for **23**.

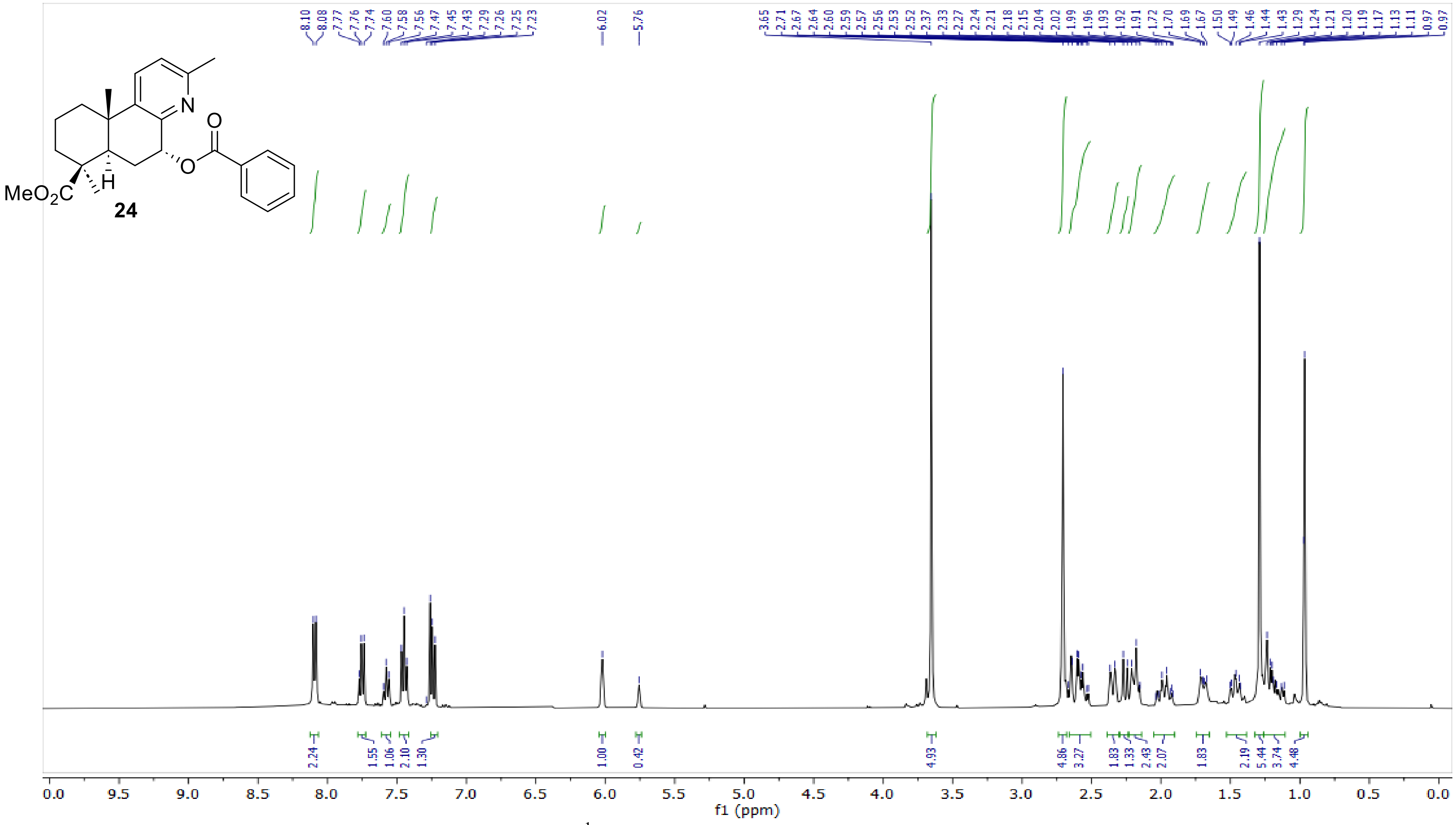

Figure S13. ^{1}H NMR spectrum of **24** ($CDCl_3$, 400 MHz).

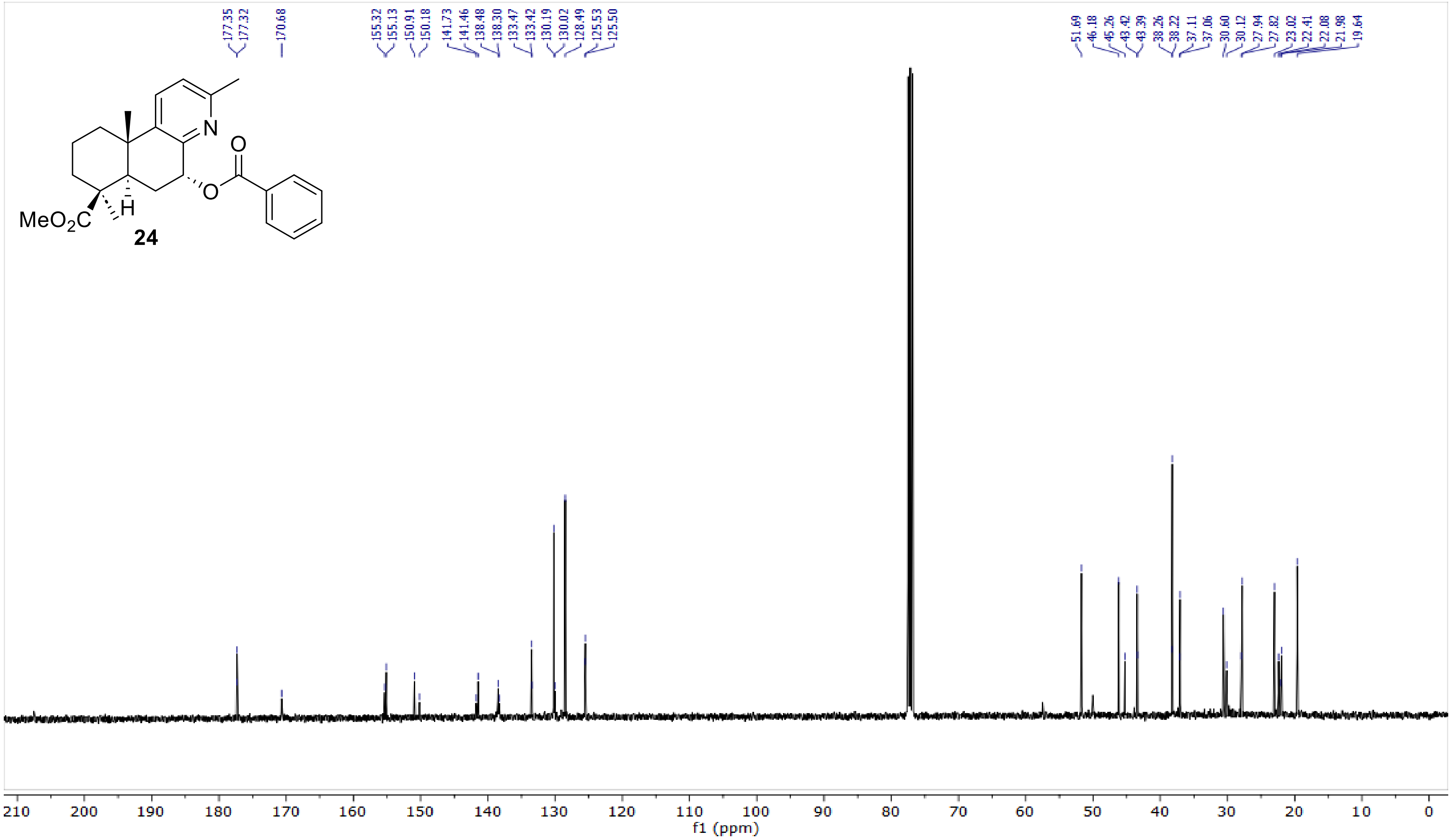

Figure S14. ^{13}C NMR spectrum of **24** ($CDCl_3$, 100 MHz).

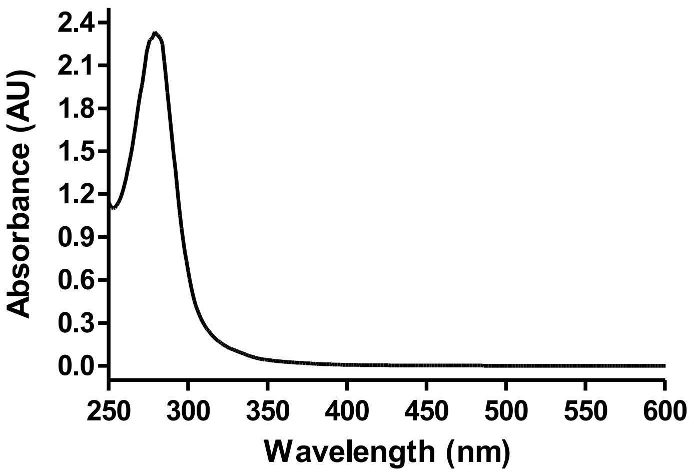

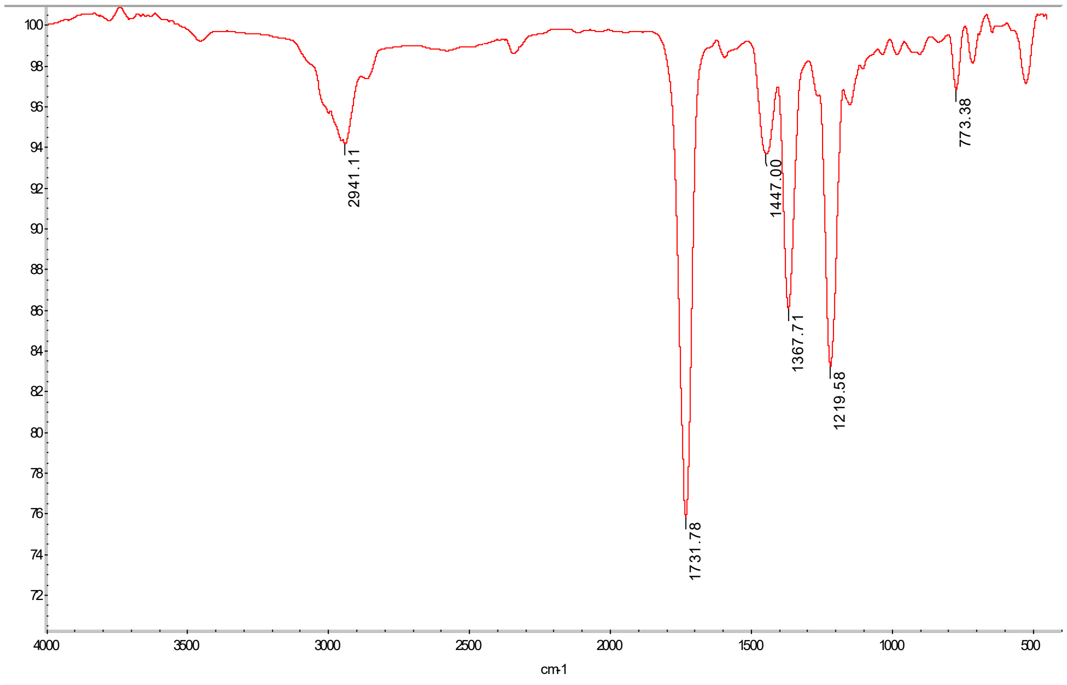

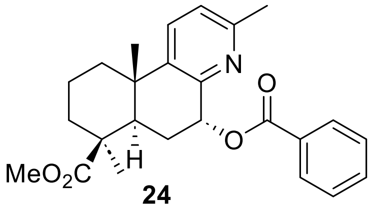

24

Figure S15. UV (in MeCN) and IR (KBr pellet) spectra report for **24**.

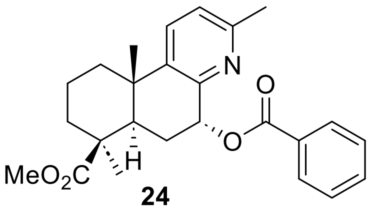

24

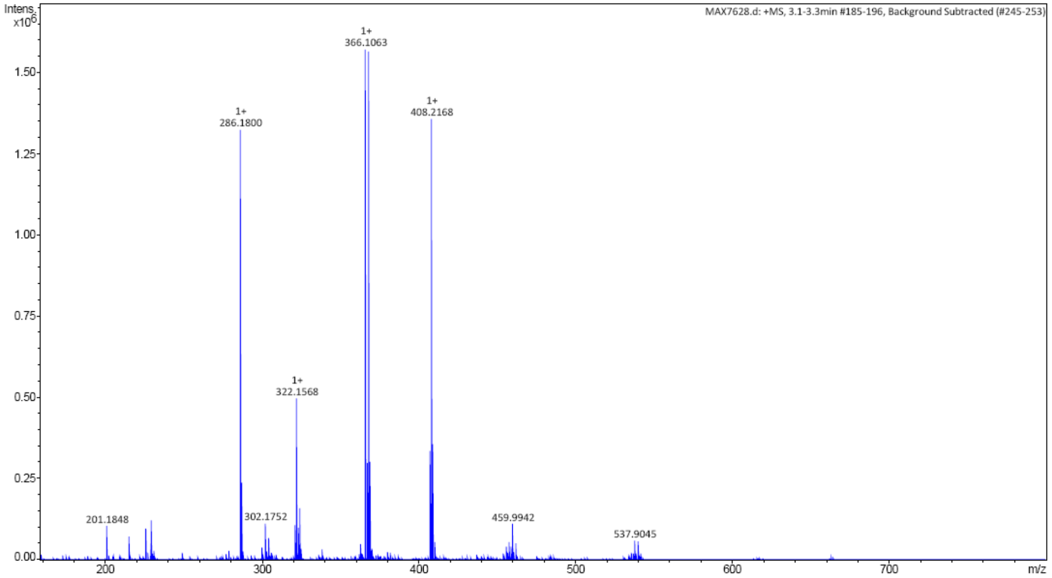

Meas. m/z	#	Ion Formula	m/z	err [ppm]	mSigma	# mSigma	Score	rdb	e¯ Conf	N-Rule
286.1800	1	C16H22N4O	286.1788	-4.0	12.4	1	69.26	8.5	odd	ok
	2	C18H24NO2	286.1802	0.7	18.1	2	100.00	8.0	even	ok
322.1568	1	C16H23ClN4O	322.1555	-3.9	11.4	1	43.73	7.5	odd	ok
	2	C18H25ClNO2	322.1568	0.3	11.4	2	100.00	7.0	even	ok
366.1063	1	C16H23BrN4O	366.1050	-3.5	3.2	1	55.41	7.5	odd	ok
	2	C18H25BrNO2	366.1063	0.1	6.1	2	100.00	7.0	even	ok
	3	C16H18N2O8	366.1058	-1.4	556.1	3	0.00	9.5	odd	ok
	4	C17H14N6O4	366.1071	2.3	558.4	4	0.00	14.5	odd	ok
	5	C14H16N5O7	366.1044	-5.0	558.5	5	0.00	10.0	even	ok
408.2168	1	C23H28N4O3	408.2156	-3.0	3.3	1	63.30	12.5	odd	ok
	2	C25H30NO4	408.2169	0.3	10.4	2	100.00	12.0	even	ok
	3	C26H26N5	408.2183	3.5	23.5	3	37.54	17.0	even	ok

Figure S16. HRMS report for **24**.

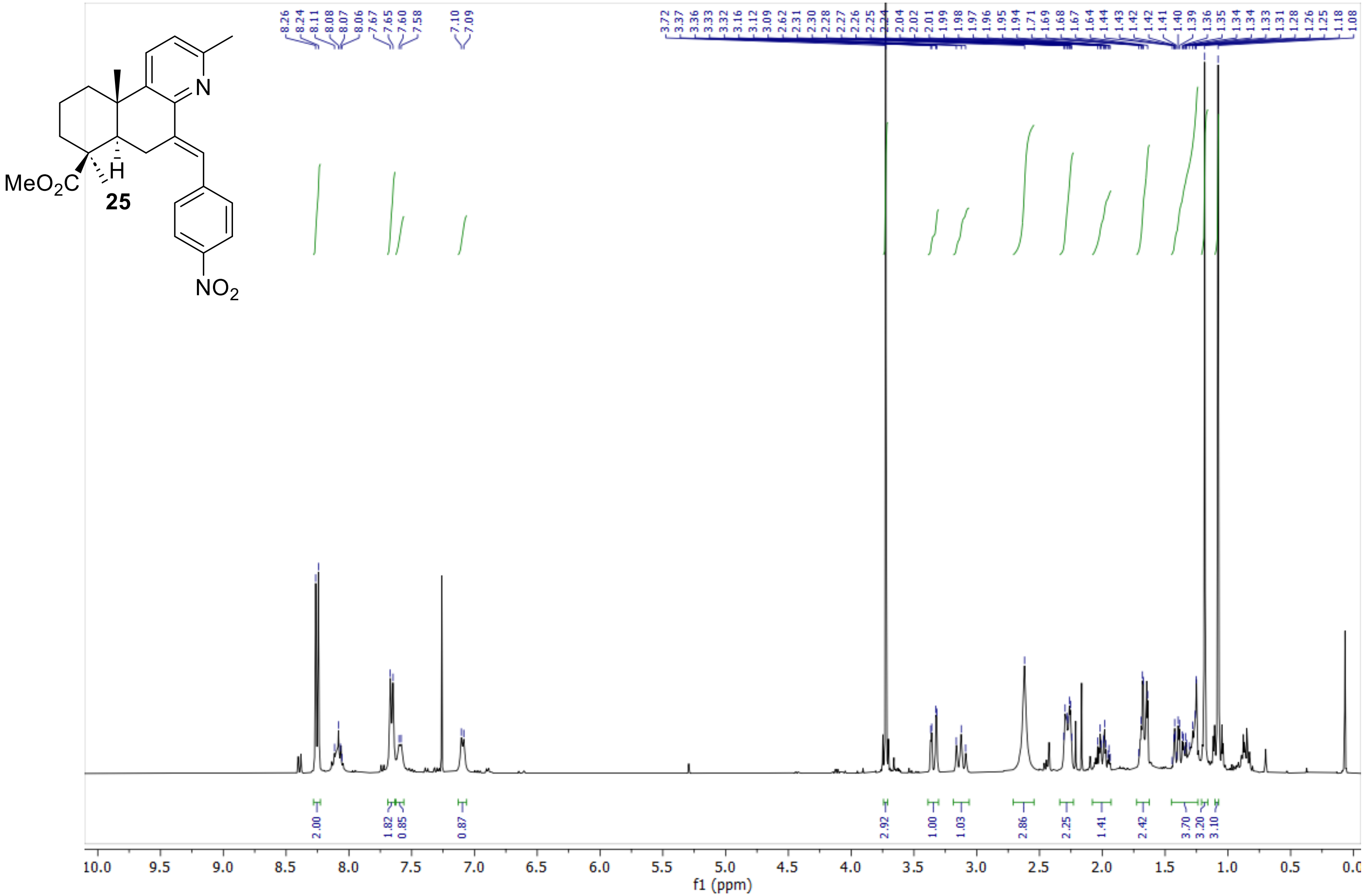

Figure S17. ^{1}H NMR spectrum of **25** ($CDCl_3$, 400 MHz).

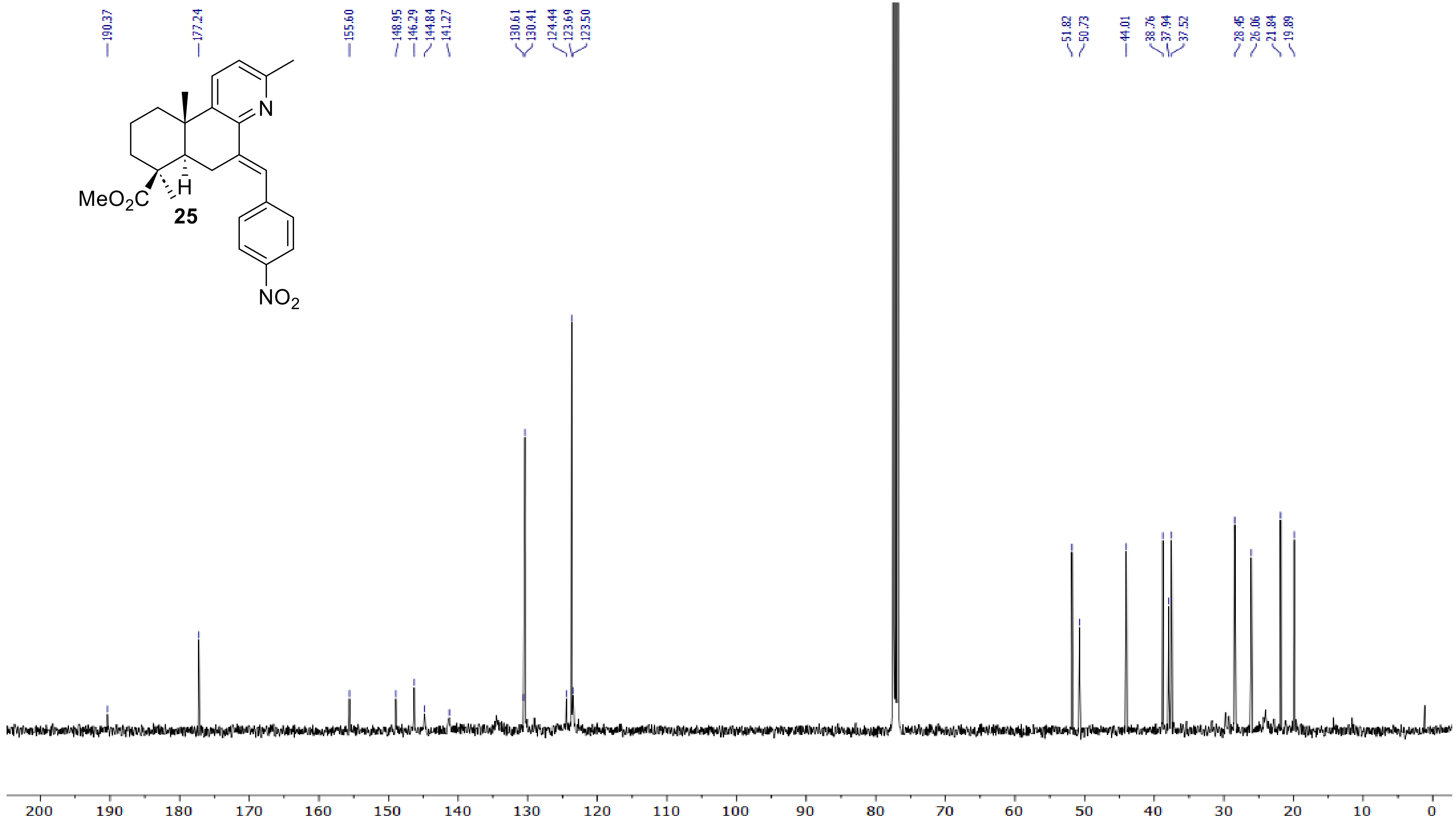

Figure S18. ^{13}C NMR spectrum of **25** ($CDCl_3$, 100 MHz).

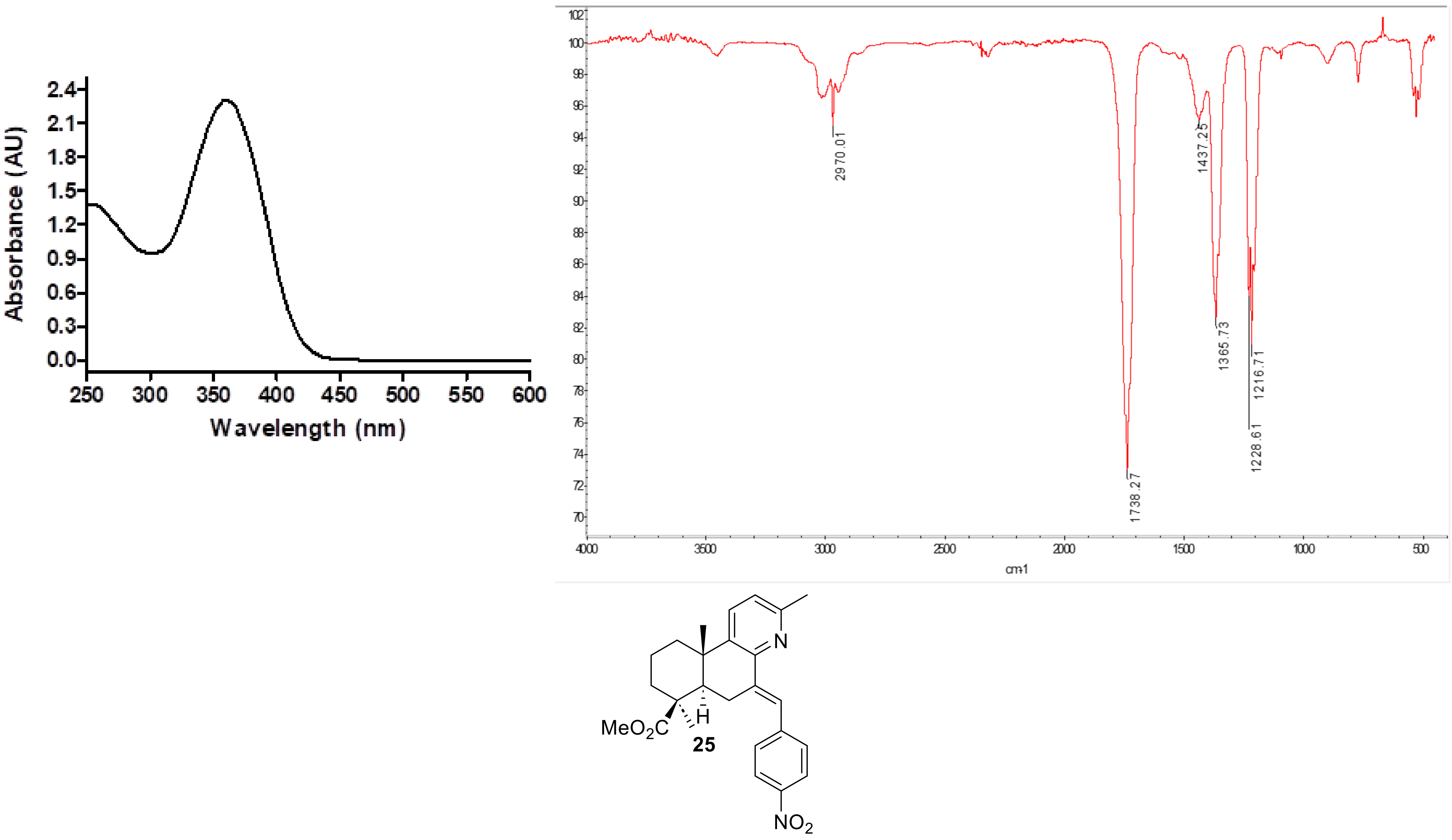

Figure S19. UV (in MeCN) and IR (KBr pellet) spectra report for **25**.

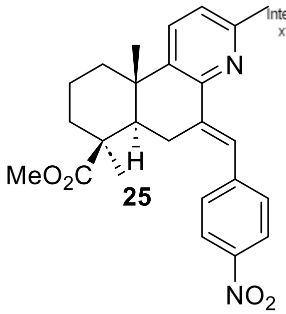

25

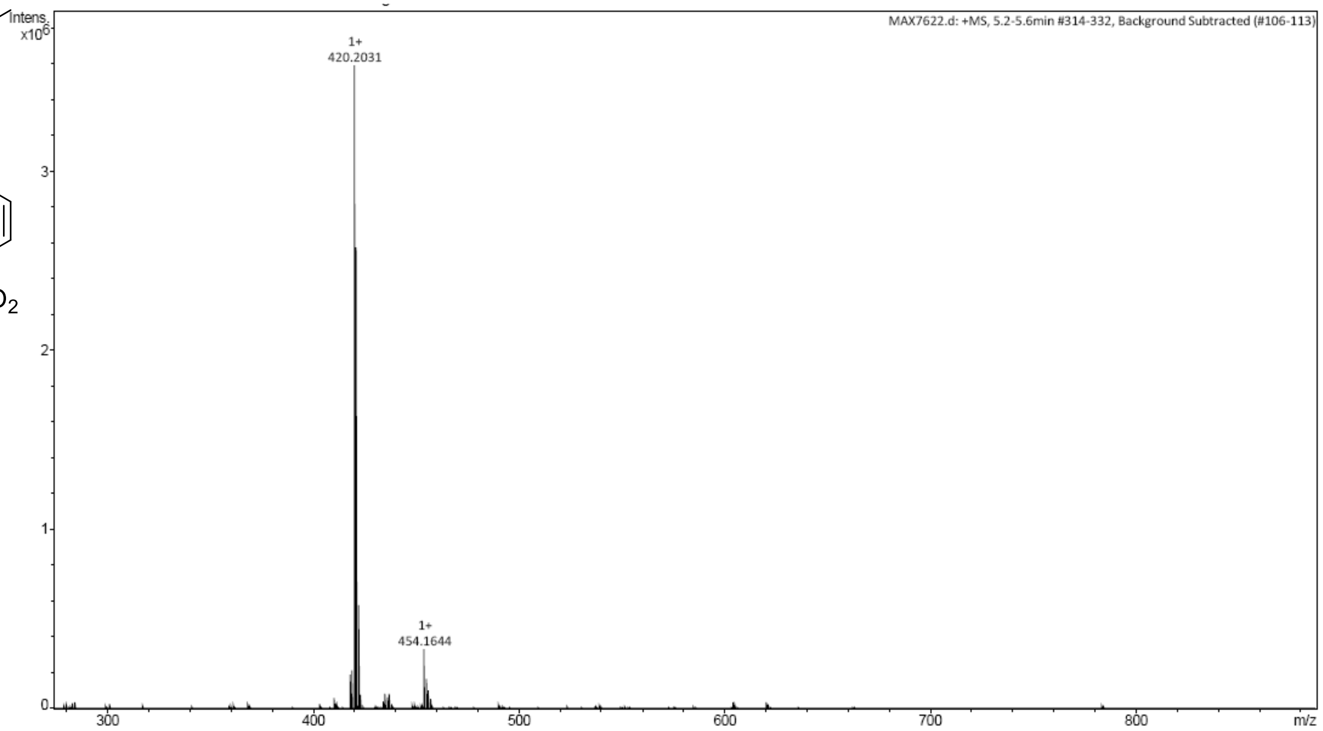

Meas. m/z	#	Ion Formula	m/z	err [ppm]	mSigma	# mSigma	Score	rdb	e⁻ Conf	N-Rule
420.2031	1	C25H28N2O4	420.2044	3.0	317.6	1	100.00	13.5	odd	ok
	2	C23H26N5O3	420.2030	-0.2	324.6	2	73.76	14.0	even	ok
	3	C22H30NO7	420.2017	-3.3	337.4	3	6.54	9.0	even	ok
454.1644	1	C28H24NO5	454.1649	1.1	358.8	1	100.00	18.0	even	ok
	2	C26H22N4O4	454.1636	-1.8	364.7	2	35.56	18.5	odd	ok
	3	C25H26O8	454.1622	-4.8	374.5	3	3.31	13.5	odd	ok

Figure S20. HRMS report for **25**.

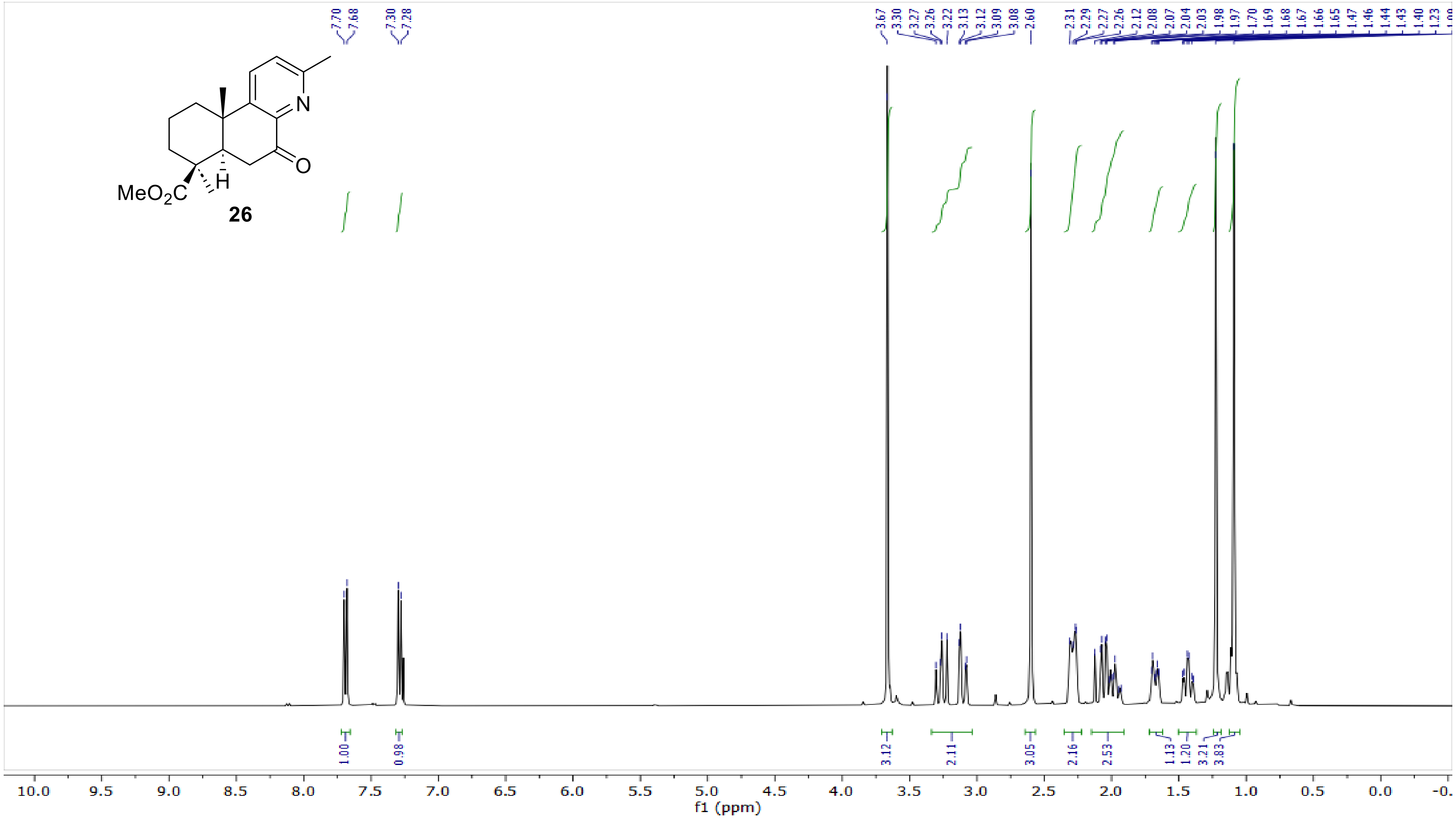

Figure S21. ^{1}H NMR spectrum of **26** ($CDCl_3$, 400 MHz).

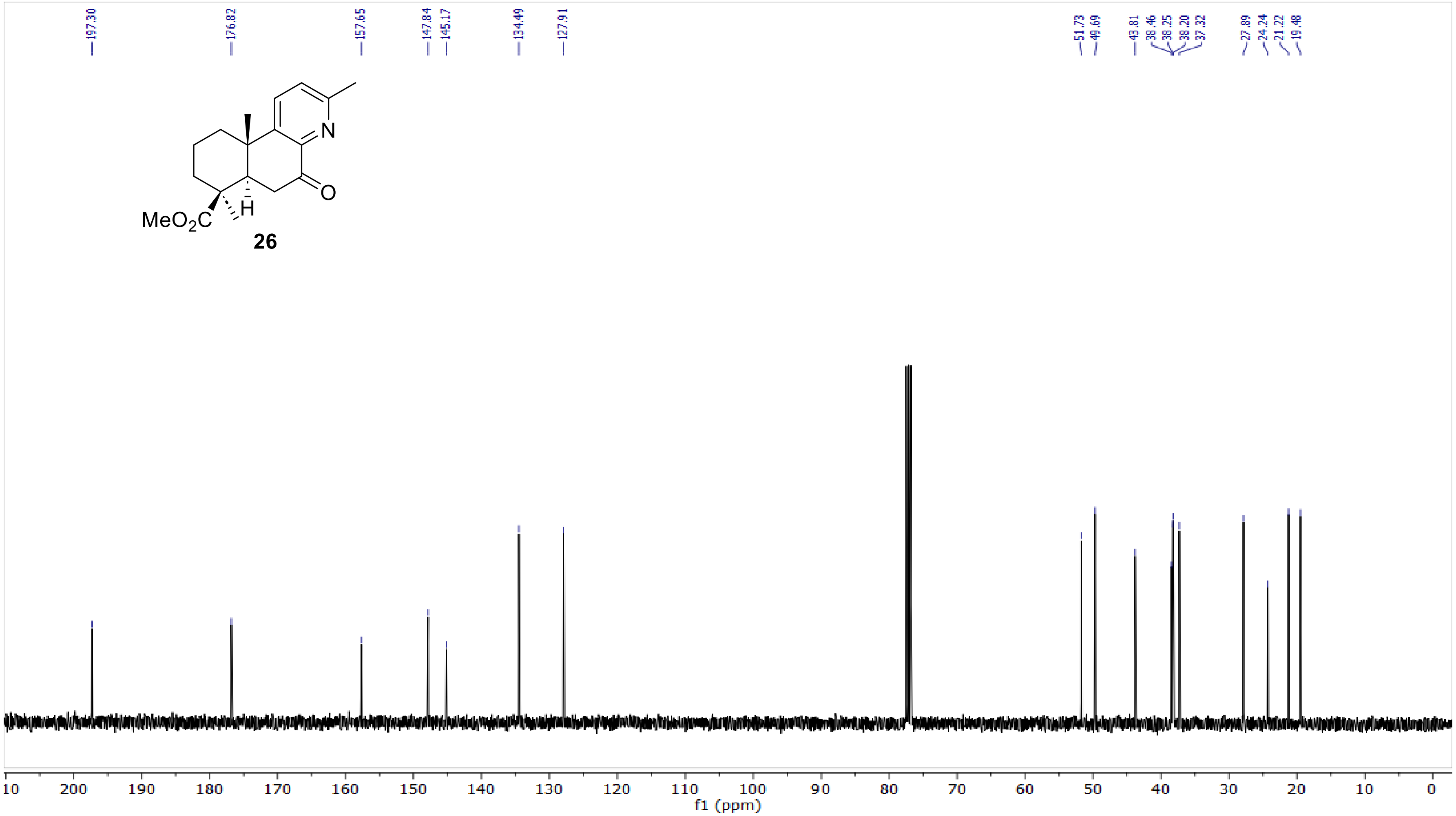

Figure S22. ^{13}C NMR spectrum of **26** ($CDCl_3$, 100 MHz).

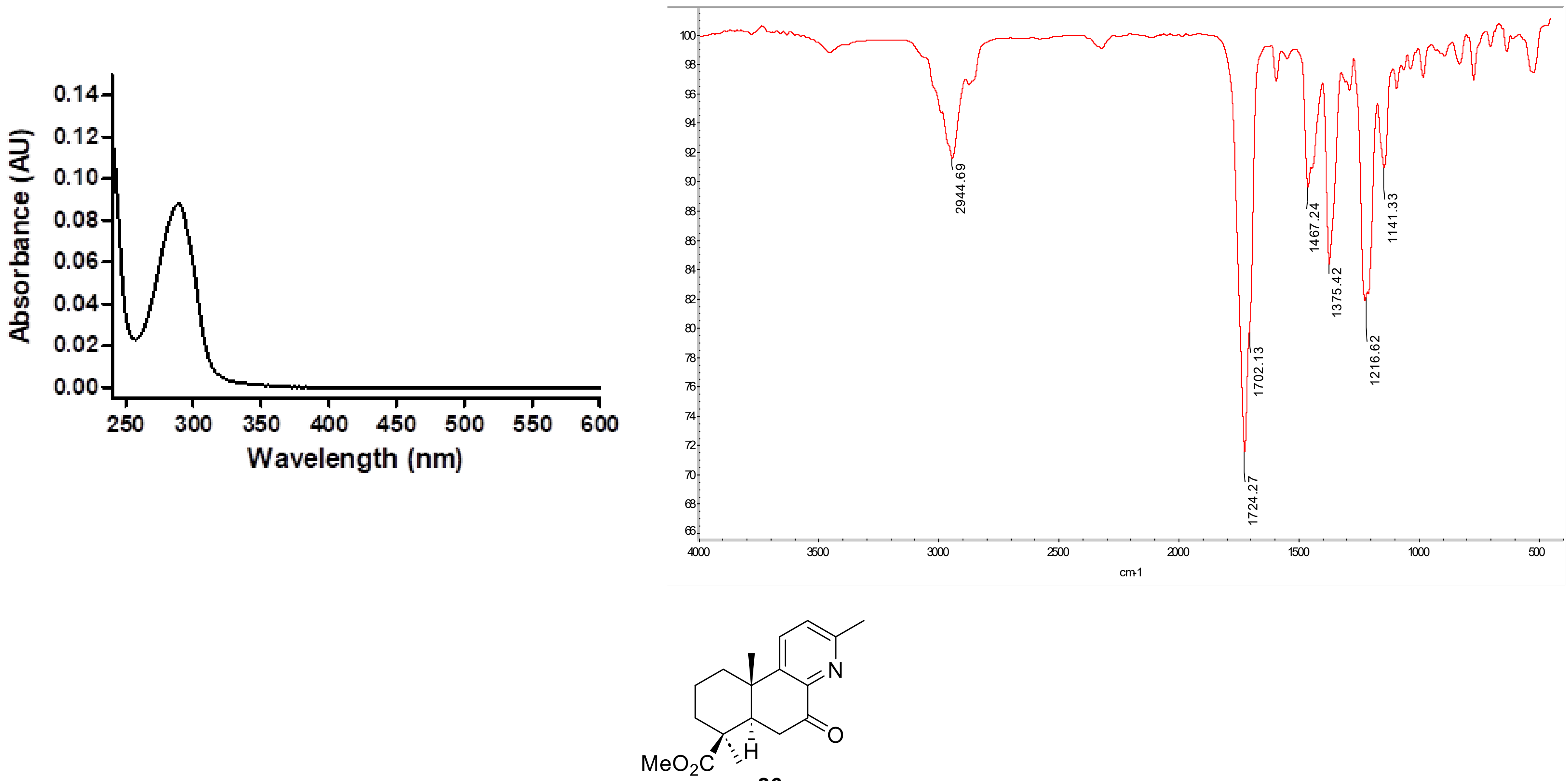

26

Figure S23. UV (in MeCN) and IR (KBr pellet) spectra report for **26**.

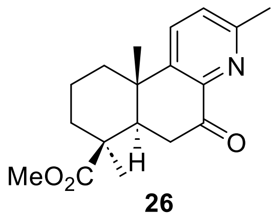

26

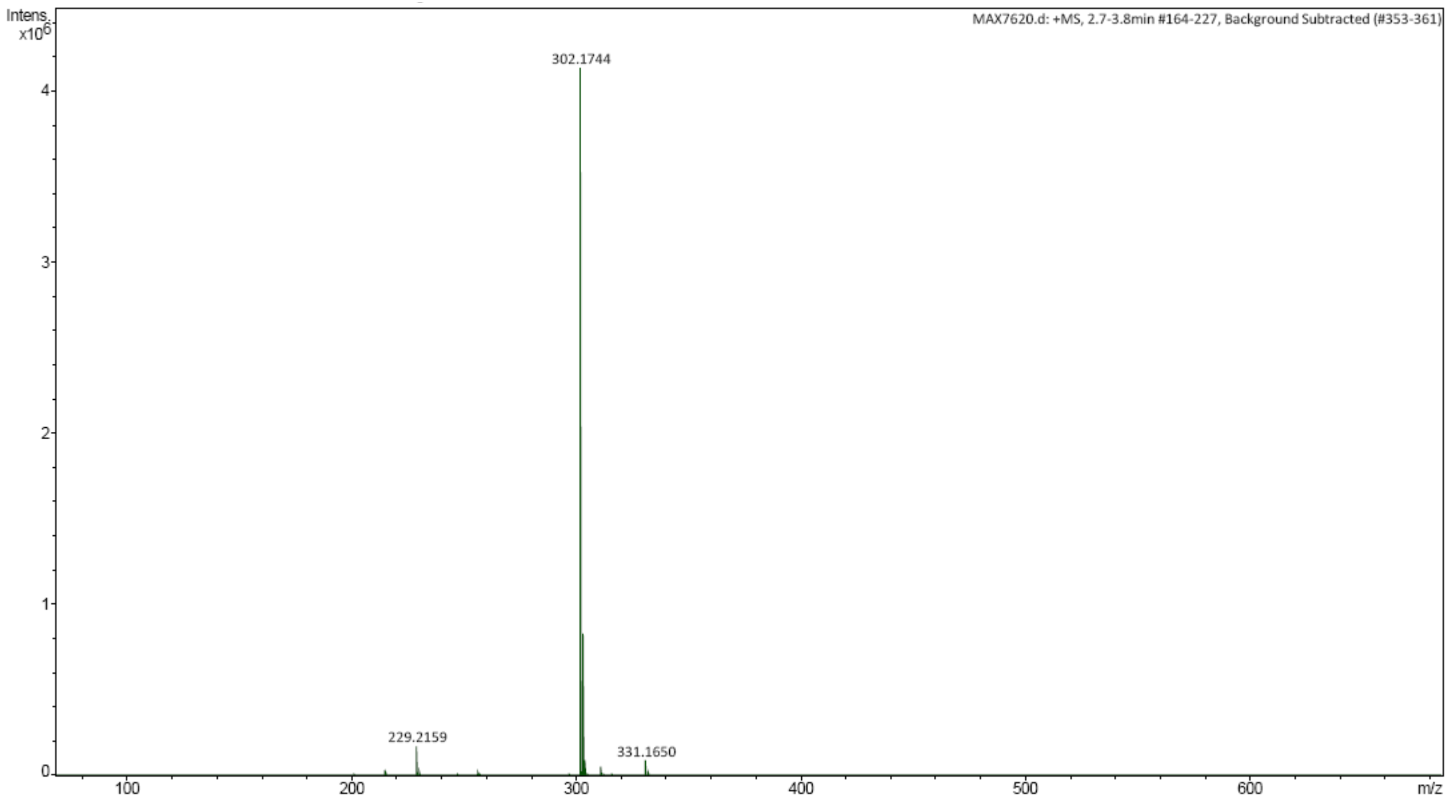

Meas. m/z	#	Ion Formula	m/z	err [ppm]	mSigma	# mSigma	Score	rdb	e⁻ Conf	N-Rule
302.1744	1	C16H22N4O2	302.1737	-2.1	13.7	1	100.00	8.5	odd	ok
	2	C18H24NO3	302.1751	2.3	14.8	2	95.15	8.0	even	ok
331.1651	1	C18H23N2O4	331.1652	0.5	17.5	1	100.00	9.0	even	ok

Figure S24. HRMS report for **26**.

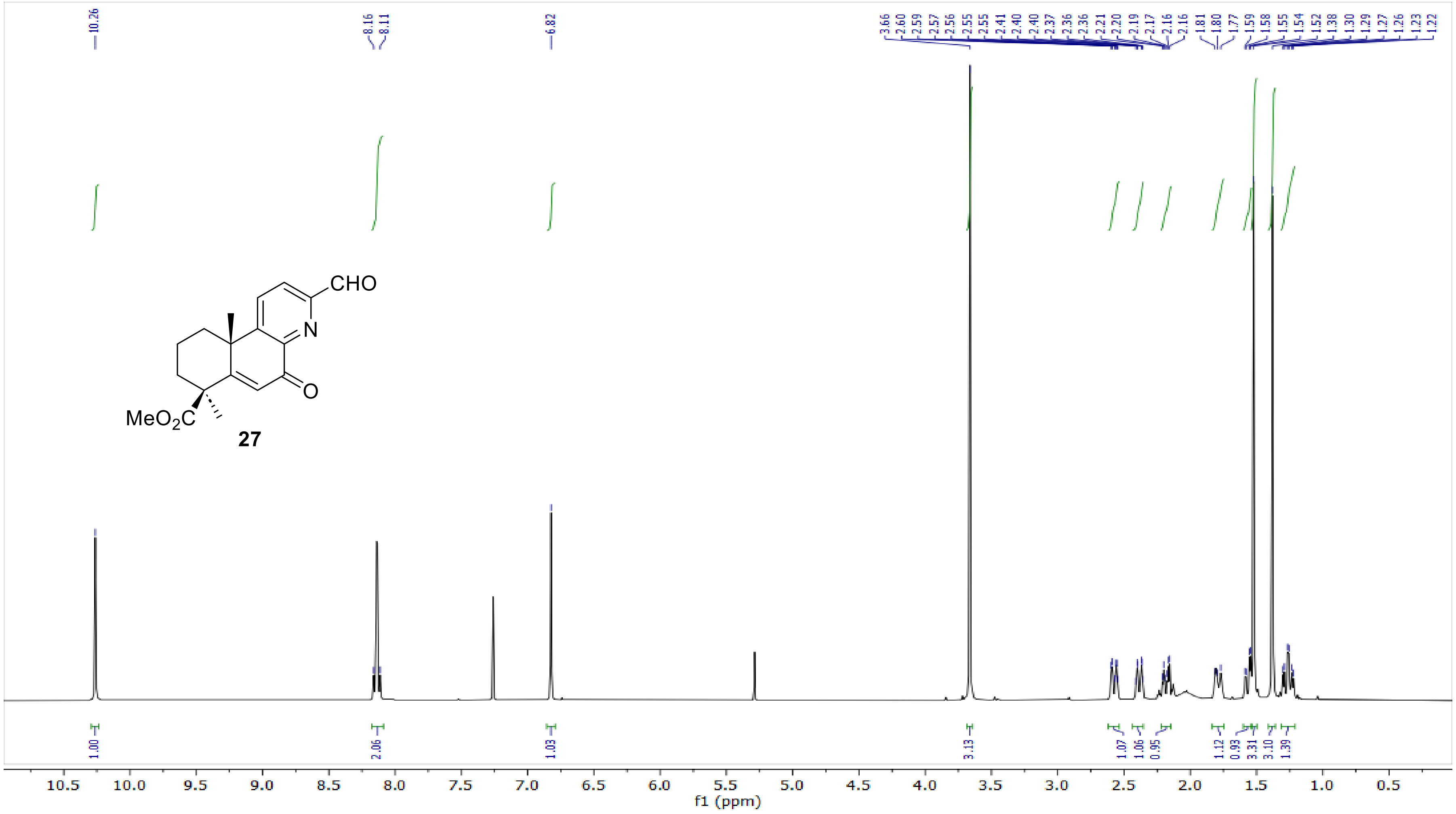

Figure S25. ^{1}H NMR spectrum of **27** ($CDCl_3$, 400 MHz).

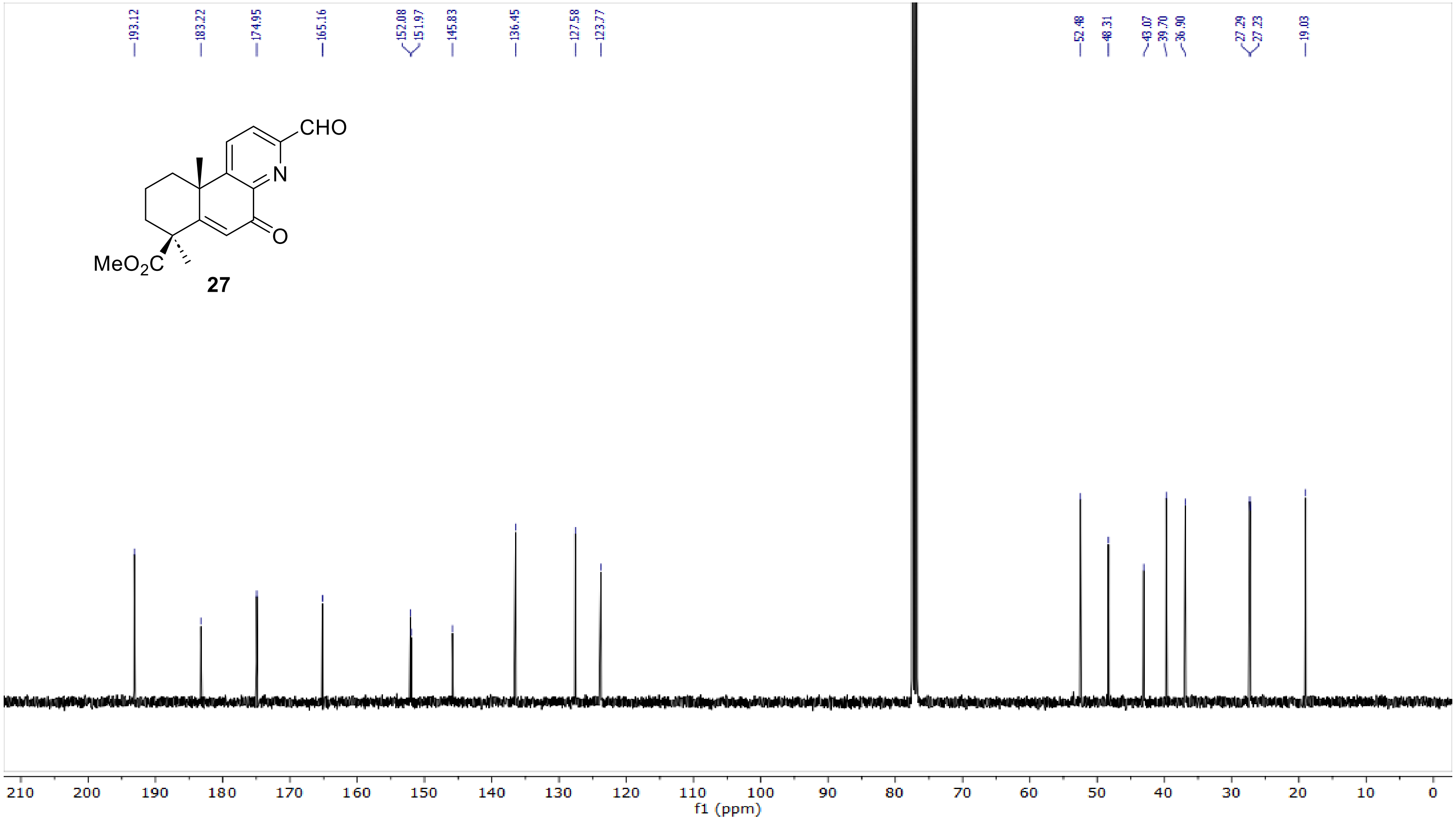

Figure S26. ^{13}C NMR spectrum of **27** ($CDCl_3$, 100 MHz).

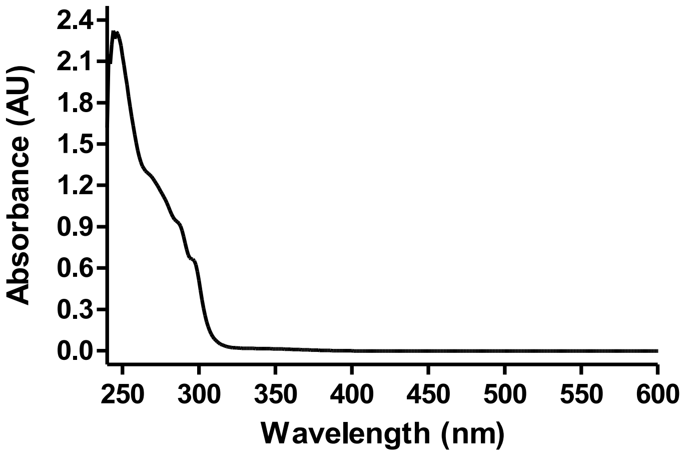

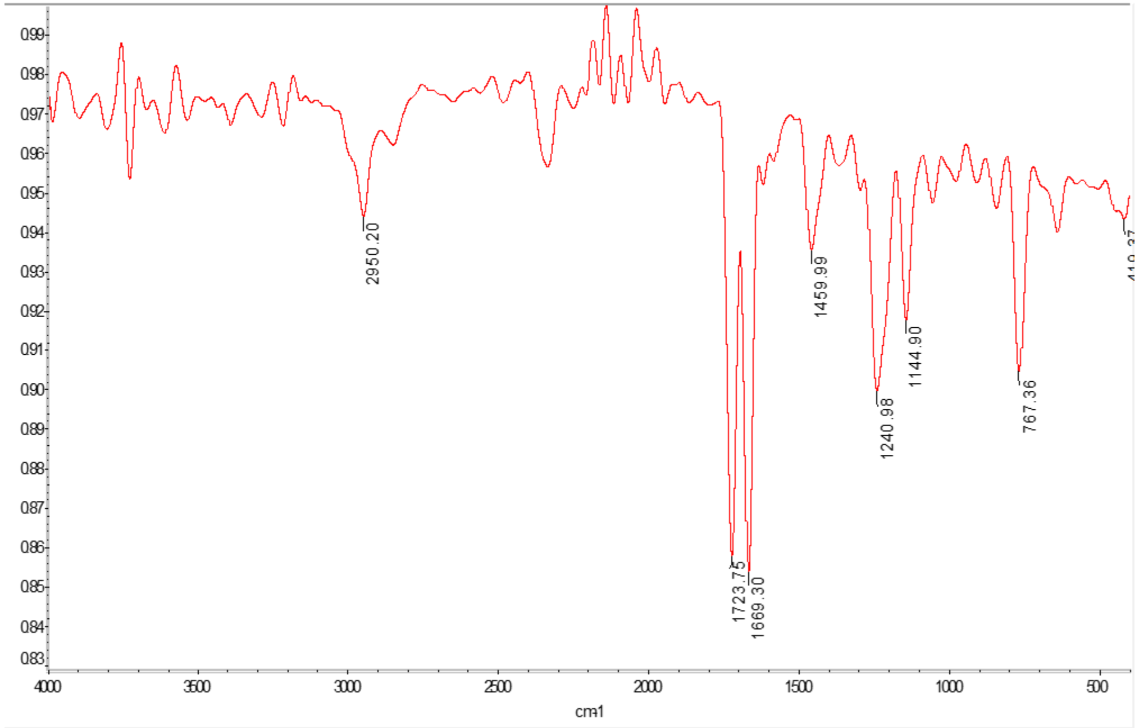

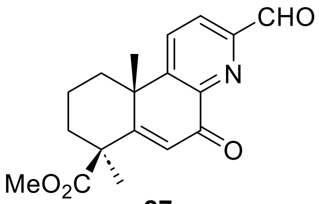

27

Figure S27. UV (in MeCN) and IR (thin film) spectra report for **27**.

Elemental Composition Report

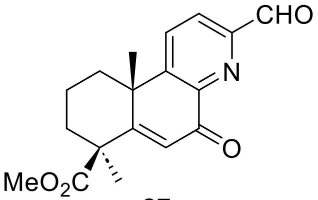

27

Multiple Mass Analysis: 4 mass(es) processed

Tolerance = 5.0 PPM / DBE: min = -400.0, max = 400.0
Element prediction: Off
Number of isotope peaks used for i-FIT = 3

Monoisotopic Mass, Even Electron Ions
54 formula(e) evaluated with 1 results within limits (all results (up to 1000) for each mass)
Elements Used:
C: 18-18 H: 0-50 N: 0-1 O: 0-5 Na: 0-1

JJRac10-bis 6.00000000
JJRac10-bis 15 (0.567) Cm (12:25) 1: TOF MS ES+
2.75e+009

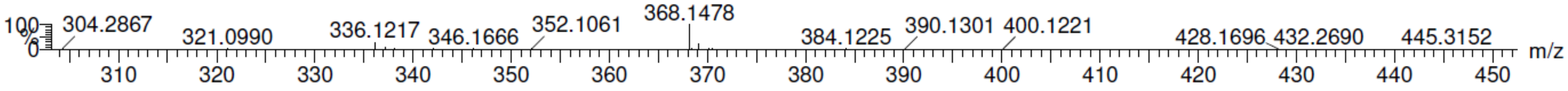

Minimum:	5.00				-400.0				
Maximum:	100.00		3.0	5.0	400.0				
Mass	RA	Calc. Mass	mDa	PPM	DBE	i-FIT	Norm	Conf(%)	Formula
336.1217	26.94	336.1212	0.5	1.5	9.5	2906.4	n/a	n/a	C18 H19 N O4 Na
337.1249	5.64	---							
368.1478	100.00	---							
369.1513	20.19	---							

Figure S28. HRMS report for **27**.

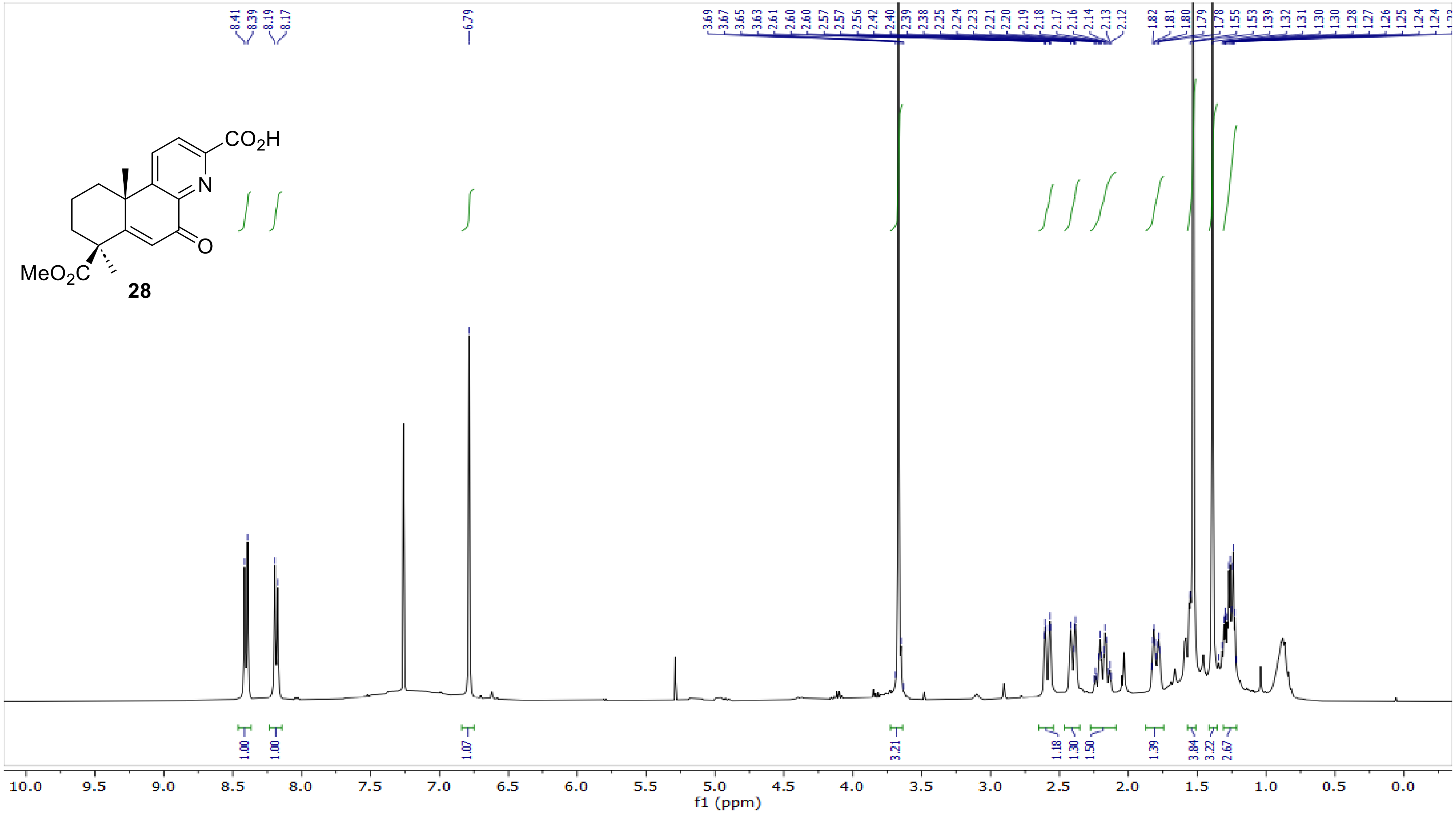

Figure S29. ^{1}H NMR spectrum of **28** ($CDCl_3$, 400 MHz).

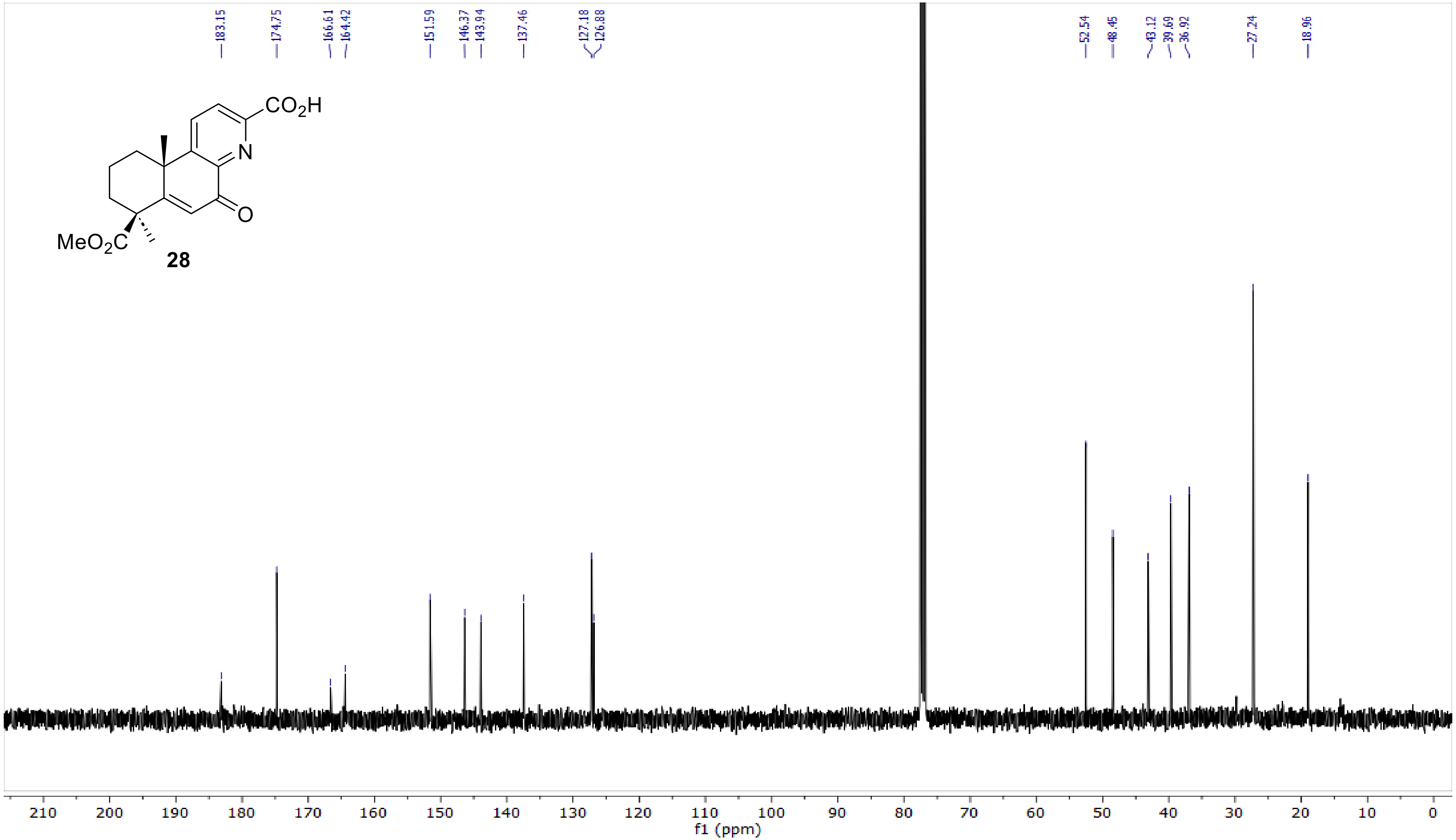

Figure S30. ^{13}C NMR spectrum of **28** ($CDCl_3$, 100 MHz).

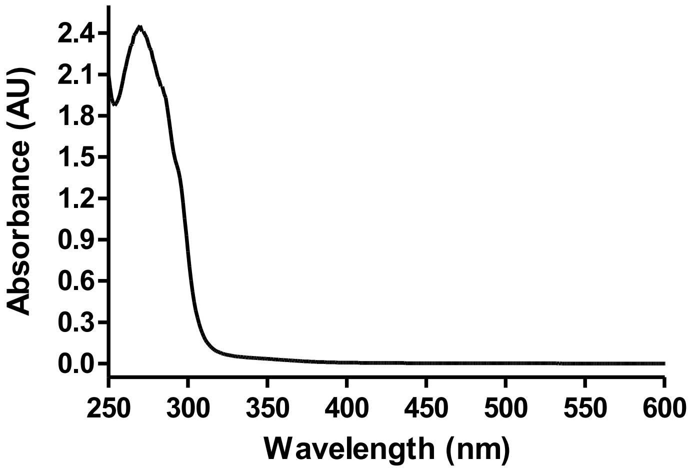

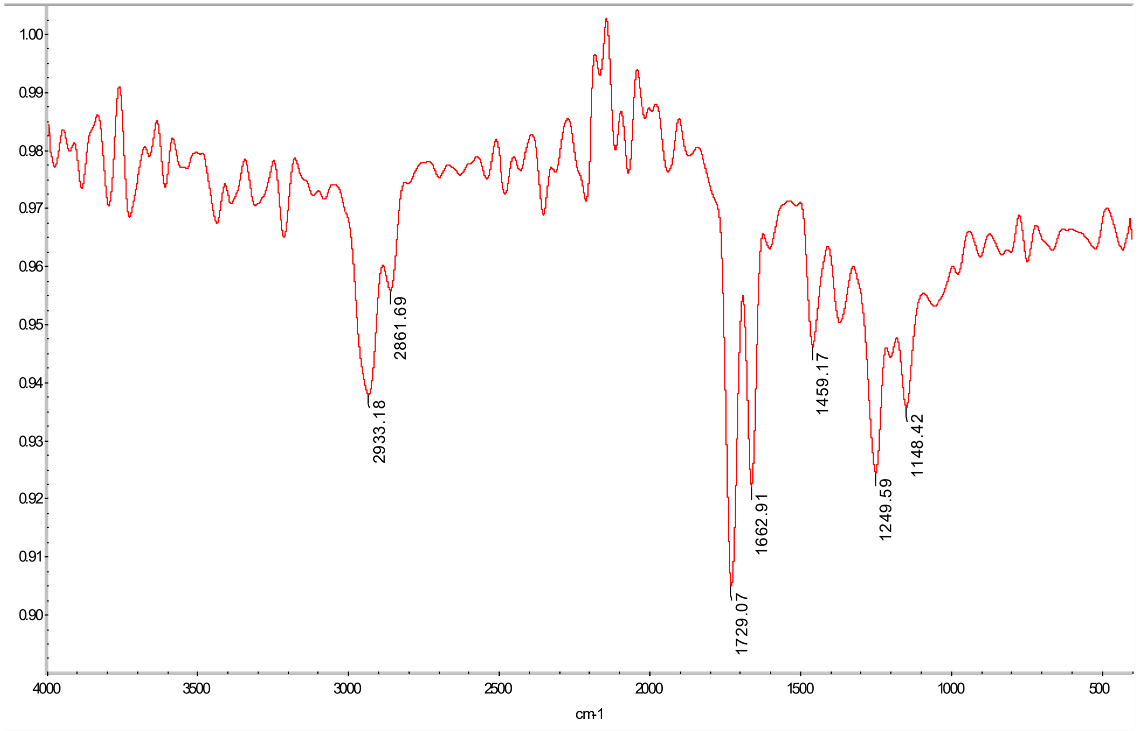

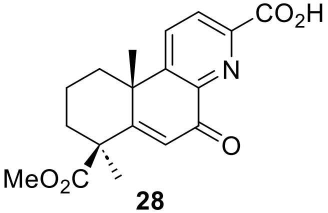

28

Figure S31. UV (in MeCN) and IR (KBr pellet) spectra report for **28**.

Elemental Composition Report

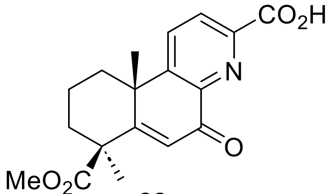

28

Multiple Mass Analysis: 10 mass(es) processed

Tolerance = 5.0 PPM / DBE: min = -400.0, max = 400.0
Element prediction: Off
Number of isotope peaks used for i-FIT = 3

Monoisotopic Mass, Even Electron Ions
160 formula(e) evaluated with 5 results within limits (all results (up to 1000) for each mass)
Elements Used:
C: 18-18 H: 0-50 N: 0-1 O: 0-5 Na: 0-1

JJRac5-bis 6.00000000
JJRac5-bis 64 (2.422) Cm (60:65) 1: TOF MS ES+ 4.54e+008

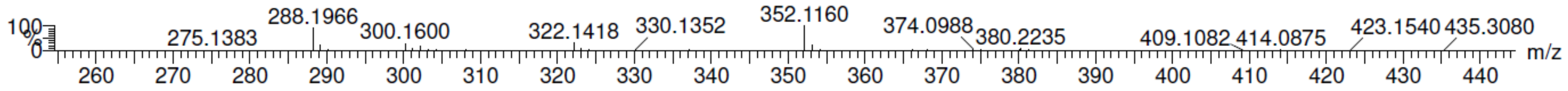

Minimum:	5.00				-400.0				
Maximum:	100.00		3.0	5.0	400.0				
Mass	RA	Calc. Mass	mDa	PPM	DBE	i-FIT	Norm	Conf(%)	Formula
288.1966	89.71	288.1964	0.2	0.7	6.5	2553.8	n/a	n/a	C18 H26 N O2
289.1997	19.47	---							
300.1600	27.95	300.1600	0.0	0.0	8.5	2382.3	n/a	n/a	C18 H22 N O3
301.1629	5.91	---							
302.1752	15.29	302.1756	-0.4	-1.3	7.5	1880.9	n/a	n/a	C18 H24 N O3
322.1418	30.64	322.1419	-0.1	-0.3	8.5	2368.8	n/a	n/a	C18 H21 N O3 Na
323.1449	6.44	---							
352.1160	100.00	352.1161	-0.1	-0.3	9.5	2451.3	n/a	n/a	C18 H19 N O5 Na
353.1193	20.65	---							
380.2235	6.93	---							

Figure S32. HRMS report for **28**.

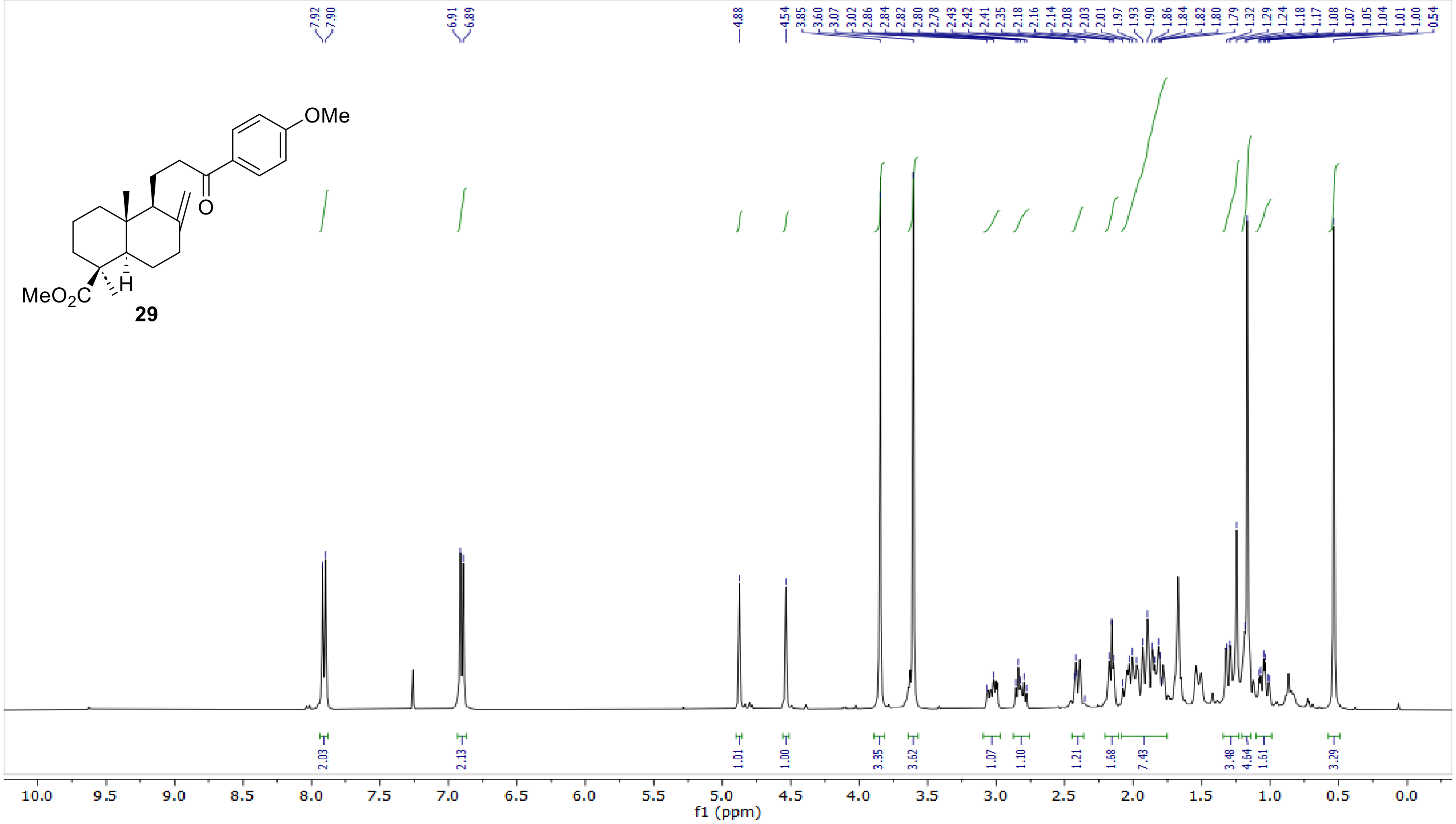

Figure S33. ^{1}H NMR spectrum of **29** ($CDCl_3$, 400 MHz).

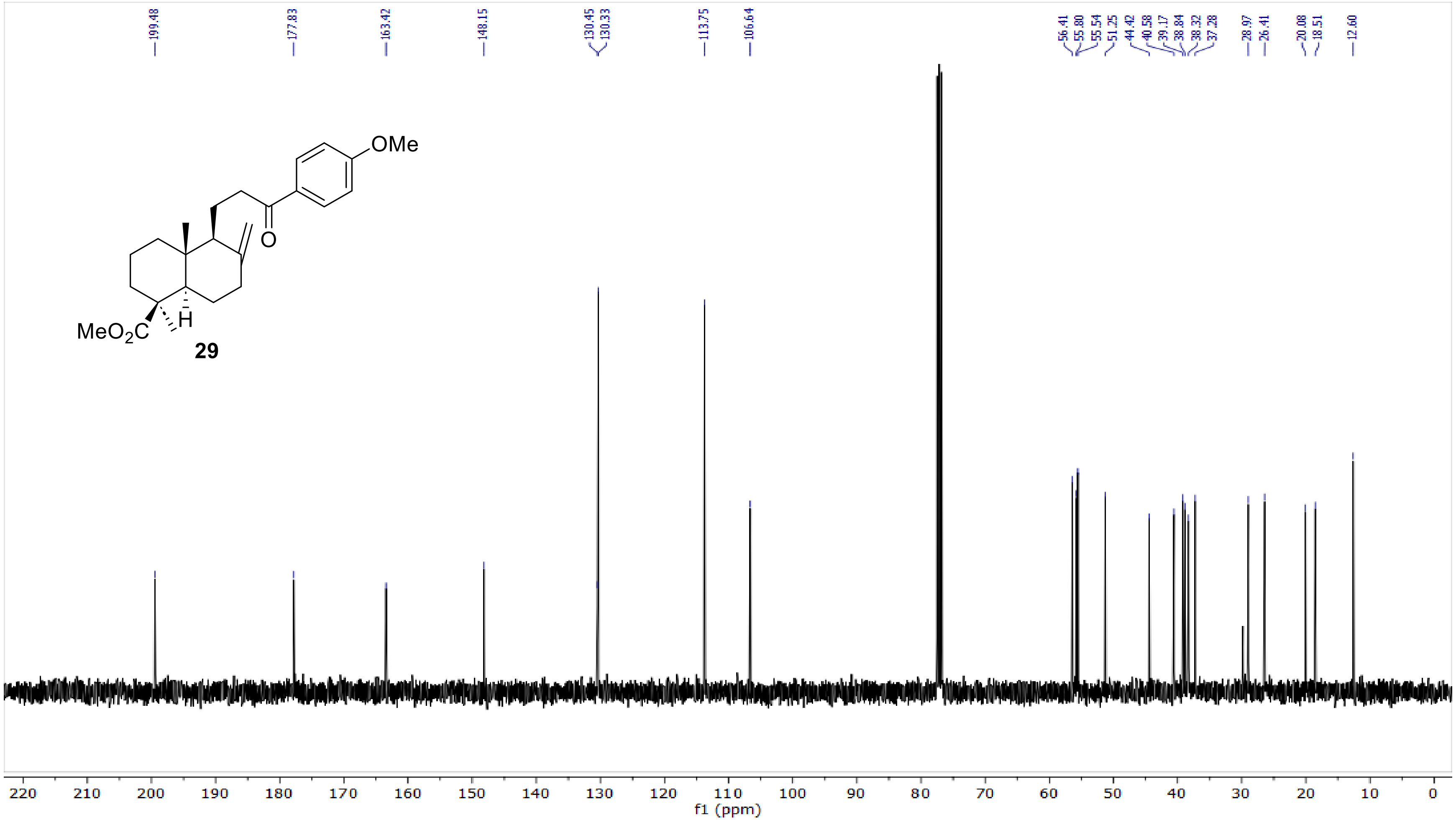

Figure S34. ^{13}C NMR spectrum of **29** ($CDCl_3$, 100 MHz).

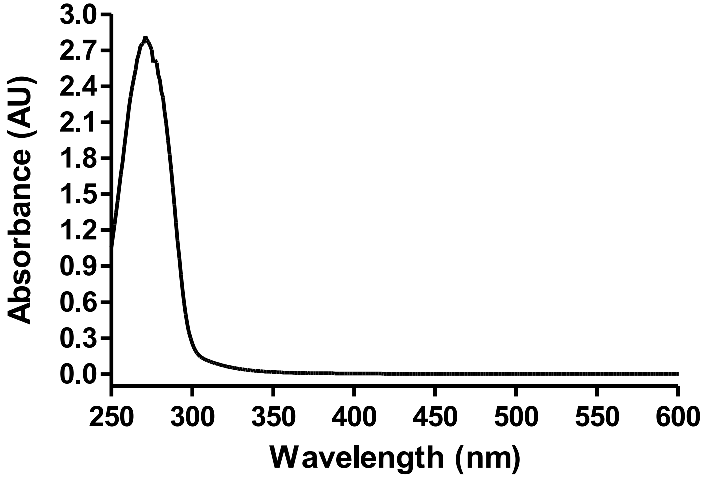

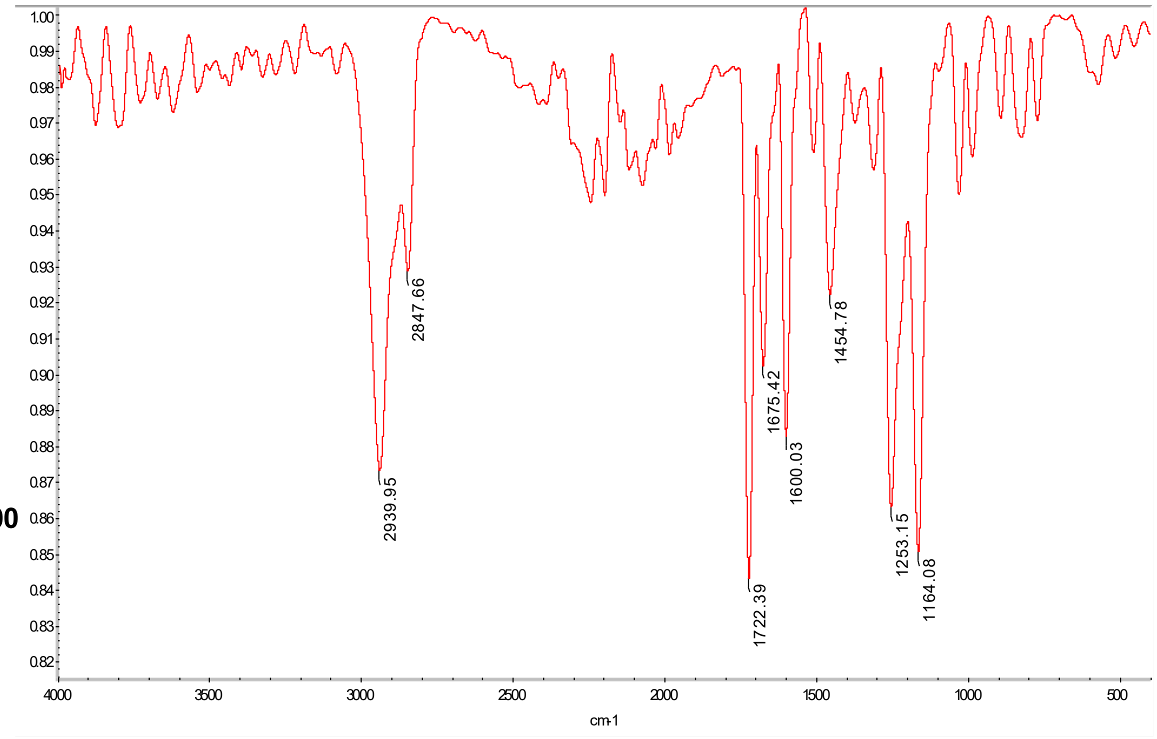

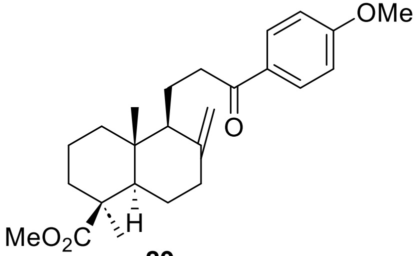

29

Figure S35. UV (in MeCN) and IR (thin film) spectra report for **29**.

Elemental Composition Report

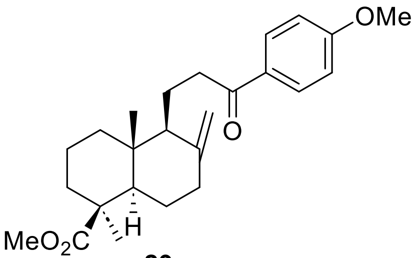

29

Single Mass Analysis

Tolerance = 5.0 PPM / DBE: min = -400.0, max = 400.0
Element prediction: Off
Number of isotope peaks used for i-FIT = 3

Monoisotopic Mass, Even Electron Ions
4 formula(e) evaluated with 1 results within limits (all results (up to 1000) for each mass)
Elements Used:
C: 23-25 H: 0-40 O: 1-5 Na: 0-1

JJRac14 6.00000000
JJRac14 31 (1.187) Cm (31:44) 1: TOF MS ES+
3.70e+007

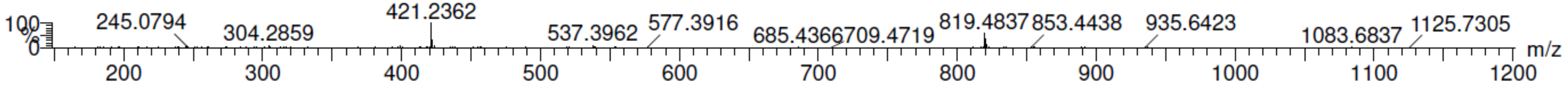

Minimum:				-400.0				
Maximum:		3.0	5.0	400.0				
Mass	Calc. Mass	mDa	PPM	DBE	i-FIT	Norm	Conf(%)	Formula
421.2362	421.2355	0.7	1.7	8.5	2003.1	n/a	n/a	C25 H34 O4 Na

Figure S36. HRMS report for **29**.

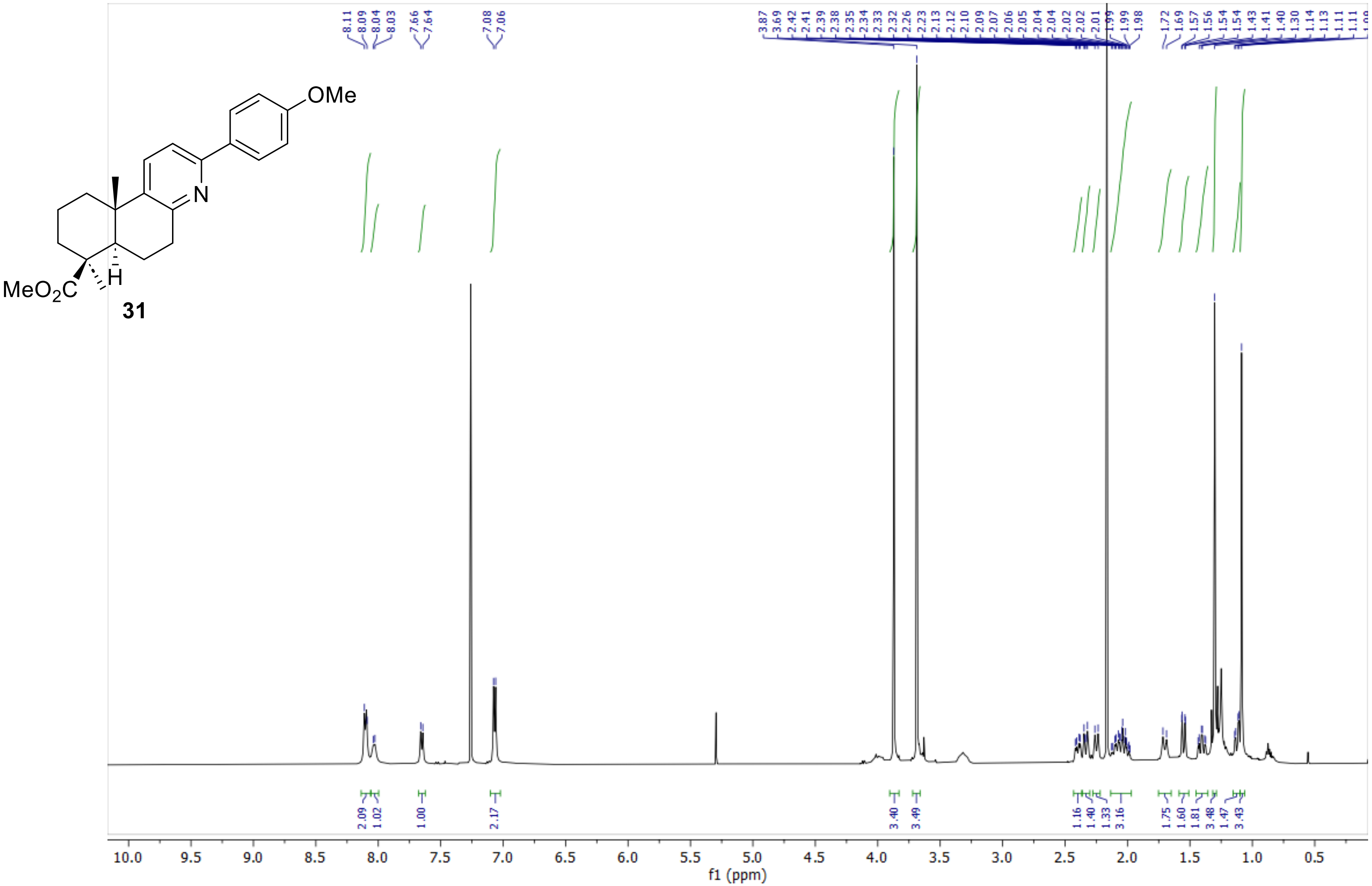

Figure S37. ^{1}H NMR spectrum of **31** ($CDCl_3$, 500 MHz)

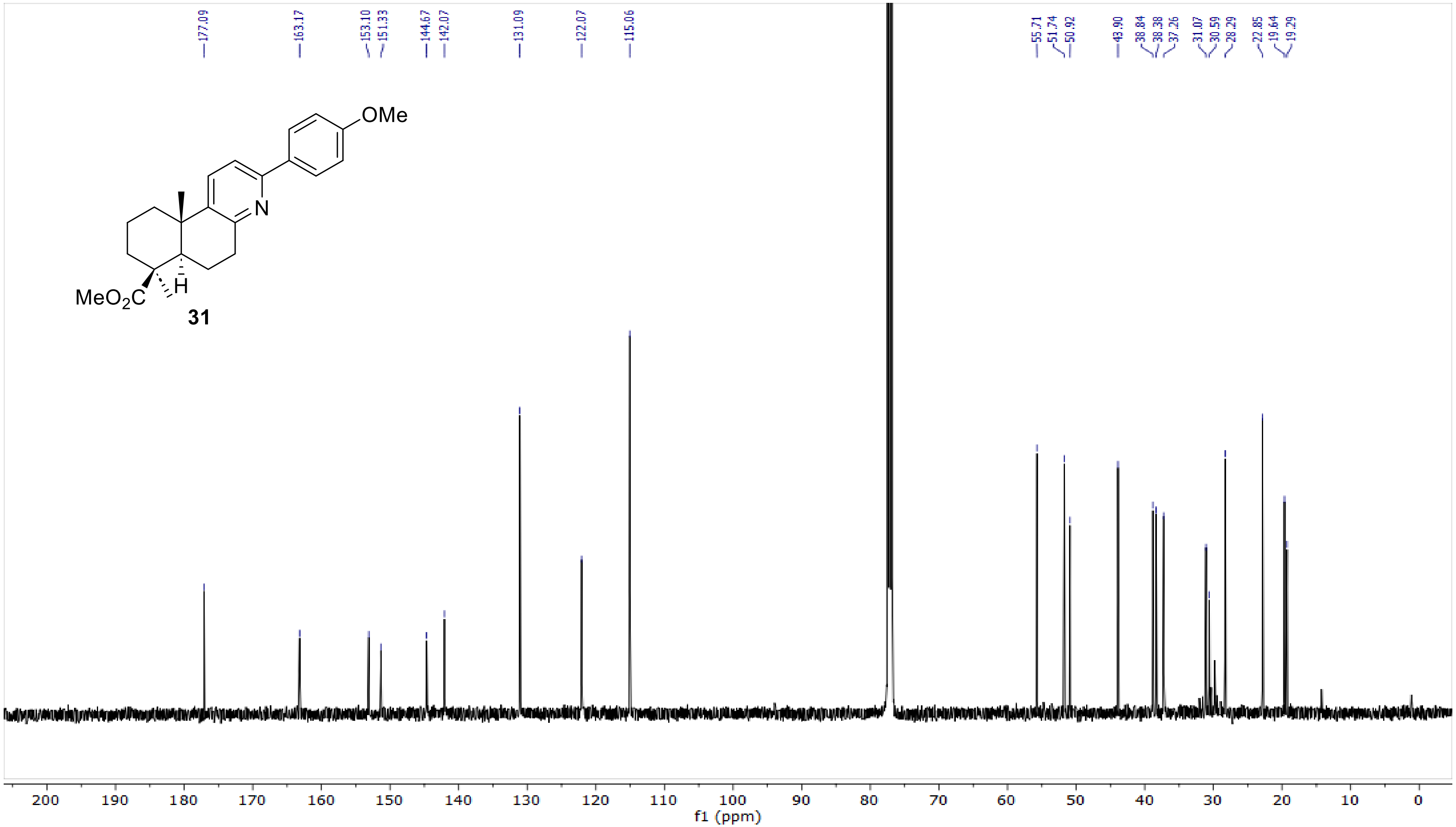

Figure S38. ^{13}C NMR spectrum of **31** ($CDCl_3$, 125 MHz).

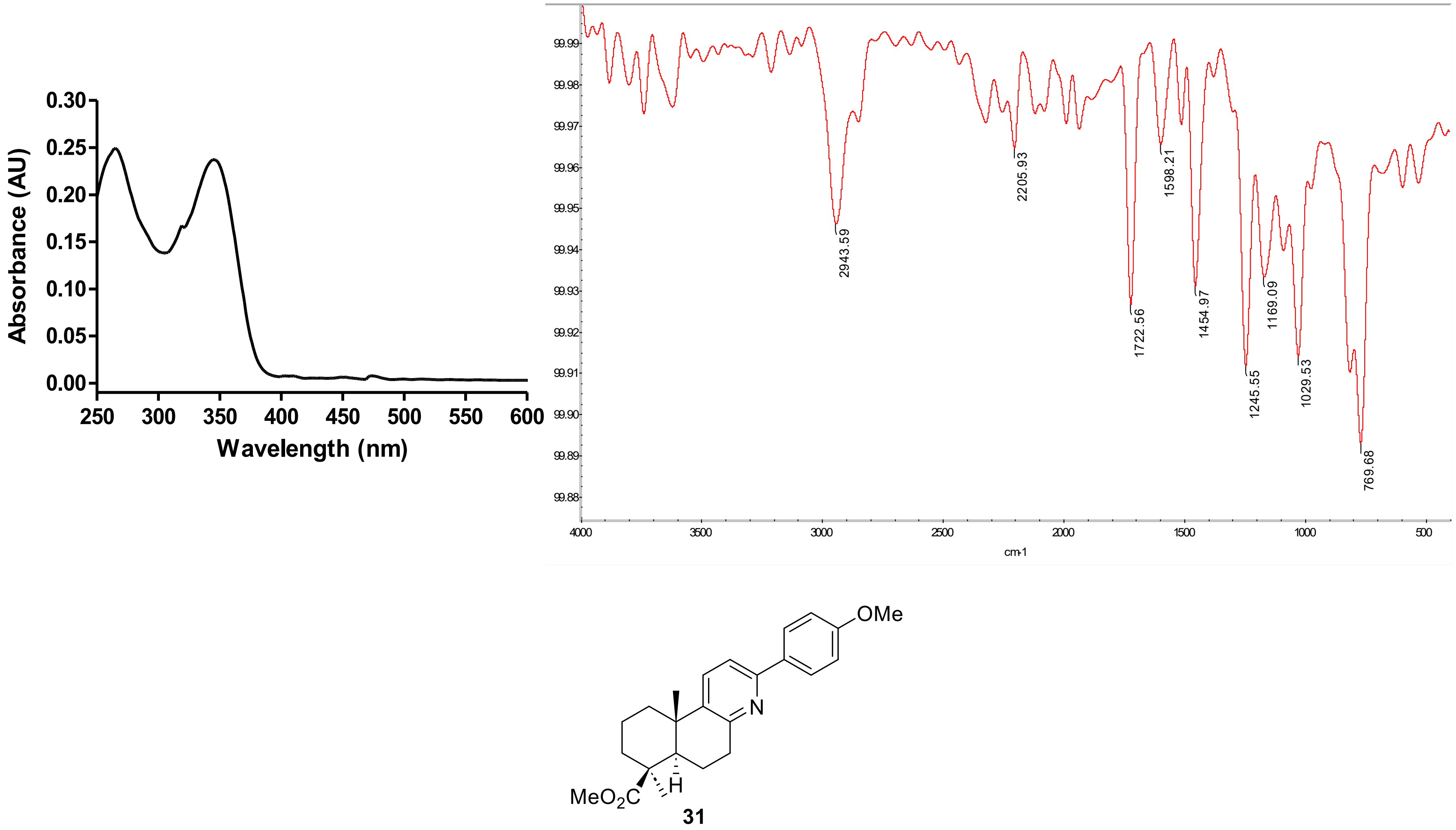

Figure S39. UV (in MeCN) and IR (KBr pellet) spectra report for **31**.

Elemental Composition Report

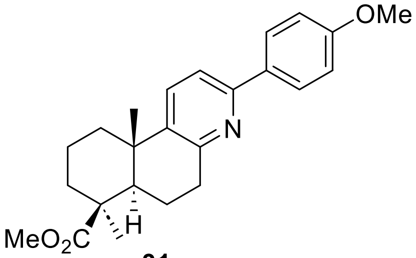

31

Single Mass Analysis

Tolerance = 5.0 PPM / DBE: min = -400.0, max = 400.0
Element prediction: Off
Number of isotope peaks used for i-FIT = 3

Monoisotopic Mass, Even Electron Ions
31 formula(e) evaluated with 1 results within limits (all results (up to 1000) for each mass)
Elements Used:
C: 23-25 H: 0-40 N: 0-1 O: 1-5 Na: 0-1

JJRac15 6.00000000
JJRac15 91 (3.454) Cm (79:99) 1: TOF MS ES+
3.62e+008

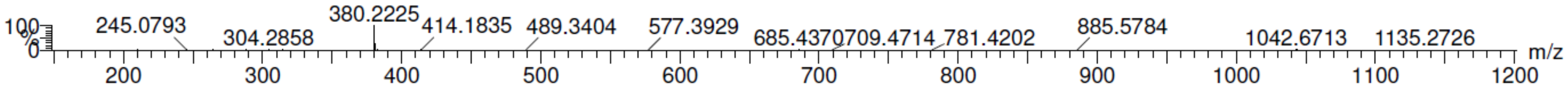

Minimum:				-400.0				
Maximum:		3.0	5.0	400.0				
Mass	Calc. Mass	mDa	PPM	DBE	i-FIT	Norm	Conf(%)	Formula
380.2225	380.2226	-0.1	-0.3	10.5	2535.4	n/a	n/a	C24 H30 N O3

Figure S40. HRMS report for **31**.
